# Epigenetically dysregulated genes and pathways implicated in the pathogenesis of non-syndromic high myopia

**DOI:** 10.1038/s41598-019-40299-x

**Published:** 2019-03-11

**Authors:** Sangeetha Vishweswaraiah, Joanna Swierkowska, Uppala Ratnamala, Nitish K. Mishra, Chittibabu Guda, Shiva S. Chettiar, Kaid R. Johar, Malgorzata Mrugacz, Justyna A. Karolak, Marzena Gajecka, Uppala Radhakrishna

**Affiliations:** 1 Department of Obstetrics and Gynecology, Oakland University William Beaumont School of Medicine, Royal Oak, MI USA; 2 Institute of Human Genetics, Polish Academy of Sciences, Poznan, Poland; 3 Department of Pharmacology, Creighton University, Omaha, NE USA; 4 Department of Genetics, Cell Biology & Anatomy College of Medicine, University of Nebraska Medical Center Omaha, Omaha, NE USA; 5 Department of Zoology, School of Sciences, Gujarat University, Ahmedabad, 380009 India; 6 Department of Ophthalmology and Eye Rehabilitation, Medical University of Bialystok, Bialystok, Poland; 7 Department of Genetics and Pharmaceutical Microbiology, Poznan University of Medical Sciences, Poznan, Poland

## Abstract

Myopia, commonly referred to as nearsightedness, is one of the most common causes of visual disability throughout the world. It affects more people worldwide than any other chronic visual impairment condition. Although the prevalence varies among various ethnic groups, the incidence of myopia is increasing in all populations across globe. Thus, it is considered a pressing public health problem. Both genetics and environment play a role in development of myopia. To elucidate the epigenetic mechanism(s) underlying the pathophysiology of high-myopia, we conducted methylation profiling in 18 cases and 18 matched controls (aged 4–12 years), using Illumina MethylationEPIC BeadChips array. The degree of myopia was variable among subjects, ranging from −6 to −15D. We identified 1541 hypermethylated CpGs, representing 1745 genes (2.0-fold or higher) (false discovery rate (FDR) p ≤ 0.05), multiple CpGs were p < 5 × 10^−8^ with a receiver operating characteristic area under the curve (ROC-AUC) ≥ 0.75 in high-myopia subjects compared to controls. Among these, 48 CpGs had excellent correlation (AUC ≥ 0.90). Herein, we present the first genome-wide DNA methylation analysis in a unique high-myopia cohort, showing extensive and discrete methylation changes relative to controls. The genes we identified hold significant potential as targets for novel therapeutic intervention either alone, or in combination.

## Introduction

Myopia, or nearsightedness, the most prevalent form of refractive error, is caused by excessive axial elongation of the eye as a major mechanism in children^1^. According to recent reports^2^, myopia is becoming an epidemic in the developed countries of East and South-East Asia, where the prevalence reaches 80–90% in children aged 17–18 attending secondary school. Concomitantly, the European Eye Epidemiology Consortium (E_3_) showed that the prevalence of myopia is also dramatically growing in the Western countries^3^.

A recent study by Holden *et al*. estimated the huge growth in the world population of myopia and high myopia, defined in his study as loss of 6.00 diopters (D) or more, as an increase from 1406 million and 163 million in 2000 to 4758 million and 938 million in 2050, for the two forms respectively^4^. These alarming data imply that by 2050 half of the world’s population may be affected by myopia, representing a significant social and economic burden to the global healthcare systems^5^.

The condition usually first appears between 8 to 12 years and as the child ages, vision can change rapidly, requiring a corrective prescription every few years. Myopia usually stabilizes by the age of approximately 20 years as the eyeball reaches its full size^6^. If myopia appears before the start of schooling (very early onset myopia), it is generally more severe and more likely to be genetic in origin. In addition, myopia developing at school age is less likely to progress to high myopia until the age of 11–13, and high myopia which appears after those ages is likely to be driven by environmental factors.

Around 30 distinct genetic risk loci have been identified for both high and mild myopia; however, the pathophysiology of variation(s) causing disease is obscure^7,8^. There is compelling evidence that both environmental and genetic factors are involved in the etiology of myopia^9–12^. Although environmental factors along with genetic predisposition are associated with the increasing prevalence of myopia amongst children, the mechanism through which they act is moderately understood. An epigenetic event such as DNA methylation could be one of the mechanisms through which these environmental factors influence the development of myopia. Previous studies have identified epigenetic analysis as a tool to reveal the causative mechanisms of ocular diseases including myopia^13,14^, but until now no genome-wide epigenetic association studies related to high myopia subjects have been reported. To elucidate potential epigenetic mechanism(s) underlying the pathophysiology of high myopia, we conducted a genome-wide methylation analysis on 18 high myopia subjects and an equal number of controls.

In this study, we investigated DNA methylation on a genome-wide scale using the Infinium MethylationEPICBeadChip-array technology in a unique cohort of children with non-syndromic high myopia, and identified associated biological pathways implicated in the development of high myopia. This study creates the preliminary awareness required to understand the influence of various factors that can contribute to the development of myopia.

## Results

### Differentially methylated CpG sites identification

**Figure 1:**
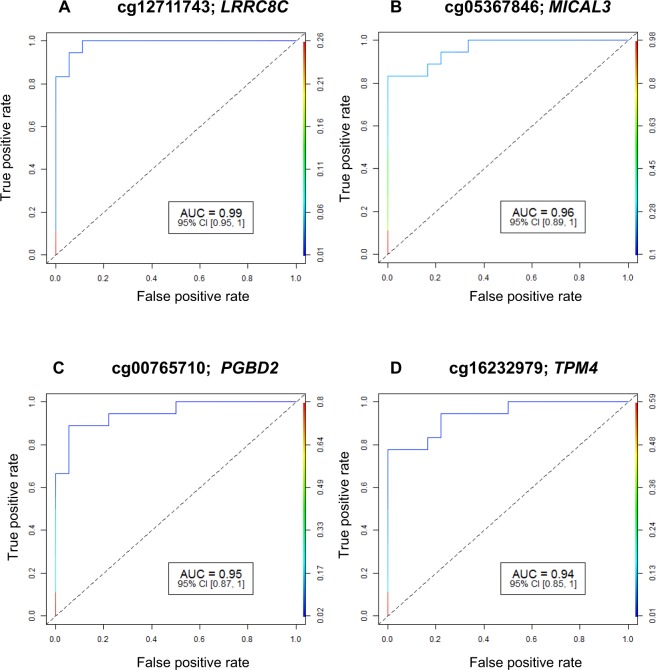
(**A**–**D**) Receiver operating characteristic curve analysis of methylation profiles. ROC analysis for four excellent CpG sites [(**A**) cg12711743: *LRRC8C*; (**B**) cg05367846: *MICAL3*; (**C**) cg00765710: *PGBD2*; and (**D**) cg16232979: *TPM4*]. At each locus, the FDR p-value for methylation difference between myopia subjects and controls was significantly different. ROC: Receiver operating characteristic; AUC: Area Under Curve; 95% CI: 95% Confidence Interval.

We identified 1541 CpG sites in 1745 unique genes that were differentially methylated (2.0-fold or higher) in high myopia subjects compared to controls without high myopia. All genes were hypermethylated and no CpG site was observed with significant hypomethylation. The detailed list of the most significant differentially methylated CpG sites based on FDR-corrected p-values, fold change and AUC for high myopia detection is shown in Supplementary Table S1. A total of 48 cytosine loci had excellent accuracy (AUC ≥ 0.90) for the detection of high myopia. A positive ‘% Methylation Change’ value indicates an average increase in methylation in high myopia subjects compared to control samples. The p-value indicates the significance of the differential methylation levels. Among the 1541 unique targets, the top 10 hypermethylated targets based on fold change were cg26526312 (*LINGO1*), cg27541540 (*PTPN11*), cg00609363 (*ZNRD1*), cg24877391 (*PEX13; PUS10*), cg21790796 (*KIF20A; BRD8*), cg14282407 (*TRAPPC1; CNTROB*), cg18191664 (*ERLIN2*), cg08048517 (*KIAA0528*), cg10646633 (*ZNF224*) and cg14694176 (*TCEA1*). The results for the four AUC ROC outcome measures are shown in Fig. 1. The study also identified 81 open reading frame (ORF) genes and 41 LOC genes associated with high myopia (Supplementary Tables S2 and S3). Both ORFs and LOC genes were highly significant between 2 to 7-fold difference and AUC value of >0.75 (FDR p-value < 0.01).

### Cluster analysis of differentially methylated targets

**Figure 2:**
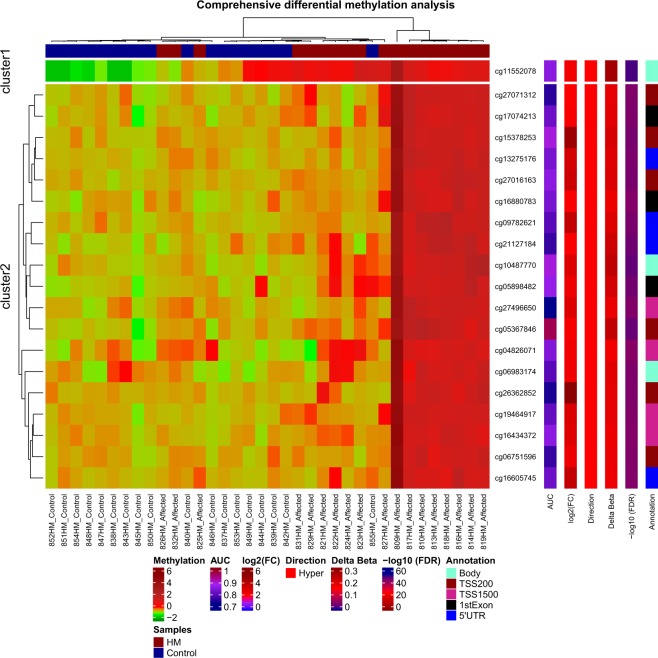
Heatmap and hierarchical clustering of myopia cases based on DNA methylation. Heat map showing hypermethylation variation in myopia cases compared with controls. The Heat map in hierarchical clustering analysis represented DNA methylation levels from completely methylated (red) to unmethylated (green).

**Figure 3:**
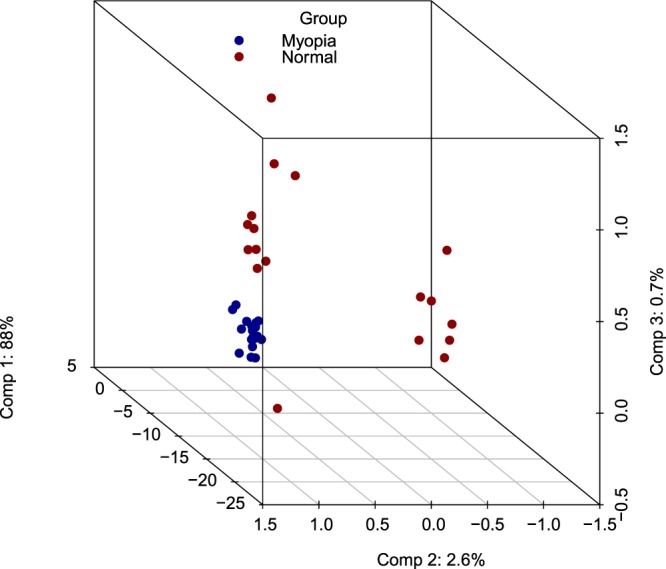
Three dimensional PCA (PCA 3D).

Hierarchical clustering was performed using β-values for commonly methylated genes that showed differential methylation between the high myopia group and controls. Twenty target genes significantly hypermethylated in myopic subjects comparing to the control group were used in the clustering analysis. These 20 targets were displayed in two distinct clusters, indicating that methylation was associated with altered expression of these genes in high myopia individuals (Fig. 2). PCA results showed complete separation between high myopia samples and control sample sets in the PCA distribution 3D plot (PCA 3D, Fig. 3). Cluster data correlates with PCA.

**Figure 4:**
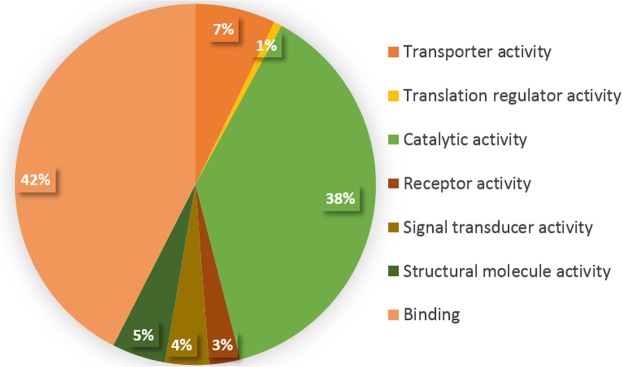
Functional enrichment analysis of differentially methylated genes involved in transporter activity, translation regulator activity, catalytic activity, receptor activity, signal transducer activity, structural molecule activity and binding activity with their respective percentages given.

**Figure 5:**
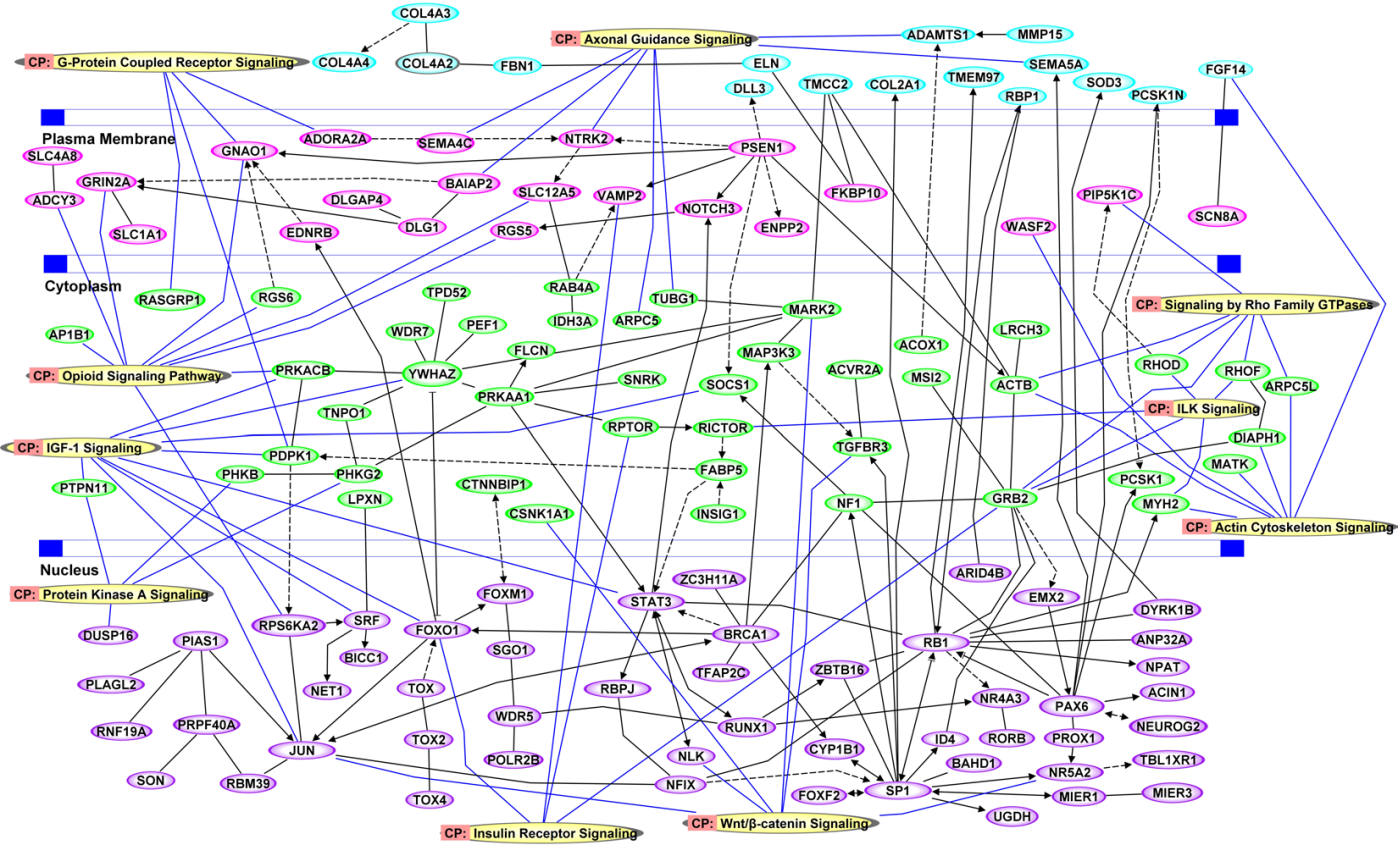
Pathways analysis of significant DNA methylation variations and network analysis performed using Ingenuity Pathway Analysis (IPA). Schematic location of nodes such as on extracellular space, plasma membrane, cytoplasm and nucleus is depicted.

Functional enrichment analysis of differentially methylated genes is shown in Fig. 4. Forty two percent of those genes are under the transcription binding activity category, followed by 38% genes with functional catalytic activity. The smallest category represents genes with translation regulator activity, comprising 1% of genes identified. Ingenuity Pathway Analysis identified 10 important canonical signaling pathways associated with high myopia gene enrichment at probability values ≤ 0.01. The identified canonical signaling pathways include: Wnt/β-catenin signaling, insulin receptor signaling, protein kinase A signaling, actin cytoskeleton signaling, ILK signaling, signaling by Rho family GTPases, IGF-1 signaling, the opioid signaling pathway, axonal guidance signaling, and G-protein coupled receptor signaling (Fig. 5).

## Discussion

Epigenetics plays a critical pathogenic role in the development of complex eye diseases including myopia^13^. Methylation profiling of DNA using array technology has been successfully used to explore CpG sites associated with various complex diseases^15,16^. Myopia is associated with number of other eye conditions such as cataracts^17^, glaucoma^18^, retinal detachment^19^, keratoconus^20^, macular degeneration, retinal holes, choroidal neovascularization^21^, diabetic retinopathy^22^ and retinitis pigmentosa^23^. Many studies have been performed to detect genes associated with myopia^24,25^. But studies on epigenetic factors, especially methylation studies, have not been performed to our knowledge. The current study identifies the global DNA methylation hotspots in the genome of high myopia patients under the age of 12 years.

We have identified differential methylation in genes those were previously suggested to be associated with myopia such as, *PAX6*, *ZNRF3*, *PSEN1*, *SOCS1*, *GRB2*, *ADCY3*, *RGS5*, *SRF* and *AP1B1*. Multiple variations of *PAX6* were previously shown to be associated with myopia^26,27^. *ZNRF3*, which was identified as one of the loci in the Consortium for Refractive Error and Myopia (CREAM) study, was also identified in the present study as a hypermethylated gene^28^. Mutations were previously observed in *PSEN1* in a patient with posterior cortical atrophy and myopia^29^ and we identified methylation on this gene in the present study. The differential expression of *SOCS1* has been observed in corneal cells of myopic patients^30^ and SOCS1 deficient mice develop severe eye diseases^31^. In RPE cells, SOCS1 higher expression inhibits IFNγ-mediated responses which leads to uveitis^32^. *GRB2* was found to be upregulated in chicks during imposed myopic defocus^33^ and also during retinal stress and neovascularization^34^. *ADCY3* and *RGS5* were previously noted to be upregulated in the retinal cells and scleral cells of myopic patients respectively, but without statistical significance^30^. *SRF* was shown to be upregulated in treated eyes versus control eyes of myopic guinea pigs^35^. A SNP (rs715494) within *AP1B1* gene, which is located in the MYP6 region, was previously identified among myopic patients^36^. These results indicate the reproducibility of our study in identifying markers for myopia.

### Oxidative stress

Oxidative stress is involved in myopia and other ocular diseases^37^. Besides inducing oxidative damage to various parts of eyes in myopia, high oxidative stress in various retinal structures leads to neovascularization in the choroid. Further, high myopia is associated with oxidative stress in various parts of eye in the development of glaucoma, cataract and retinal detachment. In the present study, we have identified several genes that are associated with regulation of oxidative stress including *PEX13*^38^, *NNT*^39^, *OXT*^40^, *SOD3*^41^, *PRDX1*^42^, *PRDX5*^43^, *CYB5B*, *CYB5R1*^44^ and *COQ3*^45^. Interleukin-1alpha downregulates SOD3 and the SOD3 reduction leads to insufficient oxidative defense during keratoconus condition^46^.

We have also identified NADH-ubiquinone oxidoreductase genes that are involved in oxidative phosphorylation process such as *NDUFA5*, *NDUFB2*, *NDUFB3*, *NDUFB7*, *NDUFB10*, *NDUFS8*, and *NDUFV2-AS1*^47^. Similarly, we have identified COX family member genes including *COX6B1*, *COX18*, and *COX16*; these take part in the physiological response to oxidative stress^48,49^. *ACOX1* also plays a role in oxidative damage^50^; *MCAT* is a mitochondrial-specific catalase enzyme^51^ and *PDIA4* expression increases with oxygen glucose deprivation^52^. *NADK2* is one of the enzymes that catalyzes phosphorylation of NAD(+) to yield NADP(+)^53^. Interestingly, *TRAF3IP2* and *JUN* genes were found to be hypermethylated in our study cases. *TRAF3IP2* is one of the oxidative stress-responsive cytoplasmic adapters that regulates c-JUN^54^.

### Axonal guidance

Axon guidance is vital for nerve growth in brain and sensory organs such as retina of eye. Retinal ganglion cells (RGC) project their axons to the visual cortex of the brain. There are number of proteins that act as axon guidance cues, such as netrins, semaphorins, slits, ephrins, L1CAM (L1), laminin, tenascin, chondroitin sulphate and Wnt proteins^55–58^. In high myopic individuals, thinning of the macular ganglion cell complex has been observed^59^. Lower macular thickness results in loss of RGCs and retinal nerve fibers^60^, resulting in disrupted signals to the visual cortex targets. Our study identified methylation differences in the semaphorins, such as *SEMA3F*, *SEMA4B*, *SEMA4C* and *SEMA5A* that were significantly associated with myopia. Among these, *SEMA5A* has been found to be specifically expressed during retinal axon outgrowth at the optic disc and along the optic nerve. Sema5A inhibits axon growth and retinal neurite outgrowth by retinal ganglion cells and Plexin family receptors respectively^61,62^. Even in the presence of growth-promoting axon guidance signaling molecules like laminin, L1 and netrins, the higher expression of *SEMA5A* could inhibit retinal growth cones^63^. Our data supports this mechanism for myopia, as we identified methylation at the gene body region that may support higher expression levels^64^ of *SEMA5A* and inhibition of retinal growth cones. *PAX6* was also identified to be hypermethylated by 3-fold at the 5’UTR in association with myopia in our study. Genetic variation at the regulatory region and other regions on *PAX6* expression is important for the development of axonal connections and *PAX6* has been reported to play a role in development of myopia^26,27^. The same study noted diminished expression of *SEMA5A*^65^. Neurotrophic Tyrosine Kinase, Receptor, Type 2 (*NTRK2*), also called as Tyrosine Kinase Receptor B (*TRKB*) is a receptor for Brain-Derived Neurotrophic Factor (*BDNF*), which plays a crucial role in the activity-dependent refinement of synaptic connectivity of retinal ganglion cells. The BDNF/TRKB complex is vital for development of photoreceptors, and for synaptic communication between photoreceptors and second order retinal neurons^66^. The current study observed methylation of *NTRK2* at the promoter region. Several other hypermethylated genes identified were *ADAMTS1*, *ARPC5*, *BAIAP2*, and *TUBG1*, for which further functional analysis is warranted. Among them, ADAMTS1 was previously found to be downregulated in corneas of keratoconus patients compared to control subjects^67^.

### Growth factor signaling and cell differentiation

Studies on animal models of myopia have suggested a role for several different growth factors and their signaling in the development of myopia. TGFβ increases in the sclera of form-deprived myopic animals, while levels of bFGF were found to be decreased^68^. The influence for the secretion of VEGF, HGF and IGF growth factors is derived from their role in oxidative stress in different parts of eye, specifically the retina^37^.

Most growth factors exert their effects on cells through G-protein coupled receptors (GPCRs)^69^. Our study identified certain signaling molecules to be associated with myopia. We have found hypermethylation of Regulator of G-protein signaling 2 (*RGS2*), which was earlier reported to be upregulated in the sclera of form deprivation myopia (FDM). Zou and collaborators supported therapeutic strategies to control dysregulated *RGS2* as a treatment for myopia^70^. *ADORA2A* was hypermethylated at the transcription start site in our study; this gene encodes the adenosine A2a receptor protein, which is a GPCR family member. *ADORA2A* is expressed in ocular tissues and modulates collagen synthesis and extracellular matrix production during eye growth. Exonic variants of this gene were previously identified in association with high myopia^71^. AdoRs showed to play role in growth regulation of eye and the variation in expression pattern of AdoR observed during form deprivation myopia confirms the pharmaceutical intervention may help in reducing myopia progression^72^.

In addition, we noted methylation alteration in the promoters of GPCR-associated Ras-regulating proteins such as *GNAO1*^73^, *PDPK1*, *RASGRP1*, *RASGRP2*, *RASGEF1B*, and *RASL10B*^74,75^. Among them, *RASGRP1* showed upregulation in human corneal epithelial cells and conjunctival epithelial cells upon IL-4 stimulation^76,77^. The Rho family of GTPases regulate growth and cellular transformation processes^78^. We found over 3-fold hypermethylation of promoters of two Rho family genes, *RHOD* and *RHOF*, to be significantly associated with myopia.

### Wnt signaling

The Wnt family of proteins lead one of the most versatile regulatory pathways in developmental biology, cell cycle and tissue homeostasis^79^. Substantial literature is available to support the association of Wnt signaling in the pathophysiology of progression of cancer, chronic progressive disorders, cell fate specification and differentiation related disorders^80^. Comprehensive study has now revealed that Wnt signaling is initiated through receptors (the Frizzled family) to downstream β-catenin^81^. Between these two ends many proteins interact in a complex pathway of signal transduction.

Wnt signaling is a ubiquitous pathway that is influenced by the action of various growth factors such as TGFβ, bFGF, IGF, VEGF, HGF, etc., and its components are critical for the development of ocular tissues at various stages, including the formation of eye, retina, lens, ciliary body, iris and vascular development^82,83^. Activation of Wnt signaling leads to the progression of myopia in mice. Wnt2b and Wnt3 along with β-catenin were shown to be highly expressed in scleral fibroblast cells in FDM^84,85^. In our study, we observed hypermethylation of Catenin Beta Interacting Protein 1 (*CTNNBIP1:* p = 0.006) and Catenin Alpha-Like 1 (*CTNNAL1*: p = 2.6 × 10^−38^) with 2.3 and 3.19-fold changes respectively. We identified 22 genes which were hypermethylated (*PPP3CA*, *RAC3*, *TCF7L1*, *CSNK2B*, *ROCK1*, *CTNNBIP1*, *PSEN1*, *BTRC*, *PPP3CC*, *TP53*, *AXIN1*, *PPP2R1B*, *CSNK1A1*, *TCF7L2*, *RBX1*, *FBXW11*, *TBL1XR1*, *NLK*, *PLCB4*, *PRKACB*, *JUN* and *SKP1*) and pathway analysis showed significant effects of these genes on pathway outcome, via both pathway and physical interactions (Supplementary Fig. 1A,B). Xion *et al*. had reported dysregulated mir-203 and mir-350 that have downstream implications on the Nlk, Sema5a and Acer2 genes, vital in the regulatory network^86^. Our study also observed hypermethylation in *NLK* and *SEMA5A*, with significant methylation change (p = 3.3 × 10^−38^ and p = 2.7 × 10^−9^, respectively). Both genes had greater than 2-fold changes in methylation as compared to control subjects. *NLK* is known to interact with *CTNNB1*, *TP53*, *TCF7L1*, and *TCF7L2*, which were hypermethylated as compared to controls.

We have identified differentially methylated genes under the influence of Wnt/β-catenin, including *JUN*, *MARK2*, *TGFBR3*, *NR5A2*, *SP1*, *NLK*, *CSNK1A1* and *ACVR2A* in myopic subjects. *JUN* is an oncogene shown to be upregulated in treated eyes versus control eyes of myopic guinea pigs^35^. Mitochondrial functioning is important for the refractive error in myopia^87^. Map/Microtubule Affinity-Regulating Kinase 2 (*MARK2*) has a strong role in maintaining the movement of mitochondria in retina/ganglion cells and helps in proper neuronal function^88^. *TGFBR3* is associated with Primary Open Angle Glaucoma (POAG)^89^ and high myopia is one of the risk factors for POAG^18^. The methylation identified in our study in the promoter and first exon of *TGFFBR3* may influence the expression of this gene, causing the myopic phenotype that may lead to POAG. The current study also shows hypermethylation on *NR5A2*, found to be linked to the *SP1* gene which is hypermethylated at the transcription start site. *SP1* is developmentally regulated and important for the corneal development^90^. The corneas in myopic patients tend to be thin compared to normal eyes^91^, implicating *SP1* and other network genes in maintaining corneal structure. Sp1 was shown to participate in regulating type I collagen synthesis or degradation during myopic sclera remodeling, in association with TGF-β1 signaling, suggesting a role in the development of myopia^92^. Another key gene in the regulatory network of Wnt signaling is *CSNK2B*, which showed significant (p = 0.01) hypermethylation with a 2.45-fold change as compared to control subjects. Kloss *et al*. reported a nonsynonymous damaging variant in *CSNK2B* (c.473 A > G resulting in p. Tyr158Cys) among high myopia families, resulting in interference with the Wnt signaling pathway^93^. Hypermethylation of *CSNK2B* may result in low levels expression, affecting interaction with *JUN*, *BTRC*, *PPP2R1B*, and *PPP3CC*.

Thus, overall diminished expression of key genes is likely to result in underpowered Wnt signaling, and consequently repressed expression of genes responsive to possible pathophysiology of the retina in myopia.

### Protein kinase A signaling activity

Protein kinase A signaling is an important process for retinal ganglion cell survival^94,95^. Hypermethylation in the 5’UTR of Dual-Specificity Phosphatase 16 (*DUSP16*) was significantly associated with myopia in our study. MAPKs regulate insulin sensitivity^96^, which is an activator of retinal transmitters resulting in eye growth^97^. Dual-specificity phosphatases inactivate MAPKs by dephosphorylation^98^. In addition, *MAPK3* was also observed to be under a hypermethylation burden in our study. Several other genes seen to be hypermethylated are under protein kinase A signaling control, including *PHKB*, *PHKG2*, *PTPN11* and *CDC27*. Among these, *PTPN11* plays a role in lens and retinal development^99,100^ and found to be associated with myopia^7^, and two other hypermethylated genes in our study, *FBN1* and *OCA2*, were also found to be associated with myopia in the same study^7^ and *FBN1* is associated with refractive error^101^.

### IGF-1 Signaling

IGF has been shown to be associated with myopia in a chick model. Insulin injections block hyperopia and induce axial myopia in chicks most likely because of its influence on the optics of the anterior segment of eye^102^. Forkhead Box O1A (*FOXO1*) is one of the major targets of insulin action^103^ and was hypermethylated at the transcription start site in the present study. This gene was previously identified in association with reduction of central corneal thickness^104^ and keratoconus^105^, a condition that exhibits increased axial length, which has significant relationship with axial myopia^20^. *RPTOR* and *VAMP2* are two more genes identified in our study under insulin receptor signaling and were hypermethylated with a significant association with myopia.

The altered secretion of various growth factors leads to activation of intracellular signaling pathways such as MAPK/Erk, Wnt/β-catenin and PI3K/Akt^97,106^ and ultimately leads to altered organization of the cytoskeleton and secretion of extracellular matrix components. The actin cytoskeleton provides mechanical force for cell movement and division^107,108^. We have identified many genes in the canonical actin cytoskeleton pathway that are statistically significantly associated with high myopia. These are *ACTB*, *MATK*, *MYH2*, *RB1*, *DIAPH1*, *FGF14*, *ARPC5L and WASF2I*. Among them, *MYH2* mutations are associated with ophthalmoplegia (paralysis or weakness of eye muscles)^109^. *RB1* is associated with retinoblastoma^110^.

The *LINGO1* gene is involved in actin dynamics^111^ and we found its promoter to be hypermethylated by 11-fold at the 5’UTR. *ACTB* codes for beta-actin, and in the presence of actin cue netrin-1, the translation of β-actin is initiated in the RGC growth cones^112^, suggesting the importance of this gene in maintenance of retinal structure.

Additional genes related to myopia and identified in this study are *MATK* that has shown differential expression in the retinal cells of myopic patients^30^. *STAT3* signaling is critical for scleral remodeling and myopia development^113^, *COL2A1* and *BICC1* are candidate genes for myopia^114,115^.

In summary, understanding various cellular and molecular pathways altered in patients with high myopia is essential in any attempts to block myopic growth, and hence can be useful in advancing treatment modalities for myopia. We have found different functional groups of genes that are significantly hypermethylated in the peripheral blood cells of high myopia patients. Whereas, we confirmed several previously reported candidate genes for myopia, many genes were newly identified with unknown mechanisms which further warrant functional studies. The replication of candidate gene association seen in this study adds to the weight of evidence that epigenetic methylation may be responsible for the development of myopic features seen in young children. In-depth bioinformatics network and pathway analysis identified significantly associated canonical pathways with eye development and function, allowing putative biological meaning to be assigned to the genes identified in the study. These genes were correlated to retinal ganglion cell development and maintenance, axial length difference, synaptic communication, corneal and scleral dysfunction. The pathway analysis also revealed possible interaction among the identified genes that may be involved in these mechanisms. One of the drawbacks of the study is that this analysis used blood DNA and not ocular tissue DNA. Nevertheless, this study provides confirmatory and novel data that should prompt further studies to address the functional role of the identified hypermethylated genes in the development of high myopia.

We proposed that methylation studies could help us to identify the genes associated with high myopia. Associated pathway analysis could provide insight into the mechanism of the development of myopia and might also help develop the preliminary awareness required to deter the influence of various factors contributing to the development of myopia. Identifying genes that contribute to the etiology of high myopia and elucidating the associated molecular mechanism(s) is the first step toward understanding the pathophysiology of this disease. This study details some of the etiology of myopia and eye growth patterns and thus has considerable public health application, as the high prevalence of myopia in the world is a formidable health care challenge. Furthermore, the identification of myopia-causing genes will be important for improving early myopia diagnosis and counseling and increasing our understanding of normal and abnormal development of the eye.

## Methods

The present study was performed on the 18 Polish high myopia cases with refractive error ranging from −6.0 to −15.0 D in at least one eye with the axial length of the eye ranging from 26.22 mm to 27.85 mm (mean, 26.22 mm) and 18 controls without high myopia, with the axial length of the eye ranging from 22, 42 mm to 24.11 mm (mean, 22, 55 mm) with same ethnicity, age and gender. All cases were recruited at Department of Pediatric Ophthalmology Medical University of Bialystok and were aged between 4–12 years at the time of sample collection. All children had a comprehensive eye examination, including cycloplegic (cyclopentolate 1%) autorefraction to assess refractive error and ocular biometry to measure axial length of the eye. In all cases, the anterior segment of the eye, including cornea and lens, were normal. Clinical demographics are available for each high myopia subject of the study (Table 1). All subject identities were masked during experimental processing and analysis. This study was approved by the Institutional Review Boards at Poznan University of Medical Sciences in Poland and the informed consent was given by each subject’ parents, according to the Declaration of Helsinki. All methods were performed in accordance with the relevant guidelines and regulations.

**Table 1:** Demographics and clinical characteristics of high myopia cases included in the present study.

Individual ID	Age	Right Eye				Left Eye			
		SRE [D]	CRE [D]	Ax [0]	SE [D]	SRE [D]	CRE [D]	Ax [0]	SE [D]
UR-809	4	−11.50	+1.50	132	−10.75	−13.0	+2.0	118	−12.00
UR-810	10	−6.75	+1.00	90	−6.25	−7.50	+1.50	80	−6.75
UR-813	11	−6.25			−6.25	−5.5			−5.50
UR-814	11	−9.5	+0.75	5	−9.25	−9.25			−9.25
UR-816	10	−5.50			−5.50	−6.25			−6.25
UR-817	11	−7.00	+4.00	90	−5.00	−5.75	+4.50	90	−3.50
UR-818	12	−10.25	+1.50	90	−9.50	−10.50	+1.50	90	−9.75
UR-819	12	−7.5			−7.50	−7.0			−7.00
UR-821	5	−7.00	+1.50	5	−6.25	−10.00	+1.50	175	−9.25
UR-822	11	−7.00			−7.00	−7.0			−7.00
UR-823	12	−6.00			−6.00	−6.5			−6.50
UR-824	12	−2.75			−2.75	−6.50	+0.75	110	−6.25
UR-825	9	−15.00	+4.25	175	−13.00	−14.00	+2.50	5	−12.75
UR-826	11	−9.25			−9.25	−8.5			−8.50
UR-827	9	−6.50			−6.50	−6.5			−6.50
UR-829	9	−10.00	+1.00	170	−9.50	−8.25	+2.25	30	−7.25
UR-831	9	−10.0	+3.0	170	−8.50	−7.75	+2.75	10	−6.50
UR-832	12	−6.50			−6.50	−8.5			−8.50

SRE- Spherical refractive error, CRE- Cylindrical refractive error, SE- Spherical equivalent, Ax- axis of CRE.

Note: Other eye diseases: UR-809 strabismus, UR-817 retinal detachment, UR-829 retinopathy of prematurity.

### Genome-wide methylation analysis

Genomic DNA from peripheral blood samples was extracted using Gentra Puregene Blood Kit (Qiagen, Hilden, Germany) according to the manufacturer’s protocol. DNA (500 ng) was bisulfite converted using the EZ DNA Methylation-Direct Kit (Zymo Research, Orange, CA) according to the manufacturer’s instruction.

Infinium MethylationEPIC BeadChip arrays (Illumina, Inc., California, USA) with 850,000 methylation sites were used for the analysis of genome-wide methylation according to manufacturer’s protocol. The methylation sites are distributed over regions including transcriptional start sites (TSS200, TSS1500), promoters, 5′UTR, exon boundaries, coding, and 3′UTR regions of autosomes and allosomes. Fluorescently-labeled BeadChips were imaged using iScan (Illumina, Inc.). Quality control, data preprocessing and background signal intensity correction was performed to yield a ratio of methylated and unmethylated signal intensities, followed by detailed bioinformatic and statistical analyses. 99% of the CpG loci were determined unequivocally and all the processing was done according to manufacturer’s instructions.

### Bioinformatic and Statistical analysis

Gene-specific, genome-wide DNA methylation was quantified using the GenomeStudio methylation package (Illumina Software). DNA methylation ß-values were assigned to each CpG site. Differential methylation was identified by comparing these ß-values per individual nucleotide at each CpG site, between affected cases and normal controls. To avoid potential confounding factors, probes containing SNPs or within 10 bp of CpG sites listed on dbSNP entries were excluded from further analysis, as these interfere with determination of methylation at the corresponding sites^116–119^. Probes with SNPs over 10 bp from methylation sites or with an allelic frequency of ≤0.005 were further considered along with the remaining methylation sites for the analysis.

The fold change in CpG site variations were obtained by dividing the mean ß-values of the probes in each island by normal controls. Differentially methylated CpG sites were defined by the pre-set cutoff criteria of ≥2.0-fold increase and/or ≥2.0-fold decrease with False Discovery Rate (FDR) *p* < 0.05. Receiver Operating Characteristic (ROC) curves were generated for each CpG site, and the corresponding area under the curves (AUCs) were calculated to quantify diagnostic markers. To avoid potential experimental confounding, various statistical modeling was used. A heatmap was generated for the differentially-methylated genes, using ComplexHeatmap (v1.6.0)^120^ R package (v3.5.0). Ward’s minimum variance^121^ was used for the hierarchical clustering of samples. A multiple logistic regression analysis was done using more stringent criteria (FDR *p* ≤ 0.00001 and ≥2-fold change), to select candidate genes for high myopia biomarkers.

### Principal Component Analysis (PCA)

Prior to analysis, we removed all CpG-probes with missing ß-values; the remaining ß-values of CpG targets were used for PCA. We analyzed PCA using the R function “prcomp” and used PC1, PC2 and PC3 for the PCA sharing plot. The 3D PCA distribution plot was created by using R package “ggplot2”. All CpG variables of myopic cases and controls were computed together to detect variations between myopia and controls.

### Validation of methylation status by bisulfite pyrosequencing

Pyrosequencing was performed to verify the variable methylation status of important methylation variants, to determine that CHIP hybridizations were not artifacts but the true methylation differences. Forty-eight targets with AUC ≥ 0.9 were chosen for validation, comprising the 53 genes with the most significant methylation variation (p < 0.00001). 2 µg of genomic DNA was bisulfite-treated using the EZ methylation kit (Zymo Research) per the manufacturer’s instructions and sequencing was performed with appropriate oligos using the PyroMark Q24 System and advanced CpG reagents (Qiagen). An additional analysis replicated the top-25 differently methylated CpG sites in an independent second cohort of 24 high myopia cases and matched 24 controls and confirmed the top-ranking differentially-methylated CpG sites in whole blood DNA of our cohort samples.

### Gene functional enrichment and pathway analysis

Genes found to be differentially methylated (FDR p-value < 0.00001) were subjected to functional enrichment analysis using the PANTHER Classification System^122^. Gene networks and pathways were analyzed using Ingenuity Pathway Analysis (IPA) software (Qiagen) to identify biological functions or interacting regulatory networks. All CpGs without mapping identifiers in IPA (GRCh37/hg19) were excluded from analysis. Only genes for which Entrez identifiers were available were further analyzed. Further, to understand the gene networks better, we performed gene enrichment analysis using WEB-based GEne SeT AnaLysis Toolkit and network analysis using GeneMANIA.

## Supplementary information

Dataset 1

## Data Availability

All data is appended in the manuscript.
