# Supplementary material for: Epigenetically dysregulated genes and pathways implicated in the pathogenesis of non-syndromic high myopia
Source: Sci Rep. 2019 Mar 11;9:4145. doi: 10.1038/s41598-019-40299-x (PMC6411983; doi:10.1038/s41598-019-40299-x)

Supplementary file

Title: **Epigenetically dysregulated genes and pathways implicated in the pathogenesis of non-syndromic high myopia**

Sangeetha Vishweswaraiah[1], Joanna Swierkowska[2], Uppala Ratnamala[3], Nitish K Mishra[4], Chittibabu Guda[4], Shiva S Chettiar[5], Kaid R Johar[5], Malgorzata Mrugacz[6], Justyna A. Karolak[7], Marzena Gajecka[2,7,] Uppala Radhakrishna[1]

1. Department of Obstetrics and Gynecology, Oakland University William Beaumont School of Medicine, Royal Oak, MI, USA
2. Institute of Human Genetics, Polish Academy of Sciences, Poznan, Poland
3. Department of Pharmacology, Creighton University, Omaha, NE, USA
4. Department of Genetics, Cell Biology & Anatomy College of Medicine, University of Nebraska Medical Center Omaha, NE, USA
5. Department of Zoology, School of Sciences, Gujarat University, Ahmedabad-380009, India
6. Department of Ophthalmology and Eye Rehabilitation, Medical University of Bialystok, Bialystok, Poland
7. Department of Genetics and Pharmaceutical Microbiology, Poznan University of Medical Sciences, Poznan, Poland

[1]To whom correspondences should be addressed.

Email: Uppala.Radhakrishna@beaumont.edu

The authors declare no competing financial interests.

Supplementary Material headings

Supplementary Table S1. Details of CpG targets significantly differentially methylated in myopia. Target ID, Gene ID, chromosome, FDR p-value, fold change, % methylation and AUC details are given

Supplementary Table S2. Target ID, Gene ID, chromosome, FDR p-value, fold change, % methylation and AUC details of Open Reading Frames (ORF)

Supplementary Table S3. Target ID, Gene ID, chromosome, FDR p-value, fold change, % methylation and AUC details of LOC genes

Supplementary Figure 1A. Wnt gene interaction network based on pathway prediction

Supplementary Figure 1B. Wnt gene interaction network based on physical interaction

Supplementary Table S1:

Target ID	CHR	Genes	FDR p-Val	Fold change	% Methylation		AUC	CI	
					Cases	Controls		lower	upper
cg26526312	15	LINGO1	1.90898E-39	11.06	11.75	1.06	0.83	0.69	0.96
cg21790796	5	KIF20A; BRD8	4.0599E-38	7.80	8.62	1.11	0.85	0.73	0.98
cg03902565	5	NNT	3.3224E-38	6.63	9.10	1.37	0.85	0.73	0.98
cg24745548	2	GMPPA	3.35327E-39	6.17	12.07	1.96	0.78	0.63	0.94
cg15388430	22	POLDIP3; RNU12	1.98883E-06	6.14	4.26	0.69	0.81	0.67	0.96
cg21630368	11	CREBZF	2.01866E-09	6.04	5.47	0.91	0.83	0.69	0.97
cg23316161	12	ZNF605	2.96846E-06	5.98	4.21	0.70	0.78	0.63	0.93
cg14421548	9	RORB	1.06967E-37	5.86	7.79	1.33	0.82	0.69	0.96
cg14700647	10	ASAH2B	1.42318E-07	5.76	4.82	0.84	0.79	0.64	0.94
cg00115101	1	THRAP3	4.25325E-06	5.61	4.22	0.75	0.77	0.62	0.93
cg11048311	10	UPF2	1.39971E-06	5.41	4.48	0.83	0.77	0.62	0.93
cg01263292	12	IRAK3	2.73835E-05	5.40	3.88	0.72	0.80	0.65	0.95
cg09442143	9	ERCC6L2	3.58535E-38	5.13	9.50	1.85	0.80	0.66	0.95
cg22547764	3	ETV5	9.62643E-39	5.06	11.26	2.22	0.75	0.59	0.91
cg25320221	1	SMG7	1.95586E-14	5.01	7.71	1.54	0.89	0.78	1.00
cg18649601	1	ZC3H11A	2.2453E-38	4.98	10.19	2.05	0.81	0.67	0.95
cg12725906	1	ARPC5; RGL1	3.06815E-07	4.85	4.95	1.02	0.81	0.67	0.96
cg19125926	11	TMEM25	2.38802E-40	4.83	16.05	3.32	0.79	0.64	0.94
cg10115182	2	PSMD14	8.88106E-13	4.82	7.22	1.50	0.82	0.69	0.96
cg10413944	2	AP1S3	4.51031E-11	4.80	6.57	1.37	0.80	0.66	0.95
cg00662647	1	SLC35F3	7.54948E-10	4.80	6.08	1.26	0.81	0.67	0.96
cg05966809	5	SNX24	9.14296E-15	4.63	8.12	1.75	0.77	0.62	0.93
cg13745645	15	FAM189A1	0.00011697	4.62	3.76	0.81	0.83	0.69	0.96
cg21054968	7	INSIG1	6.44803E-39	4.53	12.13	2.68	0.85	0.72	0.98
cg05983883	8	POLR2K	0.000184995	4.51	3.68	0.82	0.77	0.61	0.92
cg11944815	12	DHX37	3.22544E-05	4.51	4.09	0.91	0.83	0.70	0.97
cg18188134	16	PHLPP2	8.91907E-38	4.50	8.56	1.90	0.75	0.59	0.91
cg15721164	14	MNAT1	2.03334E-38	4.48	10.62	2.37	0.90	0.80	1.00
cg21393421	1	RPL22	3.54887E-07	4.31	5.15	1.20	0.76	0.60	0.92
cg04226724	1	LPHN2	8.33343E-07	4.30	4.98	1.16	0.78	0.63	0.93
cg06404175	20	OXT	5.19511E-42	4.27	21.20	4.97	0.89	0.77	1.00
cg20597714	1	SLC35E2	0.000508488	4.27	3.50	0.82	0.81	0.67	0.96
cg18947032	10	KIAA1279	3.07998E-38	4.17	10.27	2.46	0.81	0.66	0.95
cg17341933	17	BAIAP2; FLJ90757	8.09852E-08	4.17	5.53	1.32	0.77	0.62	0.93
cg00167654	17	WSCD1	4.60365E-38	4.15	9.72	2.34	0.76	0.60	0.92
cg19354091	18	SERPINB8	2.89987E-08	4.13	5.77	1.40	0.84	0.70	0.97
cg19767580	16	CYLD	1.28021E-12	4.09	7.70	1.88	0.78	0.63	0.94
cg05996236	5	ZNF354B	9.50019E-39	4.08	11.99	2.94	0.84	0.71	0.97
cg24942285	3	DHX30	3.18786E-10	4.08	6.69	1.64	0.78	0.63	0.94
cg12166292	2	NABP1	0.000430086	4.06	3.62	0.89	0.78	0.63	0.93
cg13758646	9	GABBR2	0.034892886	4.05	2.28	0.56	0.80	0.65	0.95
cg00439706	15	MGA	3.42498E-09	4.04	6.26	1.55	0.77	0.62	0.93
cg14757267	19	RPL18AP3; RPL18A	2.2053E-07	4.03	5.41	1.34	0.78	0.62	0.93
cg09552183	12	ZNF84	6.02736E-07	4.01	5.21	1.30	0.80	0.65	0.94
cg23799720	16	WWOX	8.4541E-12	4.00	7.44	1.86	0.82	0.69	0.96
cg05984388	16	MAPK3	4.09409E-15	4.00	8.89	2.22	0.82	0.69	0.96
cg22762844	2	MAL	2.36814E-38	3.98	10.80	2.71	0.79	0.64	0.94
cg15708001	12	CBX5; HNRNPA1	7.84728E-05	3.98	4.09	1.03	0.82	0.68	0.96
cg04829746	11	LPXN; CNTF; ZFP91	0.000166056	3.98	3.90	0.98	0.80	0.66	0.95
cg15304692	7	REPIN1	7.19024E-09	3.96	6.17	1.56	0.80	0.66	0.95

cg14109663	19	HSD11B1L	1.54679E-06	3.95	5.03	1.27	0.86	0.73	0.98
cg12380909	8	NKAIN3	2.72314E-14	3.95	8.54	2.16	0.77	0.61	0.92
cg08261878	2	IHH	2.38278E-07	3.95	5.44	1.38	0.85	0.72	0.98
cg03293513	16	NME3	1.27745E-10	3.94	6.98	1.77	0.79	0.64	0.94
cg19336883	11	PRDX5; TRMT112	4.68764E-38	3.94	9.86	2.50	0.92	0.82	1.00
cg03250058	1	CHTOP; S100A13	7.79215E-05	3.91	4.12	1.05	0.80	0.65	0.95
cg05659223	19	RFX1	0.000433127	3.91	3.68	0.94	0.75	0.58	0.91
cg16337295	6	HSP90AB1	0.003016453	3.89	3.13	0.80	0.90	0.79	1.00
cg18414379	12	RNF10	0.005533233	3.83	2.96	0.77	0.89	0.77	1.00
cg12430845	5	NIPBL	1.52094E-07	3.82	5.63	1.47	0.84	0.70	0.97
cg07023902	2	INO80B	6.28031E-06	3.82	4.79	1.25	0.76	0.60	0.92
cg20960422	2	SLC39A10	1.3713E-13	3.81	8.42	2.21	0.78	0.63	0.93
cg10610482	11	ATM; NPAT	6.6139E-10	3.80	6.79	1.79	0.77	0.62	0.93
cg26215849	13	MTRF1	3.91586E-42	3.79	22.40	5.91	0.81	0.67	0.95
cg04017116	1	RPAP2; GLMN	1.69136E-06	3.78	5.12	1.36	0.81	0.66	0.95
cg09037869	4	SMIM20	1.21727E-13	3.75	8.52	2.27	0.77	0.62	0.93
cg23246901	12	SLC24A6	8.28851E-42	3.75	21.58	5.76	0.86	0.74	0.99
cg19905754	4	ANKRD37	0.008795052	3.74	2.84	0.76	0.80	0.65	0.95
cg27516438	19	SSBP4	7.26312E-39	3.63	12.88	3.55	0.87	0.75	0.99
cg10563352	2	COL4A3; COL4A4	1.45291E-05	3.63	4.71	1.30	0.76	0.60	0.92
cg20942339	12	SCN8A	5.82378E-11	3.61	7.49	2.08	0.79	0.64	0.94
cg14809226	17	HIC1	2.84422E-08	3.60	6.18	1.72	0.93	0.84	1.00
cg09961571	19	MRPS12; SARS2	3.37299E-41	3.60	20.15	5.59	0.88	0.76	1.00
cg21194937	19	AP3D1	5.37231E-07	3.60	5.52	1.53	0.87	0.75	0.99
cg04127233	4	CORIN	2.12984E-38	3.57	11.40	3.20	0.86	0.74	0.99
cg05173517	12	RHOF	2.14545E-09	3.56	6.79	1.91	0.87	0.75	0.99
cg25022560	1	SLC41A1	0.021476776	3.56	2.59	0.73	0.77	0.62	0.93
cg14622441	17	RASL10B	3.98916E-12	3.56	8.10	2.28	0.76	0.60	0.92
cg05476169	22	CBY1	0.001025668	3.56	3.59	1.01	0.77	0.62	0.93
cg03589372	16	BCKDK	3.39741E-06	3.55	5.12	1.44	0.79	0.64	0.94
cg19808285	20	RALGAPA2	1.74505E-05	3.55	4.71	1.33	0.85	0.72	0.98
cg22261911	6	AGPAT1	0.002385205	3.54	3.34	0.94	0.79	0.64	0.94
cg12102151	20	SLMO2	8.34531E-09	3.54	6.52	1.84	0.81	0.67	0.96
cg11026330	4	LCORL	2.47674E-08	3.51	6.31	1.79	0.75	0.58	0.91
cg06699275	11	MTL5	0.001021569	3.51	3.62	1.03	0.90	0.79	1.00
cg18709605	12	FICD	2.2029E-07	3.49	5.83	1.67	0.78	0.62	0.93
cg26029682	1	PTGFRN	6.85582E-38	3.48	9.75	2.80	0.81	0.67	0.96
cg21704267	15	STARD9	9.0534E-07	3.48	5.51	1.58	0.82	0.69	0.96
cg07358015	8	INTS9; HMBOX1	3.32883E-12	3.48	8.25	2.37	0.82	0.69	0.96
cg19515530	4	UBA6	1.17606E-07	3.47	5.99	1.73	0.80	0.65	0.95
cg02121891	3	BBX	3.90866E-13	3.47	8.71	2.51	0.87	0.75	0.99
cg08278991	10	NDST2	1.133E-07	3.45	6.03	1.75	0.77	0.61	0.92
cg14933266	17	NF1	0.001266904	3.44	3.59	1.04	0.82	0.68	0.96
cg24443885	19	ZNF227	4.43326E-05	3.43	4.56	1.33	0.86	0.73	0.98
cg07205462	20	SNRPB2	2.55412E-12	3.38	8.46	2.50	0.90	0.80	1.00
cg00510859	2	SLC30A3	7.35649E-07	3.38	5.65	1.67	0.78	0.63	0.93
cg17649531	2	NCL	0.000628147	3.36	3.86	1.15	0.78	0.63	0.93
cg15687240	1	RRAGC	6.52701E-05	3.35	4.52	1.35	0.78	0.63	0.94
cg24626312	17	HOXB5	5.46473E-05	3.35	4.57	1.36	0.79	0.64	0.94
cg23073439	6	KDM1B; TPMT	5.40498E-10	3.34	7.38	2.21	0.81	0.67	0.95
cg24040382	1	WDR26	1.49178E-07	3.33	6.09	1.83	0.79	0.64	0.94
cg25544621	8	LRRC14; RECQL4	1.27315E-08	3.33	6.68	2.01	0.75	0.59	0.91
cg16349255	15	MTFMT	0.000338982	3.32	4.06	1.22	0.80	0.65	0.94
cg07002382	15	MFAP1	5.16207E-06	3.31	5.23	1.58	0.75	0.58	0.91

cg27016163	2	HOXD13	1.14038E-43	3.29	27.97	8.50	0.86	0.74	0.99
cg18500286	4	AFF1	3.77571E-15	3.29	9.91	3.01	0.79	0.64	0.94
cg08739233	1	SHISA4	0.000143946	3.28	4.34	1.32	0.76	0.60	0.92
cg13710595	15	BAHD1	0.000807059	3.26	3.84	1.18	0.77	0.62	0.93
cg06862673	17	CDK12	2.89426E-41	3.21	21.35	6.66	0.91	0.81	1.00
cg08869036	8	RDH10; RPL7	0.003213262	3.20	3.43	1.07	0.79	0.64	0.94
cg21070498	1	WDR78; MIER1	0.0001871	3.19	4.34	1.36	0.77	0.62	0.93
cg15042246	8	CA2	0.039701042	3.18	2.49	0.78	0.79	0.64	0.94
cg00032336	17	TTC19; ZSWIM7	1.09716E-05	3.17	5.16	1.63	0.86	0.73	0.98
cg00763151	12	SLC25A3	9.4199E-06	3.16	5.22	1.65	0.83	0.70	0.97
cg13284181	12	ZNF140	1.64447E-07	3.15	6.29	1.99	0.75	0.58	0.91
cg22626663	17	SPAG7	0.001345668	3.15	3.75	1.19	0.80	0.66	0.95
cg22830701	11	BDNFOS	1.16484E-05	3.15	5.17	1.64	0.77	0.62	0.93
cg16327227	2	DTNB	0.047861363	3.15	2.42	0.77	0.76	0.60	0.92
cg25743719	2	HPCAL1	9.77435E-39	3.14	13.24	4.22	0.77	0.62	0.93
cg03974185	12	UNG	9.30605E-06	3.13	5.25	1.68	0.85	0.71	0.98
cg14993543	7	FOXK1	2.55031E-07	3.13	6.20	1.98	0.75	0.58	0.91
cg09693243	9	ASTN2; TRIM32	0.000400153	3.13	4.16	1.33	0.82	0.68	0.96
cg11662678	2	DGUOK	7.2708E-09	3.13	7.10	2.27	0.79	0.64	0.94
cg19532257	2	KIF5C	1.0475E-11	3.11	8.66	2.78	0.84	0.70	0.97
cg15421984	5	RICTOR	1.29425E-07	3.11	6.41	2.06	0.79	0.64	0.94
cg07311313	13	CDX2	6.05819E-10	3.10	7.74	2.50	0.80	0.65	0.94
cg16645584	1	JUN	4.15333E-40	3.10	18.00	5.81	0.85	0.71	0.98
cg26080798	6	E2F3	3.26225E-39	3.09	15.01	4.86	0.88	0.76	0.99
cg18840956	5	PCSK1	3.47947E-15	3.08	10.55	3.42	0.82	0.69	0.96
cg03238298	18	DTNA	2.83095E-38	3.08	11.71	3.81	0.75	0.59	0.91
ch.18.658499F	18	RPRD1A	7.26851E-39	3.07	13.82	4.50	0.85	0.73	0.98
cg17303299	12	ENO2	2.85041E-06	3.05	5.67	1.86	0.81	0.67	0.96
cg20755690	1	MRPL55	0.006121793	3.05	3.30	1.08	0.81	0.66	0.95
cg18219532	7	DMTF1	3.78064E-38	3.05	11.30	3.71	0.79	0.64	0.94
cg14021564	10	PSD	4.82998E-07	3.04	6.18	2.03	0.84	0.71	0.97
cg24005309	10	IDI1	0.000742238	3.03	4.05	1.34	0.90	0.79	1.00
cg12968518	2	ACVR2A	2.33986E-39	3.02	15.66	5.18	0.85	0.72	0.98
cg27002185	11	CDKN1C	0.000138678	3.02	4.59	1.52	0.84	0.71	0.97
cg16998883	1	TAF13	0.001327733	3.01	3.87	1.28	0.81	0.66	0.95
cg11347840	17	SMARCE1	7.54536E-42	3.01	23.83	7.92	0.89	0.78	1.00
cg22129639	15	TP53BP1	2.31323E-05	3.01	5.14	1.71	0.79	0.64	0.94
cg09996344	7	KCTD7	2.20478E-40	3.00	19.20	6.40	0.85	0.71	0.98
cg09190219	12	CD63	2.94717E-38	3.00	11.80	3.94	0.78	0.62	0.93
cg16837525	21	DYRK1A	0.003308985	2.99	3.57	1.19	0.76	0.60	0.92
cg13688524	5	BOD1	2.09642E-05	2.98	5.20	1.74	0.76	0.60	0.92
cg25062542	1	ATP1A1	1.43417E-39	2.98	16.51	5.54	0.83	0.69	0.96
cg19349348	2	TCF7L1	0.026701409	2.98	2.76	0.93	0.81	0.66	0.95
cg22561749	1	RGS2	7.76522E-07	2.97	6.14	2.07	0.75	0.59	0.91
cg06547404	2	PARD3B	0.001602522	2.97	3.84	1.29	0.76	0.60	0.92
cg16882097	18	NPC1	0.00027128	2.97	4.43	1.49	0.83	0.70	0.97
cg08887356	15	PARP16	0.000686056	2.96	4.13	1.40	0.75	0.59	0.91
cg12940406	17	IKZF3	0.000314265	2.96	4.39	1.48	0.78	0.62	0.93
cg09276368	10	SLIT1	3.25554E-15	2.96	11.05	3.73	0.76	0.60	0.92
cg07448771	11	SUV420H1	0.000962508	2.94	4.04	1.37	0.83	0.69	0.96
cg06666025	16	CMTM2	2.24531E-07	2.92	6.58	2.26	0.84	0.71	0.97
cg02273875	9	ST6GALNAC4	3.49947E-05	2.91	5.14	1.77	0.90	0.79	1.00
cg07627390	16	PALB2; DCTN5	7.49425E-06	2.91	5.60	1.93	0.77	0.61	0.92
cg26976390	11	ZBTB16	1.19403E-38	2.90	13.44	4.63	0.83	0.70	0.97

cg22355463	4	ZNF827	4.80985E-42	2.90	24.89	8.58	0.87	0.75	0.99
cg15044280	9	NFIB	2.50795E-38	2.89	12.27	4.24	0.77	0.61	0.92
cg27159088	17	TEX14	5.18422E-12	2.88	9.39	3.26	0.84	0.70	0.97
ch.13.1783356F	13	COL4A2	1.36464E-39	2.88	16.88	5.86	0.78	0.63	0.93
cg11447942	9	PIP5KL1	6.27599E-06	2.86	5.72	2.00	0.88	0.76	0.99
cg12531974	9	PBX3	0.022411751	2.85	2.92	1.02	0.81	0.67	0.95
cg18739577	12	LTA4H	4.2215E-09	2.84	7.79	2.74	0.84	0.71	0.97
cg04090241	3	TBL1XR1	8.98172E-14	2.84	10.51	3.70	0.89	0.78	1.00
cg27496650	8	TOX	1.14258E-42	2.83	27.12	9.58	0.75	0.59	0.91
cg06529883	20	PLCB4	2.05608E-38	2.83	12.75	4.51	0.80	0.65	0.94
cg00207100	15	UBE2Q2P2; GOLGA6L10	9.74603E-12	2.82	9.41	3.33	0.86	0.74	0.99
cg10566589	6	TRAF3IP2	0.003564621	2.82	3.68	1.30	0.78	0.63	0.93
cg05184864	8	CPSF1	0.023253402	2.82	2.92	1.04	0.80	0.66	0.95
cg05086178	16	PDPK1	2.20443E-11	2.82	9.21	3.27	0.78	0.62	0.93
cg25189007	15	CCPG1	6.61493E-07	2.81	6.47	2.30	0.87	0.75	0.99
cg15825264	17	ELP2P; GEMIN4	6.4652E-05	2.81	5.09	1.81	0.87	0.75	0.99
ch.22.772318F	22	NAGA	3.46221E-38	2.80	11.95	4.26	0.93	0.84	1.00
cg11026382	1	PRKACB	3.98929E-39	2.80	15.46	5.52	0.79	0.64	0.94
cg05702851	4	CCDC109B	0.001102861	2.80	4.13	1.48	0.78	0.62	0.93
cg12567844	18	ANKRD29	1.04611E-09	2.79	8.28	2.96	0.75	0.58	0.91
cg11944735	9	ZCCHC6	2.74145E-38	2.79	12.36	4.42	0.81	0.67	0.96
cg10815272	5	PARP8	0.018453743	2.78	3.05	1.10	0.78	0.63	0.93
cg23649731	14	ATG2B; GSKIP	5.62849E-39	2.78	14.99	5.40	0.80	0.66	0.95
cg09949642	1	RCOR3	0.001124013	2.78	4.16	1.50	0.81	0.66	0.95
cg17016394	21	OLIG1	1.65208E-12	2.77	10.03	3.62	0.78	0.62	0.93
cg10576261	7	AVL9; LSM5	0.001863049	2.77	3.98	1.44	0.80	0.65	0.94
cg23037274	19	MAP2K7	0.006774099	2.77	3.48	1.26	0.79	0.63	0.94
cg13757989	19	ZNF235	2.24523E-05	2.77	5.48	1.98	0.79	0.64	0.94
cg22519756	1	CTBS	0.000555763	2.76	4.42	1.60	0.86	0.73	0.98
cg16434372	6	BRP44L	1.90304E-42	2.76	26.85	9.73	0.86	0.73	0.98
cg10327723	19	SAFB; SAFB2	0.004050392	2.75	3.70	1.34	0.75	0.59	0.91
cg16093752	17	RAC3	0.028493171	2.75	2.88	1.05	0.84	0.71	0.97
cg13779055	10	NRP1	6.36063E-39	2.75	14.87	5.41	0.81	0.67	0.95
cg27236875	2	ERBB4	3.37765E-39	2.75	15.90	5.78	0.83	0.70	0.97
cg07918776	15	SHC4; EID1	1.75384E-10	2.75	8.89	3.23	0.89	0.77	1.00
cg18874502	12	ZNF26	1.49931E-38	2.75	13.48	4.91	0.79	0.64	0.94
cg23967249	4	ELMOD2	0.046042888	2.75	2.66	0.97	0.81	0.67	0.95
cg20769275	16	SEZ6L2	2.47702E-14	2.74	11.20	4.08	0.84	0.71	0.97
cg20152630	19	MYPOP	0.001366599	2.74	4.12	1.50	0.77	0.62	0.93
cg15831769	10	ARID5B	2.40573E-38	2.74	12.71	4.64	0.82	0.68	0.96
cg11326485	3	CHMP2B	6.40314E-13	2.74	10.38	3.79	0.76	0.60	0.92
cg14160599	1	PDE4DIP	7.17438E-11	2.74	9.16	3.34	0.82	0.69	0.96
cg03906033	2	SPEG	3.64165E-13	2.73	10.56	3.86	0.82	0.69	0.96
cg22869660	8	YWHAZ	0.000738791	2.72	4.37	1.61	0.87	0.75	0.99
cg10513253	16	SOCS1	0.003256604	2.72	3.82	1.41	0.77	0.62	0.93
cg22616050	3	PVRL3	2.86679E-40	2.71	19.89	7.34	0.79	0.64	0.94
cg20964589	5	HSD17B4	2.07308E-10	2.71	8.97	3.31	0.83	0.69	0.96
cg08375775	4	DDX60	4.32116E-14	2.70	11.25	4.17	0.83	0.70	0.97
cg21348554	1	KCND3	8.13375E-05	2.70	5.17	1.92	0.86	0.74	0.99
cg11482571	20	TFAP2C	5.48872E-05	2.69	5.31	1.97	0.78	0.63	0.94
cg17733353	9	VAV2	3.04377E-05	2.69	5.51	2.05	0.78	0.63	0.94
cg19140085	12	LMO3	1.36312E-05	2.69	5.77	2.15	0.78	0.63	0.93
cg20237291	3	CCDC52	9.22378E-05	2.68	5.15	1.92	0.77	0.61	0.92

cg13429308	2	CNNM3	0.025423255	2.68	2.99	1.12	0.76	0.60	0.92
cg17088247	6	ZNF165	0.006565124	2.66	3.60	1.36	0.83	0.69	0.97
cg23769968	1	HLX	6.82399E-05	2.66	5.30	1.99	0.76	0.60	0.92
cg02115850	16	CDT1	0.004491838	2.65	3.77	1.42	0.75	0.58	0.91
ch.1.1254155F	1	MACF1	1.47475E-39	2.64	17.60	6.65	0.76	0.60	0.92
cg08694555	3	RAB43	7.35786E-08	2.64	7.48	2.83	0.82	0.68	0.96
cg23615065	1	SYDE2	5.84866E-40	2.63	19.11	7.25	0.81	0.66	0.95
cg24348442	4	SNHG8	0.010069642	2.63	3.45	1.31	0.75	0.59	0.91
cg00172371	9	PTRH1; TTC16	0.000285493	2.62	4.85	1.85	0.78	0.63	0.93
cg23268892	14	HSP90AA1	0.002160653	2.62	4.09	1.56	0.81	0.67	0.96
cg00637058	15	BNIP2	3.99301E-08	2.62	7.73	2.95	0.77	0.62	0.93
cg21882527	3	TOP2B	6.93253E-07	2.61	6.88	2.64	0.77	0.61	0.92
cg01370063	17	MPP2	0.002161828	2.60	4.12	1.58	0.77	0.61	0.92
cg11752103	21	DIP2A	0.00059118	2.60	4.63	1.78	0.83	0.69	0.96
cg11746480	17	SNF8	0.000463065	2.59	4.72	1.82	0.77	0.61	0.92
cg17591341	17	PSMD12	1.1176E-10	2.59	9.54	3.68	0.88	0.76	0.99
ch.20.707667F	20	RBM39	1.77006E-38	2.59	13.69	5.30	0.77	0.61	0.92
cg20187825	17	PYCR1	9.33315E-15	2.58	12.22	4.73	0.80	0.65	0.95
cg23429200	8	SMIM19; SLC20A2	3.10198E-39	2.58	16.65	6.45	0.82	0.69	0.96
cg22585957	12	NECAP1	1.13014E-38	2.58	14.48	5.61	0.90	0.80	1.00
cg17590938	2	ARID5A	4.43702E-05	2.57	5.60	2.17	0.81	0.67	0.95
cg03301945	19	ILF3	1.95055E-06	2.57	6.64	2.58	0.78	0.63	0.94
cg11996143	2	RND3	7.77051E-14	2.57	11.66	4.53	0.83	0.69	0.96
cg10800082	10	PDCD4	5.63001E-39	2.57	15.71	6.12	0.83	0.70	0.97
cg13594244	19	ZNF780B	0.000439054	2.57	4.78	1.86	0.76	0.60	0.92
cg12753450	11	SC5DL	0.004005422	2.56	3.92	1.53	0.77	0.62	0.93
cg25532409	19	FBXL12	2.4049E-08	2.56	8.07	3.15	0.77	0.62	0.93
cg19720077	14	SAV1	0.001406488	2.56	4.35	1.70	0.89	0.78	1.00
cg13849552	18	SETBP1	1.04888E-38	2.56	14.70	5.75	0.81	0.66	0.95
cg07106881	4	FGFRL1	1.00158E-09	2.55	9.04	3.54	0.82	0.68	0.96
cg25654619	2	TGOLN2	1.08694E-07	2.55	7.62	2.99	0.89	0.77	1.00
cg06498773	7	TAX1BP1	6.2248E-10	2.55	9.21	3.61	0.76	0.60	0.92
cg23508333	21	RUNX1	0.021675464	2.55	3.19	1.25	0.81	0.67	0.96
cg15492820	11	SYT7	1.72814E-06	2.55	6.75	2.65	0.78	0.63	0.94
cg18036710	19	DYRK1B	0.000351942	2.54	4.90	1.93	0.76	0.60	0.92
cg24742219	4	TMEM175; GAK	0.039142657	2.54	2.90	1.14	0.78	0.63	0.94
cg15162827	19	RPL28	0.000865379	2.54	4.56	1.80	0.79	0.64	0.94
cg16931825	20	TCEA2	1.3591E-38	2.54	14.31	5.63	0.88	0.76	0.99
cg00963305	16	MMP15	0.000646865	2.54	4.68	1.84	0.76	0.60	0.92
cg23627620	6	TBPL1	0.0006701	2.53	4.68	1.85	0.77	0.61	0.92
cg11680792	19	DLL3	0.000745898	2.52	4.66	1.85	0.79	0.64	0.94
cg26206910	14	RALGAPA1	0.021857494	2.52	3.21	1.27	0.79	0.64	0.94
cg24478660	14	RGS6	1.48926E-41	2.52	25.37	10.07	0.86	0.73	0.98
cg04737131	7	HOXA2	5.38964E-14	2.51	12.08	4.81	0.79	0.64	0.94
cg11019437	1	DUSP10	0.002777368	2.51	4.15	1.66	0.80	0.65	0.95
cg12243375	11	GUCY1A2	0.015456481	2.49	3.40	1.36	0.75	0.59	0.91
cg08313638	10	DHTKD1	1.44405E-05	2.49	6.17	2.47	0.81	0.66	0.95
cg15033181	2	TRIB2	7.75078E-39	2.49	15.46	6.20	0.79	0.64	0.94
cg07380405	12	LPCAT3	1.95892E-11	2.49	10.47	4.20	0.80	0.66	0.95
cg06965122	4	GALNT7	0.000123444	2.49	5.40	2.17	0.76	0.60	0.92
cg23309992	3	ZNF445	2.98969E-12	2.49	11.05	4.45	0.86	0.73	0.98
cg16419098	16	N4BP1	0.000934272	2.49	4.63	1.86	0.81	0.67	0.96
cg22385477	4	CCDC110	1.53261E-14	2.49	12.62	5.08	0.93	0.83	1.00
cg25922163	19	GGN	0.000256632	2.48	5.13	2.07	0.84	0.71	0.97

cg12511887	5	CSNK1A1	5.15294E-06	2.48	6.55	2.64	0.83	0.69	0.96
cg27065415	6	VARS	6.30016E-09	2.48	8.75	3.53	0.76	0.60	0.92
cg00074853	6	CDK19; AMD1	1.32662E-10	2.47	9.98	4.03	0.75	0.59	0.91
cg22876327	1	ADIPOR1	0.00024136	2.47	5.18	2.09	0.87	0.75	0.99
cg00134028	19	WIZ	2.7512E-12	2.46	11.22	4.56	0.82	0.68	0.96
cg05845236	13	PCDH9	0.006511948	2.46	3.85	1.56	0.81	0.67	0.95
cg03210589	16	DOK4	2.91849E-05	2.46	6.00	2.44	0.78	0.62	0.93
ch.2.4853641F	2	ILKAP	3.4295E-10	2.46	9.77	3.98	0.80	0.65	0.95
cg20815084	6	BAT4; CSNK2B	0.014901716	2.45	3.47	1.42	0.78	0.63	0.94
cg01292486	7	SRI	0.007874023	2.45	3.77	1.54	0.76	0.60	0.92
cg10038092	1	AK2	0.02952091	2.45	3.14	1.28	0.77	0.62	0.93
cg09428537	16	AFG3L1; CENPBD1	5.14025E-05	2.45	5.82	2.38	0.90	0.80	1.00
cg00908185	8	SQLE	1.44893E-06	2.44	7.10	2.91	0.76	0.60	0.92
ch.10.818442R	10	ARHGAP12	1.49112E-09	2.44	9.39	3.85	0.81	0.66	0.95
cg23753855	5	MEF2C	1.06997E-09	2.44	9.50	3.90	0.75	0.59	0.91
cg26393294	14	NPAS3	3.64596E-12	2.43	11.31	4.66	0.79	0.64	0.94
cg17780891	3	IP6K2	8.5575E-15	2.43	13.16	5.42	0.77	0.62	0.93
cg18023951	11	XRRA1; SPCS2	8.46295E-08	2.43	8.12	3.34	0.82	0.68	0.96
cg22942838	19	ZBTB7A	0.007849015	2.42	3.81	1.57	0.75	0.59	0.91
cg01906998	17	ACOX1	0.00137692	2.41	4.60	1.91	0.81	0.67	0.96
cg18567327	1	MKNK1	0.0196843	2.41	3.39	1.41	0.75	0.59	0.91
cg25547232	19	CNN2	0.006145397	2.41	3.95	1.64	0.76	0.60	0.92
cg03897712	7	ELN	2.04858E-08	2.40	8.70	3.62	0.80	0.66	0.95
cg00536383	2	ASAP2	0.013845933	2.40	3.58	1.49	0.78	0.63	0.94
ch.1.2289685F	1	BTBD8	3.27869E-13	2.39	12.27	5.13	0.81	0.67	0.96
cg22664697	19	ZNF554	0.042250912	2.39	3.02	1.27	0.87	0.75	0.99
cg08701551	17	ARSG; SLC16A6	0.002306212	2.38	4.43	1.86	0.81	0.66	0.95
cg15487992	19	UBL5	2.89783E-05	2.38	6.20	2.60	0.76	0.60	0.92
cg04691892	16	VPS4A	4.08429E-05	2.38	6.08	2.55	0.85	0.72	0.98
cg18165031	14	SLC39A9; ERH	0.007717975	2.38	3.89	1.63	0.84	0.70	0.97
cg24177604	4	ANTXR2	7.31636E-09	2.38	9.13	3.84	0.80	0.66	0.95
cg21635917	5	CAST	0.000966398	2.38	4.81	2.02	0.85	0.73	0.98
cg26617789	17	NTN1	0.002376156	2.38	4.43	1.86	0.87	0.75	0.99
cg00958884	17	CYGB	6.03851E-10	2.37	9.98	4.20	0.79	0.64	0.94
cg15932738	12	PTMS	0.001038005	2.37	4.80	2.03	0.91	0.81	1.00
cg18430990	1	TMEM240	1.28986E-40	2.37	23.02	9.71	0.78	0.63	0.93
cg26731327	8	BAI1	4.94672E-10	2.37	10.08	4.26	0.88	0.76	0.99
cg18251019	10	OBFC1	0.002944106	2.37	4.35	1.84	0.86	0.74	0.99
cg05129039	11	ZNHIT2	0.013414094	2.37	3.64	1.54	0.76	0.60	0.92
ch.15.1221715R	15	SIN3A	2.49794E-13	2.37	12.53	5.29	0.82	0.68	0.96
cg00536289	16	ATXN2L	0.018138072	2.36	3.50	1.48	0.76	0.60	0.92
ch.10.2734064R	10	BCCIP	5.31106E-10	2.36	10.10	4.28	0.75	0.59	0.91
cg02665463	17	ARL17A	2.15293E-40	2.36	22.26	9.44	0.75	0.59	0.91
cg23283236	19	PAK4	0.000830483	2.36	4.92	2.09	0.78	0.62	0.93
cg27607898	6	PECI	0.007291077	2.35	3.96	1.68	0.81	0.67	0.95
cg09366703	17	NME2; NME1	0.003005376	2.35	4.37	1.86	0.76	0.60	0.92
cg06983174	8	XKR4	3.74185E-43	2.34	32.26	13.77	0.81	0.66	0.95
cg01373262	20	DBNDD2; SYS1	2.70966E-09	2.34	9.65	4.12	0.77	0.62	0.93
cg09873429	6	RNU6-71P	1.0236E-38	2.34	15.67	6.69	0.80	0.66	0.95
ch.2.4958278F	2	PASK	5.53681E-14	2.34	13.17	5.63	0.87	0.75	0.99
cg06384694	12	RHNO1; FOXM1	6.88703E-09	2.34	9.35	4.00	0.80	0.65	0.94
cg21002970	11	MTA2	0.009560505	2.34	3.85	1.65	0.76	0.60	0.92
cg06316315	17	RHBDL3	4.38119E-05	2.33	6.18	2.65	0.85	0.73	0.98
cg15083233	9	PALM2; AKAP2	9.81154E-11	2.33	10.80	4.63	0.75	0.59	0.91

cg09221924	2	ADCY3	0.000458251	2.33	5.23	2.24	0.77	0.62	0.93
cg12339905	11	FEZ1	0.047411698	2.33	3.02	1.30	0.84	0.71	0.97
cg07827337	19	PAPL	0.003923581	2.33	4.29	1.84	0.78	0.63	0.93
cg20686479	11	NAV2	0.000417689	2.31	5.32	2.30	0.77	0.61	0.92
cg17615063	15	HCN4	0.000301155	2.31	5.46	2.36	0.75	0.58	0.91
cg13520931	21	ICOSLG	0.012857405	2.30	3.76	1.63	0.75	0.59	0.91
cg00984602	6	SRF	1.27122E-07	2.30	8.49	3.69	0.76	0.60	0.92
ch.17.1132347R	17	DHX8	2.72058E-13	2.30	12.98	5.64	0.80	0.65	0.94
cg21428647	3	HES1	3.30845E-39	2.30	17.93	7.80	0.75	0.59	0.91
cg07305215	18	ZFP161	0.01085384	2.30	3.85	1.68	0.83	0.69	0.97
cg19628745	6	MYLIP	5.25752E-07	2.29	8.01	3.49	0.76	0.60	0.92
cg17650050	22	HIRA; MRPL40	0.004235074	2.29	4.33	1.89	0.80	0.65	0.94
cg17235374	17	TAOK1	0.005580366	2.29	4.20	1.83	0.80	0.66	0.95
cg07814697	19	PLIN3	7.50183E-09	2.29	9.57	4.18	0.79	0.64	0.94
cg23139473	8	WRN; PURG	0.001796217	2.29	4.74	2.07	0.75	0.58	0.91
cg27301343	19	EML2	1.11331E-09	2.28	10.27	4.49	0.89	0.78	1.00
cg06971720	5	PRKAA1	0.0007692	2.28	5.13	2.25	0.77	0.62	0.93
cg22811818	3	SETD5	0.002545351	2.28	4.59	2.01	0.77	0.61	0.92
cg00384577	19	ITPKC; ADCK4	0.01913744	2.28	3.59	1.57	0.82	0.68	0.96
cg09916698	1	JMJD4; SNAP47	0.003107381	2.28	4.50	1.97	0.94	0.86	1.00
cg07010314	1	KIF21B	1.45034E-05	2.28	6.79	2.98	0.82	0.69	0.96
cg23659592	1	PEA15	0.006042472	2.27	4.20	1.85	0.77	0.62	0.93
ch.1.893309F	1	ARID1A	4.43629E-08	2.27	9.04	3.98	0.80	0.66	0.95
cg11838640	15	MAP2K5	2.8775E-40	2.27	22.44	9.89	0.84	0.70	0.97
cg17121541	19	DPF1	0.001350092	2.27	4.91	2.17	0.79	0.64	0.94
cg04861380	7	TMEM176B	6.98814E-06	2.27	7.13	3.15	0.77	0.62	0.93
cg05770546	10	ADK	2.32689E-41	2.26	26.76	11.86	0.78	0.63	0.93
cg18086592	12	HTR7P; HEBP1	0.006719055	2.26	4.17	1.85	0.81	0.66	0.95
cg06307169	17	CASC3	3.2043E-39	2.25	18.27	8.10	0.96	0.89	1.00
cg11472541	17	FDXR	0.000316866	2.25	5.60	2.49	0.82	0.68	0.96
cg13707780	11	DIXDC1	0.001333492	2.25	4.96	2.21	0.75	0.59	0.91
cg03227078	19	ZNF221	3.14785E-09	2.25	10.12	4.50	0.77	0.62	0.93
cg21763952	12	TCTN1	0.000145091	2.25	5.95	2.65	0.82	0.68	0.96
cg19362572	15	SHC4	4.26605E-14	2.24	14.04	6.26	0.79	0.64	0.94
cg13665940	22	TOP3B	0.011481843	2.24	3.92	1.75	0.86	0.74	0.99
cg02655864	17	RNMTL1; GLOD4	0.001807855	2.24	4.83	2.15	0.79	0.64	0.94
cg23094080	8	DGAT1	3.57616E-09	2.24	10.12	4.52	0.81	0.66	0.95
cg07460449	16	TSNAXIP1; RANBP10	6.06909E-39	2.24	17.20	7.69	0.95	0.88	1.00
cg02133183	10	FAM190B	0.007756453	2.23	4.14	1.85	0.75	0.59	0.91
cg02675375	4	MND1	0.003075303	2.23	4.61	2.07	0.75	0.58	0.91
cg12758922	20	RALGAPB	0.000183525	2.23	5.91	2.65	0.79	0.64	0.94
cg15290708	1	EFHD2	4.88927E-05	2.22	6.49	2.92	0.84	0.71	0.97
cg26215887	10	SLC25A28	2.06081E-07	2.22	8.69	3.91	0.81	0.67	0.96
cg03131395	17	STAT3	7.54003E-06	2.22	7.28	3.28	0.81	0.66	0.95
cg00518698	21	ITSN1; CRYZL1	0.000445674	2.22	5.54	2.50	0.80	0.66	0.95
cg19286308	11	SLC25A22; PANO1	0.001295784	2.22	5.05	2.28	0.79	0.64	0.94
cg13727849	17	MGC16275; TTYH2	6.51873E-05	2.22	6.39	2.88	0.75	0.59	0.91
cg02582682	3	RAB43; ISY1; RAB43	1.65709E-08	2.21	9.72	4.40	0.79	0.64	0.94
cg08789220	11	RTN4RL2	0.045553261	2.21	3.21	1.45	0.79	0.64	0.94
cg25479742	4	FAM198B	2.00133E-39	2.21	19.43	8.79	0.83	0.69	0.97
cg02478448	13	CCNA1	1.37979E-40	2.21	24.20	10.95	0.82	0.69	0.96
cg21850069	5	TRIM41	1.5294E-14	2.21	14.77	6.69	0.75	0.58	0.91
cg19344263	19	ZNF584	2.95975E-14	2.21	14.53	6.58	0.81	0.67	0.96
cg16526137	16	ZFP90	1.94037E-39	2.21	19.51	8.84	0.80	0.65	0.94

cg13404054	19	NOTCH3	6.86643E-08	2.20	9.24	4.20	0.80	0.65	0.95
cg19595402	17	SRCIN1	0.004816139	2.20	4.47	2.03	0.82	0.68	0.96
ch.9.2473665R	9	PMPCA	4.52705E-15	2.19	15.37	7.01	0.83	0.70	0.97
cg10507402	2	DPP4	0.004331177	2.19	4.53	2.07	0.76	0.60	0.92
cg11270070	3	ZMYND10	0.001380937	2.19	5.10	2.33	0.77	0.61	0.92
cg07355392	6	SEC63	0.00087272	2.19	5.32	2.43	0.83	0.69	0.96
cg09412014	20	IFT52	0.012690268	2.18	3.98	1.82	0.78	0.62	0.93
cg16188589	3	IRAK2	4.43335E-10	2.18	11.27	5.16	0.79	0.64	0.94
cg14904433	1	NUCKS1	1.34075E-06	2.18	8.15	3.73	0.82	0.67	0.96
cg17282161	20	SDC4	0.018969528	2.18	3.76	1.72	0.81	0.67	0.96
cg03411765	8	TSNARE1	2.93555E-40	2.18	23.12	10.60	0.81	0.66	0.95
cg03382048	1	PLEKHM2	0.0034492	2.18	4.67	2.14	0.77	0.61	0.92
cg21785522	10	GFRA1	1.90879E-39	2.18	19.75	9.07	0.80	0.66	0.95
cg07600127	13	KATNAL1	0.004425283	2.18	4.55	2.09	0.77	0.61	0.92
cg18708365	7	ORC5L	1.18147E-09	2.18	10.96	5.04	0.75	0.59	0.91
ch.7.246556R	7	RNF216	1.03627E-39	2.17	20.91	9.62	0.79	0.64	0.94
cg15336627	9	CDC14B	8.92445E-09	2.17	10.25	4.73	0.84	0.71	0.97
cg24050671	11	CCDC15	1.53291E-10	2.16	11.83	5.47	0.78	0.63	0.94
cg24040576	1	SMYD3	0.042941769	2.16	3.32	1.54	0.79	0.64	0.94
cg08881202	14	CCNB1IP1	2.41181E-09	2.16	10.81	5.01	0.77	0.61	0.92
cg09615786	3	DCBLD2	0.006376765	2.16	4.41	2.04	0.78	0.62	0.93
cg19118262	16	COG8; PDF	0.006650472	2.16	4.39	2.03	0.81	0.67	0.96
cg08681365	8	SPAG1	1.15961E-41	2.15	29.00	13.48	0.87	0.75	0.99
cg25635304	16	FAM65A	4.24629E-39	2.15	18.46	8.59	0.79	0.64	0.94
ch.11.966965R	11	CKAP5	1.96104E-08	2.15	10.04	4.67	0.76	0.60	0.92
cg11078175	7	ACTR3B	0.002220309	2.15	4.98	2.32	0.78	0.63	0.93
cg00179014	9	RALGPS1	0.002404786	2.15	4.94	2.30	0.91	0.80	1.00
ch.20.221631R	20	ANKRD5	1.10744E-11	2.15	12.96	6.03	0.82	0.68	0.96
cg13173564	19	MPND	3.61138E-07	2.15	8.88	4.14	0.81	0.66	0.95
cg26838212	10	BMS1	0.007461115	2.14	4.37	2.04	0.78	0.62	0.93
cg16570157	11	EFCAB4A	0.003307074	2.14	4.81	2.25	0.75	0.58	0.91
cg25518707	17	MARCH10;	1.01427E-10	2.14	12.21	5.72	0.78	0.63	0.94
cg25890275	11	VPS37C	0.006715621	2.13	4.44	2.08	0.76	0.60	0.92
cg24188415	10	EMX2	0.014312987	2.13	4.03	1.89	0.86	0.73	0.98
cg16123774	13	NEK3	0.010522312	2.13	4.20	1.97	0.77	0.61	0.92
cg21816532	17	HOXB6	2.20728E-08	2.13	10.15	4.77	0.89	0.78	1.00
cg24737639	12	NUP37	0.022361072	2.12	3.78	1.78	0.76	0.60	0.92
cg11251319	19	ATP8B3	1.30096E-07	2.12	9.45	4.45	0.76	0.60	0.92
cg03328060	17	ZNF624	0.040104892	2.12	3.44	1.62	0.76	0.60	0.92
cg27204163	3	EIF2B5	5.39595E-06	2.12	7.88	3.72	0.76	0.60	0.92
cg14446015	8	ZNF395	0.034445518	2.12	3.54	1.67	0.79	0.63	0.94
cg12070221	10	ANK3	4.34969E-10	2.12	11.82	5.58	0.78	0.62	0.93
cg06022942	10	FLJ45983; GATA3	1.04917E-05	2.12	7.61	3.59	0.76	0.60	0.92
cg15972331	13	FGF14	7.58769E-09	2.12	10.67	5.05	0.79	0.64	0.94
ch.22.363412F	22	AP1B1	8.9185E-15	2.11	16.06	7.61	0.81	0.66	0.95
ch.19.332523F	19	GTF2F1	5.99105E-09	2.11	10.82	5.13	0.75	0.59	0.91
cg02720697	19	NFIC	6.22587E-39	2.11	18.04	8.56	0.87	0.75	0.99
cg12784766	17	CYTH1	0.029587775	2.11	3.65	1.74	0.78	0.62	0.93
ch.17.1364760R	17	CA10	8.12394E-07	2.11	8.79	4.17	0.75	0.59	0.91
cg16256643	19	NDUFB7	6.39116E-15	2.10	16.32	7.76	0.81	0.67	0.95
cg08101646	18	SLC14A2	6.89702E-06	2.10	7.86	3.74	0.83	0.69	0.97
cg16803522	15	RASGRP1	0.043309519	2.10	3.42	1.63	0.81	0.66	0.95
cg07001901	16	PLCG2	1.02886E-08	2.10	10.66	5.07	0.82	0.68	0.96
cg11146173	14	MTA1	0.017187648	2.10	3.99	1.90	0.75	0.58	0.91

cg09781054	20	ZSWIM1	1.10315E-07	2.10	9.69	4.62	0.84	0.70	0.97
cg15321195	21	AGPAT3	1.56039E-09	2.10	11.47	5.47	0.76	0.60	0.92
cg05157140	7	UNCX	7.44484E-05	2.10	6.81	3.25	0.79	0.64	0.94
cg22800477	1	CASZ1	1.67579E-39	2.10	20.69	9.88	0.84	0.70	0.97
cg15926590	6	PSMB9; TAP1	0.003337545	2.09	4.92	2.35	0.82	0.68	0.96
cg05175333	18	ROCK1	0.009285024	2.09	4.37	2.09	0.75	0.59	0.91
cg14159220	17	SEC14L1	4.90079E-08	2.09	10.09	4.83	0.78	0.62	0.93
cg20719524	15	RPP25	9.71672E-39	2.08	17.35	8.32	0.84	0.70	0.97
cg03556069	2	TMEM163	0.006211594	2.08	4.61	2.21	0.76	0.60	0.92
cg10840007	14	CDCA4	0.000299738	2.08	6.22	3.00	0.75	0.59	0.91
cg16546279	17	ATXN7L3	4.01087E-08	2.07	10.31	4.97	0.75	0.59	0.91
cg18548208	14	EML5	4.41085E-06	2.07	8.23	3.97	0.79	0.64	0.94
cg11927881	11	ELMOD1	1.0678E-12	2.07	14.73	7.12	0.92	0.82	1.00
cg19983274	19	LPHN1	0.002520816	2.07	5.16	2.50	0.84	0.71	0.97
cg14496450	17	HRNBP3	0.006006844	2.05	4.72	2.30	0.76	0.60	0.92
cg09418984	19	MYADM	4.37873E-05	2.05	7.29	3.56	0.81	0.67	0.95
cg08723176	12	PXN	0.011706612	2.05	4.35	2.12	0.77	0.61	0.92
cg20755163	1	KIAA2013	0.005270969	2.05	4.82	2.35	0.76	0.60	0.92
cg13703070	9	NR4A3	5.77783E-06	2.04	8.28	4.05	0.81	0.66	0.95
cg21533369	22	SFI1	0.042232572	2.04	3.56	1.74	0.82	0.68	0.96
cg07860213	8	PRDM14	0.026015058	2.04	3.88	1.90	0.79	0.64	0.94
cg27234067	11	WIT1	0.001151327	2.04	5.67	2.78	0.78	0.62	0.93
cg17030173	5	MARCH11	4.93414E-42	2.03	32.05	15.77	0.90	0.80	1.00
cg23118464	2	AFF3	0.038656325	2.03	3.64	1.79	0.80	0.65	0.94
cg13219446	1	DCAF8	2.69496E-06	2.03	8.73	4.30	0.76	0.60	0.92
cg10175688	17	TXNDC17; KIAA0753	0.000150357	2.03	6.78	3.34	0.80	0.65	0.95
cg23690809	1	PPP1R12B	4.88534E-11	2.03	13.60	6.70	0.88	0.77	1.00
cg19564098	10	HMX3	0.042516073	2.03	3.59	1.77	0.75	0.58	0.91
cg05279413	7	MAFK	2.94847E-09	2.02	11.89	5.88	0.78	0.62	0.93
cg11097433	10	SORCS1	0.000777344	2.02	5.97	2.96	0.75	0.59	0.91
cg06463759	7	ATP6V1F	2.15759E-39	2.02	20.94	10.38	0.81	0.66	0.95
cg21392576	2	NFE2L2	1.92969E-14	2.01	17.08	8.48	0.76	0.60	0.92
cg04588455	16	MAZ	0.010956968	2.01	4.49	2.23	0.75	0.58	0.91
cg18221121	10	WDR37	2.12167E-06	2.01	8.98	4.47	0.75	0.59	0.91
cg26933691	19	COX6B1	0.001201431	2.01	5.79	2.88	0.86	0.73	0.98
cg09806934	11	CPT1A	1.40184E-05	2.00	8.10	4.04	0.87	0.75	0.99
cg02009141	1	UBE2T	3.24733E-05	2.00	7.69	3.84	0.75	0.59	0.91
cg13583085	5	UBE2B	5.44323E-07	2.00	9.69	4.84	0.79	0.64	0.94
cg24606935	20	HCK	1.68923E-10	4.62	6.44	1.39	0.76	0.60	0.92
cg09390251	17	RABEP1	0.001730446	4.21	3.19	0.76	0.76	0.60	0.92
cg20337934	2	SNRNP27	2.16937E-07	3.81	5.56	1.46	0.75	0.59	0.91
cg11081723	20	PANK2	0.001245853	3.50	3.57	1.02	0.75	0.58	0.91
cg12035585	17	FAM134C; TUBG1	0.02725959	3.03	2.73	0.90	0.80	0.65	0.94
cg01157809	2	AGBL5	0.011036615	2.83	3.23	1.14	0.75	0.59	0.91
cg24984691	1	POLR3C; RNF115; NBPF20; NBPF10	0.022315865	2.57	3.15	1.23	0.81	0.66	0.95
cg01045337	16	AXIN1	4.08703E-05	2.53	5.71	2.26	0.78	0.63	0.94
cg03212129	19	PDCD2L	0.045681852	2.30	3.08	1.34	0.90	0.79	1.00
cg23669159	1	PLEKHO1	9.57119E-10	2.26	10.49	4.65	0.81	0.66	0.95
cg09936907	12	USP5; CDCA3	0.037224524	2.01	3.72	1.85	0.81	0.67	0.95
cg00949022	13	PIBF1; DIS3	0.000701884	2.16	5.50	2.54	0.76	0.60	0.92
cg05789608	1	DSTYK	0.001976424	3.13	3.65	1.17	0.77	0.61	0.92
cg07130520	1	DDAH1	0.006651328	2.19	4.31	1.97	0.84	0.70	0.97
cg03369465	6	DAXX	2.37687E-08	3.01	7.00	2.33	0.80	0.66	0.95

cg19565306	22	RPL23AP82; RABL2B	0.000488579	2.48	4.89	1.97	0.76	0.60	0.92
cg05898482	7	XRCC2	2.74809E-44	2.41	35.28	14.67	0.87	0.75	0.99
cg13620370	5	PDLIM7	0.016110285	2.39	3.51	1.47	0.75	0.59	0.91
cg20552696	5	MED7	0.027091568	2.43	3.21	1.32	0.77	0.61	0.92
cg13432900	19	RPS11	0.001557325	2.60	4.25	1.64	0.79	0.64	0.94
cg19823793	3	SGOL1	1.17112E-12	3.76	8.08	2.15	0.81	0.66	0.95
cg08427575	7	YWHAG	1.21022E-12	4.19	7.62	1.82	0.81	0.67	0.95
cg07737564	19	ZNF223	0.042669131	2.31	3.10	1.34	0.76	0.60	0.92
cg24333469	22	SLC7A4	5.61729E-06	2.76	5.92	2.14	0.84	0.71	0.97
cg03909712	6	AKD1; FIG4	6.95393E-06	2.05	8.13	3.96	0.80	0.65	0.94
cg09997463	12	CNOT2	3.04518E-15	2.76	11.76	4.26	0.86	0.73	0.98
cg17500645	3	LZTFL1	0.009249343	2.58	3.54	1.37	0.76	0.60	0.92
cg23209098	14	CYP46A1	2.69122E-38	3.72	10.88	2.93	0.82	0.68	0.96
cg03469617	2	MRPL53	4.45198E-06	3.92	4.81	1.23	0.83	0.69	0.96
cg16781205	14	DIO3	1.15541E-11	3.71	7.69	2.07	0.77	0.61	0.92
cg21861662	9	FANCC	0.001838807	3.10	3.69	1.19	0.80	0.65	0.95
cg06077359	9	DNAJC25; GNG10	0.018660112	2.92	2.95	1.01	0.79	0.64	0.94
cg09484638	19	DNASE2	0.000288285	2.88	4.51	1.57	0.80	0.65	0.95
cg20525831	1	ANKRD35; NBPF20	0.011957887	2.67	3.34	1.25	0.78	0.63	0.93
cg25979598	1	RTCD1	2.62911E-41	2.60	24.01	9.23	0.85	0.71	0.98
cg02573825	19	ZNF350	2.22266E-10	2.43	10.04	4.14	0.76	0.60	0.92
cg13359134	18	RTTN	0.006872013	2.39	3.92	1.64	0.75	0.59	0.91
cg16750717	1	SSX2IP	0.006431831	2.29	4.13	1.80	0.76	0.60	0.92
cg19706449	1	ATPAF1	1.07349E-07	2.26	8.73	3.85	0.82	0.68	0.96
cg13109300	12	DHH	0.000127221	2.26	5.98	2.65	0.81	0.66	0.95
cg10014408	9	SDCCAG3; PMPCA	0.044983242	2.10	3.40	1.62	0.88	0.76	0.99
cg05853013	6	VARS2	0.010038354	2.01	4.54	2.26	0.80	0.65	0.95
cg25674826	21	MRPS6; SLC5A3	7.12342E-10	2.07	12.03	5.81	0.85	0.72	0.98
cg21270074	20	BCAS4	1.05974E-09	2.61	8.83	3.38	0.78	0.63	0.94
cg10255675	14	HNRNPC	2.89993E-07	2.50	7.47	2.99	0.76	0.60	0.92
cg17074213	1	TGFBR3	3.47752E-43	2.15	34.62	16.07	0.83	0.69	0.96
cg09367529	20	TMEM230	0.025103638	2.76	2.94	1.07	0.88	0.76	0.99
cg03212862	20	CSE1L	0.007470744	2.31	4.02	1.74	0.80	0.65	0.94
cg08188860	9	PPP6C	1.88837E-12	4.17	7.56	1.82	0.76	0.60	0.92
cg19597529	3	PARP14	4.53003E-39	2.09	18.81	9.00	0.75	0.59	0.91
cg18070621	17	PCYT2	0.000962465	2.85	4.13	1.45	0.88	0.76	1.00
cg12315363	17	TAF15	2.43805E-06	3.16	5.59	1.77	0.78	0.62	0.93
cg00045303	21	SETD4	0.005483618	2.39	4.04	1.69	0.78	0.63	0.93
cg18412834	20	FLJ16779; NKAIN4	0.030208238	2.35	3.24	1.38	0.79	0.64	0.94
cg09724301	2	GAD1	0.000108687	2.18	6.31	2.90	0.81	0.67	0.96
cg00440776	15	SPRED1	0.009362296	4.40	2.64	0.60	0.76	0.60	0.92
cg04842828	21	IFNAR1	3.53897E-11	4.40	6.86	1.56	0.83	0.70	0.97
cg16399182	6	COQ3	1.11733E-37	5.41	7.87	1.45	0.90	0.80	1.00
cg18555073	15	B2M	1.39767E-06	2.10	8.56	4.07	0.77	0.61	0.92
cg07212963	1	HMGCL	3.39862E-14	4.62	7.88	1.70	0.79	0.64	0.94
cg25544073	8	MTDH	5.01675E-38	3.61	10.07	2.79	0.78	0.63	0.94
cg05134500	3	MFI2	2.51962E-38	3.33	11.47	3.45	0.85	0.71	0.98
cg26463106	3	THUMPD3	3.51056E-05	3.27	4.75	1.45	0.81	0.66	0.95
cg24454219	4	ZNF721; PIGG	3.28244E-06	3.10	5.58	1.80	0.85	0.72	0.98
cg08052226	19	NFIX	1.06633E-14	2.92	10.81	3.71	0.78	0.63	0.93
cg13370420	19	DACT3	0.000945816	2.82	4.16	1.48	0.77	0.62	0.93
cg26791126	1	DNAJC8	0.000690045	2.57	4.61	1.79	0.87	0.75	0.99
cg00505936	16	HAS3	1.33689E-09	2.41	9.54	3.95	0.79	0.64	0.94
cg21702506	12	SLC6A15	3.73513E-41	2.18	26.73	12.27	0.86	0.74	0.99

cg13607699	17	UBTF	1.18121E-38	2.17	16.37	7.55	0.75	0.59	0.91
cg24397040	21	PDXK	0.003895245	2.17	4.65	2.14	0.86	0.74	0.99
cg10754596	22	OSBP2	0.007695377	2.08	4.51	2.17	0.81	0.66	0.95
cg12876333	18	NEDD4L	0.001415955	2.07	5.46	2.64	0.79	0.64	0.94
cg22501899	17	AARSD1; RUNDC1	0.043229275	2.00	3.63	1.81	0.75	0.58	0.91
cg25026401	12	SLC15A4	1.23732E-40	2.26	23.97	10.62	0.82	0.69	0.96
cg19464917	15	ISL2	7.34034E-43	2.92	27.28	9.36	0.82	0.69	0.96
cg16880783	19	TRMT1	2.02122E-42	2.12	32.26	15.20	0.84	0.71	0.97
cg05205152	19	AKAP8L	0.009049844	2.92	3.24	1.11	0.91	0.81	1.00
cg14077010	11	NARS2	0.000865351	2.79	4.23	1.51	0.78	0.63	0.94
cg23235362	15	TSPAN3	2.31859E-05	3.74	4.52	1.21	0.75	0.59	0.91
cg18269503	6	HIST1H4C	7.87273E-07	2.91	6.24	2.15	0.77	0.62	0.93
cg23672480	12	APAF1; IKBIP	1.50141E-07	2.43	7.91	3.25	0.79	0.64	0.94
cg22576055	19	MORG1; MAN2B1	1.82163E-38	4.35	10.87	2.50	0.76	0.60	0.92
cg09881384	11	USP28	1.42228E-38	4.06	11.45	2.82	0.85	0.72	0.98
cg05856122	11	TAF10	4.5311E-15	4.68	8.12	1.74	0.82	0.68	0.96
cg27586068	19	DOHH	0.000875871	2.80	4.21	1.50	0.85	0.73	0.98
cg00807892	4	MOBKL1A	2.77503E-05	2.18	6.88	3.15	0.76	0.60	0.92
cg06995575	2	DPY30	0.01997031	2.09	3.93	1.88	0.88	0.76	0.99
cg09162102	12	TMPO	2.03525E-12	2.35	11.98	5.10	0.79	0.64	0.94
cg02417622	15	NUSAP1; OIP5	0.005079486	2.05	4.82	2.35	0.81	0.66	0.95
cg16793043	3	SNRK	2.0503E-39	4.08	14.06	3.45	0.84	0.71	0.97
cg18464001	18	KDSR	2.99505E-07	2.95	6.45	2.19	0.81	0.66	0.95
cg22517622	3	SGOL1	5.15099E-13	4.81	7.31	1.52	0.78	0.63	0.93
cg19478426	16	KLHDC4	4.74712E-39	4.05	12.97	3.20	0.82	0.69	0.96
cg07596209	1	PRDM2	0.001640107	2.84	3.95	1.39	0.81	0.66	0.95
cg27400713	9	CHMP5; BAG1	0.001060275	2.16	5.30	2.45	0.87	0.75	0.99
cg23056966	15	HDDC3; UNC45A	0.000153801	2.07	6.57	3.17	0.78	0.63	0.93
cg08484665	2	PCBP1	2.16709E-09	3.63	6.71	1.85	0.79	0.64	0.94
cg08535148	4	RBPJ	0.008342308	2.24	4.09	1.83	0.76	0.60	0.92
cg05608389	1	PDE4B	7.32323E-11	2.20	11.83	5.38	0.77	0.61	0.92
cg05349922	6	RWDD1	4.63776E-09	3.61	6.57	1.82	0.77	0.61	0.92
cg13126121	8	FLJ10661	0.044079108	2.10	3.41	1.62	0.76	0.60	0.92
cg14661464	2	TXNDC9; EIF5B	0.024215066	3.94	2.43	0.62	0.85	0.73	0.98
cg05775895	3	CAND2	0.003243623	2.80	3.74	1.34	0.79	0.64	0.94
cg21542780	2	HJURP	3.27212E-08	2.43	8.43	3.47	0.85	0.72	0.98
cg06383088	9	XPA	0.004984235	3.14	3.32	1.06	0.79	0.63	0.94
cg17054969	12	COL2A1	0.032613588	2.38	3.17	1.33	0.79	0.64	0.94
cg10929629	13	RNF219	7.13238E-14	3.54	8.94	2.53	0.79	0.64	0.94
cg03467424	10	EPC1	0.026469576	3.09	2.70	0.87	0.80	0.66	0.95
cg26485563	12	ANKRD52	6.66328E-38	5.57	8.51	1.53	0.75	0.59	0.91
cg10476512	19	IGLON5	7.13608E-11	3.33	7.83	2.35	0.76	0.60	0.92
cg21777986	18	BCL2	9.19628E-08	2.42	8.10	3.34	0.89	0.78	1.00
cg03056863	14	RABGGTA	0.000139608	2.35	5.66	2.41	0.75	0.58	0.91
cg03761649	12	RIMKLB	0.008604579	2.27	4.03	1.78	0.78	0.62	0.93
cg01576519	19	ILVBL	1.6816E-39	2.22	19.70	8.89	0.76	0.60	0.92
cg14002175	1	BCL9	5.0811E-09	2.10	10.93	5.19	0.82	0.68	0.96
cg08440521	4	RNF150	1.30209E-05	2.03	7.97	3.92	0.80	0.66	0.95
cg24111008	19	PNPLA6	5.52678E-05	2.87	5.05	1.76	0.83	0.69	0.96
cg01416537	4	NDST3	3.82677E-12	2.53	10.76	4.25	0.82	0.69	0.96
cg20146868	19	GRWD1	7.41274E-13	2.44	11.72	4.80	0.81	0.66	0.95
cg23598562	12	SP1	8.73255E-07	2.28	7.88	3.46	0.75	0.58	0.91
cg07840972	7	TMED4	3.30463E-05	3.94	4.32	1.10	0.81	0.67	0.96
cg25028996	14	PRMT5	0.013504372	2.15	4.02	1.87	0.75	0.59	0.91

cg05895714	17	RPL23	5.80792E-38	3.89	9.59	2.46	0.88	0.76	0.99
cg08884368	17	NME2; NME1	0.014016004	2.49	3.45	1.39	0.81	0.67	0.96
cg12497026	1	HSPB11; LRRC42	4.61387E-07	2.54	7.21	2.84	0.80	0.65	0.95
cg01943657	4	PPP3CA	0.049288743	2.08	3.38	1.63	0.86	0.73	0.98
cg11387577	6	SHPRH	6.68154E-38	3.99	9.31	2.34	0.78	0.63	0.93
cg13832679	9	CTNNAL1	2.6682E-38	3.19	11.61	3.64	0.80	0.65	0.95
cg07235453	8	TAF2	1.22613E-12	3.67	8.18	2.23	0.78	0.63	0.94
cg00609363	6	ZNRD1	3.83507E-38	7.95	8.67	1.09	0.75	0.59	0.91
cg14207210	17	PPM1E	5.07356E-05	2.40	5.95	2.48	0.88	0.77	1.00
cg05202407	17	TOP3A; SMCR8	1.03628E-05	2.57	6.09	2.37	0.75	0.59	0.91
cg15403346	6	TAPBP; RGL2	1.83127E-05	3.35	4.86	1.45	0.85	0.73	0.98
cg08886844	19	CD320	0.00110308	2.07	5.59	2.70	0.76	0.60	0.92
cg07296918	1	B4GALT2	0.010882723	2.81	3.25	1.16	0.83	0.69	0.97
cg17717362	16	HSDL1; DNAAF1	0.000479313	3.60	3.79	1.05	0.85	0.71	0.98
cg02117197	16	CENPT; NUTF2	9.83045E-08	3.42	6.09	1.78	0.81	0.66	0.95
cg04099673	18	WDR7	1.19634E-08	3.13	6.97	2.23	0.79	0.64	0.94
cg00905725	1	DFFB; KIAA0562	0.005086477	3.12	3.32	1.06	0.75	0.58	0.91
cg02480104	20	RPN2	0.001385512	2.97	3.89	1.31	0.78	0.63	0.94
cg15196314	19	CTU1	2.05218E-06	2.94	5.92	2.01	0.76	0.60	0.92
cg16930275	19	TRIP10	0.000101769	2.68	5.13	1.91	0.80	0.65	0.95
cg05003157	1	ZBTB41; CRB1	2.07652E-40	2.53	21.24	8.38	0.75	0.59	0.91
cg02824295	19	TBC1D17; AKT1S1	0.001498652	2.45	4.49	1.83	0.78	0.62	0.93
cg22931738	3	GATA2	4.9351E-12	2.41	11.33	4.71	0.77	0.61	0.92
cg07329149	2	NDUFB3; FAM126B	1.18304E-05	2.14	7.42	3.46	0.83	0.69	0.96
cg07707379	16	CYB5B	5.92821E-10	2.01	12.76	6.36	0.79	0.64	0.94
cg07811750	17	CAMTA2	0.001152391	2.71	4.22	1.56	0.75	0.59	0.91
cg27265032	16	CP110	1.51789E-09	2.07	11.76	5.69	0.85	0.72	0.98
cg03537301	5	MIER3	1.64896E-38	3.27	12.21	3.73	0.76	0.60	0.92
cg14439185	2	SGOL2	0.00093817	3.13	3.89	1.24	0.77	0.62	0.93
cg05294095	19	PSMD8	8.07704E-08	2.19	9.25	4.23	0.94	0.86	1.00
cg25344180	17	RNFT1	1.48259E-08	3.00	7.14	2.38	0.84	0.71	0.97
cg10602468	19	CYTH2	5.91176E-12	2.68	10.03	3.75	0.84	0.70	0.97
cg08336315	6	TRERF1	0.008181715	2.07	4.50	2.18	0.81	0.66	0.95
cg08897132	17	DCXR	4.30203E-06	2.44	6.74	2.76	0.79	0.65	0.94
cg18530950	2	IWS1	0.014482174	3.01	2.99	0.99	0.79	0.64	0.94
cg22267597	17	MYH10	0.00194373	3.63	3.36	0.93	0.81	0.66	0.95
cg00200289	17	GRB2	0.03432636	2.26	3.30	1.46	0.77	0.62	0.93
cg12229767	7	STAG3L1; STAG3L3; PMS2P7; PMS2P5; PMS2P2	3.37361E-40	4.71	15.73	3.34	0.82	0.68	0.96
cg08680689	19	ELOF1	9.80701E-05	2.88	4.86	1.69	0.82	0.68	0.96
cg02774380	1	AP4B1; DCLRE1B	6.92647E-05	2.28	6.16	2.71	0.75	0.59	0.91
cg01560790	2	PPP4R3B	0.000105084	3.09	4.61	1.49	0.78	0.63	0.93
cg21538020	3	SLC33A1	3.22517E-39	7.21	11.79	1.64	0.76	0.60	0.92
cg22963915	19	MATK	1.26896E-10	3.06	8.20	2.68	0.85	0.72	0.98
cg17720231	1	IGSF9	6.74508E-40	2.58	19.15	7.43	0.76	0.60	0.92
cg11939631	5	RUFY1	0.000655018	2.33	5.07	2.17	0.76	0.60	0.92
cg10646633	19	ZNF224	1.53883E-40	7.37	15.20	2.06	0.75	0.58	0.91
cg24139898	19	HPN	2.65129E-39	4.88	13.02	2.67	0.75	0.59	0.91
cg01892655	3	TMEM41A	9.48912E-41	4.22	17.81	4.22	0.83	0.69	0.97
cg12292808	19	UPF1	0.000430145	3.08	4.18	1.36	0.80	0.66	0.95
cg23948071	20	CENPB	2.54381E-05	2.87	5.29	1.85	0.79	0.64	0.94
cg11078683	17	LHX1	4.49182E-10	2.57	9.24	3.60	0.77	0.62	0.93
cg03281368	1	H3F3A; H3F3AP4	5.82074E-09	2.54	8.56	3.37	0.81	0.66	0.95

cg13597632	5	HMGCS1	3.37571E-06	2.38	6.99	2.93	0.83	0.69	0.97
cg05796727	21	JAM2	0.000940526	2.35	4.88	2.07	0.87	0.75	0.99
cg21015791	1	MDM4	0.016824577	2.17	3.86	1.78	0.76	0.60	0.92
cg06539804	20	CPXM1	1.67272E-09	2.17	10.90	5.03	0.75	0.59	0.91
cg11987819	21	ADARB1	0.00150805	2.14	5.18	2.42	0.80	0.65	0.94
cg22090640	19	CD3EAP; PPP1R13L	6.00809E-40	2.78	18.47	6.64	0.78	0.63	0.94
cg16444198	3	HLTF	3.81516E-08	2.05	10.52	5.14	0.81	0.66	0.95
cg21512185	2	GEMIN6	0.012368045	2.60	3.39	1.31	0.90	0.79	1.00
cg17625506	6	HLA-F	3.9682E-05	2.50	5.79	2.31	0.81	0.67	0.95
cg03003009	4	MED28	1.7157E-05	2.14	7.29	3.41	0.76	0.60	0.92
cg16219302	15	ZNF774	1.98362E-06	2.22	7.81	3.52	0.87	0.75	0.99
cg01381678	2	HECW2	0.003189563	2.06	5.04	2.44	0.81	0.67	0.95
cg17588917	3	ARPC4; TTLL3; TADA3	0.019602879	2.27	3.59	1.58	0.80	0.65	0.94
cg13471114	2	OTX1	1.09156E-14	2.33	13.77	5.90	0.76	0.60	0.92
cg05586242	3	BTD; HACL1	0.000583589	3.18	4.00	1.26	0.83	0.70	0.97
cg04826071	15	TTBK2	3.89522E-42	2.31	29.09	12.60	0.85	0.71	0.98
cg17211192	8	SNX16	4.08051E-07	2.24	8.36	3.74	0.77	0.62	0.93
cg20705251	17	ZBTB4	1.14283E-07	2.04	10.09	4.94	0.77	0.61	0.92
cg23831542	17	TMEM11	0.000428217	2.34	5.23	2.23	0.84	0.70	0.97
cg17670640	6	TAP2	9.03216E-09	2.27	9.62	4.24	0.81	0.66	0.95
cg07722274	9	SLC1A1	0.014695169	2.26	3.76	1.66	0.78	0.63	0.93
cg14522873	20	XRN2	0.002206904	2.55	4.18	1.64	0.77	0.62	0.93
cg12237591	19	AP1M1	0.032137812	2.36	3.20	1.35	0.77	0.62	0.93
cg07320819	13	RB1	3.27648E-07	4.10	5.28	1.29	0.76	0.60	0.92
cg21682474	13	N4BP2L2	0.001526208	3.14	3.72	1.19	0.75	0.58	0.91
cg19519384	6	TRIM27	4.98784E-12	2.52	10.74	4.26	0.79	0.64	0.94
cg25522894	2	PLB1	0.002663237	2.38	4.36	1.83	0.88	0.76	0.99
cg05845808	19	CCDC159; TMEM205	1.60198E-41	2.29	27.01	11.78	0.89	0.78	1.00
cg23418208	14	ZFYVE21; XRCC3	1.33455E-05	2.28	6.80	2.98	0.78	0.63	0.93
cg15388570	14	ADCK1	3.50053E-10	2.28	10.67	4.67	0.85	0.73	0.98
cg05637773	6	JARID2	1.2502E-06	2.08	8.78	4.23	0.75	0.59	0.91
cg22413205	8	PDP1	2.52529E-06	2.07	8.51	4.11	0.80	0.65	0.94
cg00418743	6	XPO5; POLH	4.08618E-05	2.02	7.49	3.71	0.82	0.68	0.96
cg16537756	20	PTGIS	9.90941E-06	2.08	7.82	3.76	0.78	0.63	0.94
cg10314670	19	PPM1N; RTN2	0.0024294	2.25	4.69	2.09	0.75	0.59	0.91
cg05386387	18	FBXO15	0.003054209	4.65	2.92	0.63	0.85	0.72	0.98
cg21543045	12	PRDM4	1.37795E-40	2.50	22.06	8.82	0.86	0.73	0.98
cg25874678	3	TMEM44	2.53212E-05	2.42	6.16	2.55	0.76	0.60	0.92
cg13096007	4	SOD3	0.000680513	2.04	5.93	2.90	0.77	0.61	0.92
cg17354807	6	AARS2	6.02701E-40	5.28	14.59	2.76	0.85	0.71	0.98
cg23339899	8	GPAA1	0.002629182	2.61	4.03	1.54	0.80	0.66	0.95
cg11620687	15	IMP3	0.000432719	2.01	6.32	3.15	0.77	0.61	0.92
cg04861263	15	FOXB1	2.47226E-40	5.44	15.54	2.86	0.80	0.65	0.95
cg21336547	19	AKAP8	0.000587605	3.06	4.10	1.34	0.77	0.61	0.92
cg24877391	2	PEX13; PUS10	1.02952E-07	7.84	4.52	0.58	0.80	0.65	0.95
cg26646290	12	CCDC53	2.20067E-10	5.01	6.19	1.24	0.86	0.73	0.98
cg12891667	3	NICN1	3.00354E-12	4.43	7.27	1.64	0.80	0.66	0.95
cg15617297	10	ANXA11	0.013154509	3.85	2.67	0.69	0.75	0.59	0.91
cg03859186	16	ZNF785	0.000753402	3.31	3.83	1.16	0.78	0.63	0.93
cg24767041	6	PSMB8	9.6375E-07	3.00	6.04	2.01	0.80	0.66	0.95
cg12104945	11	ARHGEF17	0.03630266	2.99	2.62	0.88	0.90	0.79	1.00
cg00859739	20	LSM14B	2.17556E-40	2.86	19.69	6.88	0.79	0.64	0.94
cg03436453	1	WASF2	6.29057E-39	2.81	14.71	5.23	0.87	0.75	0.99

cg05899224	18	SEC11C	3.63158E-05	2.53	5.76	2.28	0.77	0.61	0.92
cg11833275	1	KHDRBS1	0.001993677	2.47	4.34	1.76	0.76	0.60	0.92
cg01867111	2	ARL6IP6; PRPF40A	0.000202951	2.34	5.54	2.36	0.81	0.66	0.95
cg01573650	16	HIRIP3; INO80E	6.07921E-06	2.29	7.11	3.11	0.80	0.65	0.95
cg03745002	16	TOX3	3.75326E-07	2.22	8.46	3.81	0.79	0.64	0.94
cg19850348	15	PYGO1	0.010004007	2.15	4.18	1.94	0.81	0.66	0.95
cg24853904	5	SEMA5A	2.75989E-09	2.15	10.84	5.05	0.80	0.65	0.94
cg09703789	6	TUBE1	0.015461548	2.15	3.95	1.84	0.78	0.63	0.93
cg27435133	16	CBFA2T3	5.8839E-07	2.10	8.96	4.27	0.76	0.60	0.92
cg16851799	1	CASP9	0.000129466	2.07	6.64	3.20	0.84	0.70	0.97
cg21506655	2	NR4A2	2.50928E-10	2.36	10.32	4.36	0.85	0.72	0.98
cg15629719	13	SPRY2	0.000374402	2.39	5.18	2.16	0.76	0.60	0.92
cg09694403	14	DHRS7	0.007228681	2.85	3.38	1.18	0.76	0.60	0.92
cg16742990	6	RNF146	3.66105E-13	2.42	12.06	4.98	0.75	0.58	0.91
cg09239744	16	GRIN2A	0.022573346	2.52	3.20	1.27	0.81	0.66	0.95
cg16296356	17	RAD51L3	6.79023E-40	6.15	13.99	2.27	0.82	0.68	0.96
cg12927990	18	GRP	4.65386E-05	2.40	5.97	2.49	0.84	0.71	0.97
cg02639808	4	CCNI	2.98981E-07	4.55	5.07	1.12	0.75	0.59	0.91
cg16001418	19	HCST	1.32121E-38	2.56	14.27	5.56	0.75	0.59	0.91
cg10604703	9	WDR5	1.39643E-06	2.45	7.08	2.89	0.76	0.60	0.92
cg00747619	16	CPPED1	0.002055804	2.79	3.92	1.41	0.77	0.61	0.92
cg07706279	3	GORASP1; TTC21A	0.002414759	2.47	4.27	1.73	0.77	0.61	0.92
cg07849302	10	TCF7L2	1.02158E-06	2.85	6.27	2.20	0.82	0.68	0.96
cg19351189	16	SLC12A4	0.000151992	2.54	5.23	2.06	0.81	0.66	0.95
cg16909962	1	RAB4A	0.036695684	2.63	2.86	1.09	0.81	0.67	0.96
cg18590590	1	LRRC42; HSPB11	6.05625E-13	4.60	7.42	1.61	0.82	0.68	0.96
cg05338745	12	CCDC59	2.10056E-05	6.12	3.80	0.62	0.77	0.61	0.93
cg24143101	3	FNDC3B	5.60466E-40	5.37	14.62	2.72	0.79	0.64	0.94
cg26425904	15	OCA2	5.8411E-10	4.64	6.21	1.34	0.77	0.62	0.93
cg24153071	19	CCDC130	0.000108816	4.07	3.97	0.98	0.81	0.66	0.95
cg20640698	12	LOH12CR1; LOH12CR2	1.46105E-10	3.22	7.86	2.44	0.81	0.67	0.95
cg13528777	6	PHF1	0.001370197	3.03	3.84	1.27	0.76	0.60	0.92
cg18443229	22	NOL12	0.001704143	2.84	3.94	1.39	0.85	0.73	0.98
cg22244076	20	EIF6	1.28852E-05	2.83	5.55	1.96	0.85	0.72	0.98
cg06956240	4	SLC9B2	2.40891E-39	2.78	16.33	5.88	0.93	0.84	1.00
cg19041146	11	TMEM25; TTC36	0.032023419	2.68	2.89	1.08	0.76	0.60	0.92
cg06838921	16	APRT	0.030169184	2.62	2.96	1.13	0.76	0.60	0.92
cg14664714	12	STK38L	0.001974047	2.52	4.27	1.70	0.78	0.62	0.93
cg13215078	11	NPAS4	1.11696E-12	2.07	14.63	7.05	0.85	0.72	0.98
cg21933829	16	METTL9	0.026028171	2.07	3.82	1.85	0.87	0.75	0.99
cg16321228	10	PFKP	0.000326425	2.05	6.27	3.05	0.83	0.69	0.96
cg02153584	22	CCDC117	4.71402E-06	4.29	4.61	1.07	0.78	0.63	0.94
cg01999229	19	SNRNP70	0.012615439	2.20	3.96	1.80	0.75	0.59	0.91
cg10205852	7	CLDN12	1.70564E-09	2.50	9.10	3.65	0.76	0.60	0.92
cg23359173	7	PSPH; CCT6A	8.55209E-06	2.02	8.24	4.07	0.75	0.59	0.91
cg26362852	5	RNF145	4.90334E-43	5.09	22.73	4.46	0.76	0.60	0.92
cg19580633	5	RPL26L1	1.823E-08	4.54	5.62	1.24	0.85	0.73	0.98
cg07490279	1	ATAD3B	0.043980638	2.36	3.03	1.28	0.77	0.61	0.92
cg04630879	15	CDAN1	0.007193487	2.04	4.65	2.28	0.77	0.61	0.92
cg05957736	3	PHLDB2	1.8385E-09	3.46	6.94	2.01	0.81	0.66	0.95
cg19908534	15	FBN1	8.67605E-09	2.20	10.02	4.55	0.77	0.62	0.93
cg18415182	14	MAPK1IP1L	1.77985E-08	2.73	7.66	2.80	0.88	0.76	0.99
cg17043366	19	ZNF414	0.011735537	3.16	2.99	0.94	0.82	0.69	0.96
cg26262375	19	WDR62; THAP8	8.62464E-06	2.53	6.25	2.47	0.83	0.69	0.96

cg23539966	2	RTN4	0.015165857	2.38	3.56	1.49	0.76	0.60	0.92
cg03445574	2	SF3B1	6.23239E-05	2.01	7.31	3.63	0.75	0.59	0.91
cg16684668	5	WDR55	0.003113642	3.30	3.38	1.02	0.76	0.60	0.92
cg15017283	11	FOXRED1; SRPR	6.61922E-08	3.23	6.41	1.99	0.75	0.58	0.91
cg09804398	20	NINL	0.01546815	3.12	2.90	0.93	0.88	0.76	0.99
cg25169790	17	KIAA0195	1.08466E-11	2.83	9.36	3.30	0.81	0.66	0.95
cg06934911	5	MAT2B	1.41273E-06	2.71	6.44	2.38	0.75	0.59	0.91
cg00908189	16	PIGQ	3.44719E-08	2.50	8.15	3.26	0.75	0.59	0.91
cg18494399	11	SLC1A2	4.46161E-07	2.48	7.38	2.97	0.81	0.67	0.96
cg15810163	19	KRI1	0.028287502	2.30	3.34	1.45	0.90	0.79	1.00
cg00867273	16	ROGDI	2.2854E-40	2.21	23.29	10.52	0.78	0.63	0.93
cg07140217	7	ZYX	0.000103778	2.18	6.31	2.89	0.81	0.66	0.95
cg25471404	6	ABCC10	8.77848E-05	2.11	6.67	3.16	0.78	0.63	0.93
cg17448127	4	RPL34	3.59544E-07	2.92	6.43	2.20	0.78	0.63	0.93
cg12934804	14	ARG2	0.000884951	5.02	3.17	0.63	0.88	0.76	1.00
cg01691333	10	CREM	0.00017144	2.71	4.89	1.80	0.82	0.68	0.96
cg16761549	6	TMEM170B	0.011157524	3.64	2.80	0.77	0.82	0.68	0.96
cg03286969	7	AASS	0.003571087	2.11	4.84	2.29	0.75	0.59	0.91
cg08165558	14	DHRS4	1.11216E-05	2.10	7.68	3.66	0.76	0.60	0.92
cg09733424	1	NVL	8.11204E-06	2.23	7.22	3.24	0.89	0.77	1.00
cg01754828	6	ADGRB3	2.22526E-14	2.03	16.72	8.22	0.83	0.70	0.97
cg22196952	12	GRASP	2.53836E-05	2.92	5.23	1.79	0.84	0.70	0.97
cg02840367	8	FDFT1	9.95786E-06	2.16	7.44	3.45	0.77	0.62	0.93
cg18039556	22	RAB36	0.000811862	2.26	5.17	2.29	0.83	0.69	0.97
cg22513924	1	NMNAT2	1.13839E-40	2.53	22.23	8.80	0.78	0.63	0.93
cg05013027	6	GNL1	5.20701E-40	4.30	15.60	3.63	0.77	0.62	0.93
cg14959291	3	EPHA6	5.10483E-14	5.23	7.44	1.42	0.78	0.63	0.94
cg03466082	12	ZNF384	4.32394E-07	2.92	6.38	2.18	0.77	0.62	0.93
cg15524173	19	PLD3	0.005204489	2.72	3.63	1.34	0.78	0.63	0.94
cg18640183	5	P4HA2	0.003188261	2.71	3.84	1.42	0.75	0.58	0.91
cg26290478	17	EXOC7	6.15684E-05	2.69	5.28	1.97	0.81	0.66	0.95
cg06735806	14	MIPOL1	3.604E-39	2.43	17.08	7.03	0.81	0.66	0.95
cg22842854	12	HIP1R	9.79158E-39	2.35	15.70	6.68	0.78	0.63	0.94
cg11405279	15	SEMA4B	0.018668985	2.34	3.51	1.50	0.79	0.63	0.94
cg19735074	1	HIAT1	5.66679E-09	2.33	9.48	4.08	0.78	0.63	0.94
cg22491927	19	CACNA1A	1.04305E-05	2.05	7.96	3.88	0.77	0.61	0.92
cg13735965	19	ZNF616	2.28013E-38	2.40	13.94	5.81	0.77	0.62	0.93
cg14120359	9	SMARCA2	1.39355E-13	2.17	14.37	6.63	0.75	0.58	0.91
cg04653284	16	SH2B1	0.030747107	2.22	3.42	1.54	0.87	0.75	0.99
cg00625177	16	TXNL4B; DHX38	0.003866289	2.88	3.60	1.25	0.77	0.62	0.93
cg06627825	2	C1D	0.001144901	2.01	5.78	2.87	0.80	0.65	0.94
cg17971929	21	PSMG1	4.49563E-05	2.05	7.28	3.55	0.76	0.60	0.92
cg06867065	19	CLDND2	1.16535E-08	3.93	6.09	1.55	0.79	0.64	0.94
cg26312059	12	UBC	0.001701374	2.02	5.55	2.75	0.87	0.75	0.99
cg03787849	17	MAP3K14	0.003872479	2.08	4.87	2.34	0.80	0.66	0.95
cg16476890	4	RBM47	1.08736E-07	3.99	5.58	1.40	0.76	0.60	0.92
cg17771515	6	CNKSR3	0.002527478	2.12	5.00	2.36	0.75	0.58	0.91
cg24386890	2	HOXD1; HAGLR	7.63518E-05	2.65	5.27	1.98	0.80	0.65	0.95
cg07916071	1	PSMD4	1.53884E-12	4.19	7.58	1.81	0.82	0.68	0.96
cg14370961	14	BRF1	3.08738E-10	3.58	7.18	2.01	0.91	0.81	1.00
cg12727596	16	PAM16; CORO7	0.000165947	3.53	4.13	1.17	0.78	0.62	0.93
cg02058552	10	NET1	5.70687E-11	3.09	8.33	2.70	0.82	0.68	0.96
cg00482898	2	SNRPG	2.03321E-08	2.90	7.25	2.50	0.78	0.63	0.93
cg18469811	11	GUCY2E	0.033889619	2.79	2.77	0.99	0.76	0.60	0.92

cg16770712	3	SS18L2	2.1214E-12	2.71	10.19	3.76	0.77	0.61	0.92
cg23491124	18	PMAIP1	1.12914E-08	2.55	8.34	3.28	0.75	0.59	0.91
cg17846100	22	MCAT	0.000631527	2.50	4.76	1.90	0.83	0.70	0.97
cg20493960	11	AMOTL1	2.63548E-08	2.48	8.32	3.36	0.85	0.73	0.98
cg19045300	21	LSS; MCM3AP-AS1	0.00079438	2.47	4.72	1.91	0.76	0.60	0.92
cg16173736	12	RERG	2.08395E-11	2.36	11.13	4.71	0.81	0.66	0.95
cg07254066	16	IRX5	2.23544E-11	2.33	11.32	4.86	0.77	0.61	0.92
cg00575153	8	PLEC1	7.55405E-08	2.32	8.59	3.70	0.75	0.58	0.91
cg14356946	20	EIF2S2	3.02981E-14	2.24	14.24	6.36	0.75	0.58	0.91
cg00366818	10	ARMC4	0.036692874	2.19	3.37	1.54	0.80	0.65	0.95
cg23840219	6	SESN1	7.42104E-11	2.16	12.13	5.61	0.75	0.59	0.91
cg04200835	16	VKORC1	0.008759897	2.34	3.89	1.66	0.85	0.72	0.98
cg22758957	3	PSMD2	2.02034E-07	3.04	6.40	2.10	0.84	0.70	0.97
cg00212272	16	TSNAXIP1	7.19839E-08	2.52	7.86	3.12	0.78	0.63	0.94
cg05418736	16	RSPRY1; FAM192A	4.59381E-39	2.76	15.37	5.58	0.77	0.61	0.92
cg01529917	12	UBE2N	0.015395249	2.52	3.38	1.34	0.77	0.62	0.93
cg15933195	10	LRRC27	0.000329874	4.31	3.60	0.84	0.83	0.70	0.97
cg15759889	19	DDA1	0.001347516	2.36	4.71	2.00	0.79	0.64	0.94
cg01304814	3	PRKAR2A	1.47278E-06	2.30	7.58	3.29	0.78	0.62	0.93
cg04634693	4	MMAA	0.011508373	2.74	3.29	1.20	0.77	0.62	0.93
cg23709321	16	TMEM170A	0.047330314	2.76	2.64	0.96	0.82	0.68	0.96
cg14694176	8	TCEA1	2.96014E-38	7.32	9.10	1.24	0.88	0.76	1.00
cg11260709	17	EIF5A	1.59473E-09	3.97	6.47	1.63	0.82	0.68	0.96
cg19309149	9	GKAP1	8.70665E-10	3.89	6.65	1.71	0.76	0.60	0.92
cg03771255	4	RPS3A	0.01335417	3.26	2.89	0.89	0.77	0.61	0.92
cg06329261	14	TTC5	2.29626E-08	3.20	6.71	2.10	0.78	0.62	0.93
cg25096680	10	HIF1AN	0.001298543	2.61	4.31	1.65	0.83	0.70	0.97
cg11864882	18	PSTPIP2	0.031888751	2.43	3.12	1.29	0.76	0.61	0.92
cg18158043	14	AK7	5.60532E-12	2.42	11.22	4.64	0.82	0.68	0.96
cg26879696	10	VTI1A; ZDHHC6	0.016984623	2.32	3.60	1.55	0.77	0.61	0.92
cg27501451	6	BRD2	3.6016E-06	2.28	7.32	3.21	0.85	0.72	0.98
cg23737229	21	ADAMTS1	2.28715E-06	2.26	7.58	3.35	0.80	0.66	0.95
cg02161262	2	BIN1	4.18237E-11	2.02	13.83	6.86	0.79	0.64	0.94
cg25310430	7	WDR60	0.000717218	2.52	4.68	1.86	0.75	0.59	0.91
cg18379624	13	COG3	7.76384E-07	2.27	7.97	3.51	0.78	0.62	0.93
cg26575164	1	SNX27	3.63263E-41	2.53	23.94	9.45	0.78	0.63	0.93
cg22068773	1	SWT1; TRMT1L	0.028389464	2.13	3.64	1.71	0.76	0.60	0.92
cg08378782	11	RASGRP2	0.005323296	2.74	3.60	1.32	0.79	0.64	0.94
cg07015190	6	RPS6KA2	0.003594762	2.70	3.80	1.41	0.82	0.68	0.96
cg12014623	3	SEC22C	5.73961E-07	2.74	6.65	2.43	0.75	0.59	0.91
cg26545983	19	ELL	4.49493E-05	4.40	4.05	0.92	0.87	0.75	0.99
cg23395483	19	TMEM149	0.003404881	2.83	3.69	1.30	0.75	0.58	0.91
cg02547269	6	FOXF2	2.20342E-06	2.34	7.28	3.11	0.84	0.71	0.97
cg07941700	3	NEK4	2.44665E-06	3.20	5.54	1.73	0.83	0.69	0.96
cg24337497	6	CENPQ; MUT	5.7266E-07	2.96	6.25	2.12	0.78	0.63	0.93
cg20201475	3	MECOM	9.33751E-14	2.82	10.59	3.76	0.83	0.69	0.97
cg03087610	10	AIFM2	8.24195E-07	2.75	6.53	2.38	0.77	0.61	0.92
cg21939213	1	NRD1	0.002508182	2.71	3.93	1.45	0.76	0.60	0.92
cg25594636	1	YOD1	0.028552834	2.57	3.03	1.18	0.76	0.60	0.92
cg14235434	3	UBP1	1.37206E-07	2.51	7.67	3.05	0.85	0.73	0.98
cg09238756	1	HIPK1	8.36152E-07	2.31	7.78	3.37	0.85	0.73	0.98
cg23002761	1	FBLIM1	1.563E-09	2.22	10.57	4.77	0.76	0.60	0.92
cg10439765	20	SLC12A5	5.18154E-39	2.14	18.13	8.45	0.91	0.80	1.00
cg07229027	8	ATAD2	0.018243822	2.09	3.97	1.90	0.77	0.61	0.92

cg19139691	2	KDM3A	3.47669E-05	3.01	5.02	1.67	0.89	0.78	1.00
cg00342532	19	KCNC3	6.55111E-05	2.53	5.54	2.19	0.82	0.68	0.96
cg23504634	3	EIF4G1	0.005013711	3.50	3.12	0.89	0.85	0.72	0.98
cg04988224	19	RPL18; SPHK2	4.07019E-07	4.10	5.24	1.28	0.85	0.72	0.98
cg01050704	19	MZF1	0.00849977	2.33	3.92	1.68	0.79	0.64	0.94
cg18357291	3	NUP210	6.373E-11	4.66	6.59	1.41	0.80	0.65	0.94
cg14818026	1	NSUN4	0.018194032	2.36	3.49	1.48	0.79	0.64	0.94
cg10781048	15	SELS	5.22974E-12	2.28	12.16	5.34	0.75	0.58	0.91
cg26445041	11	RAB6A	0.000217055	3.44	4.11	1.19	0.78	0.63	0.93
cg12644625	22	SGSM1	1.24946E-07	3.22	6.27	1.94	0.85	0.73	0.98
cg26424013	16	DCUN1D3; LYRM1	0.037116649	3.11	2.55	0.82	0.77	0.61	0.92
cg14606680	17	FAM64A	0.000212613	2.85	4.64	1.63	0.85	0.72	0.98
cg26020522	12	APPL2	1.80983E-05	2.71	5.64	2.08	0.84	0.70	0.97
cg06734342	3	SACM1L	6.96699E-11	2.66	9.44	3.55	0.86	0.73	0.98
cg27105275	9	GCNT1	0.023165538	2.49	3.22	1.29	0.77	0.62	0.93
cg06213463	16	MT3	0.000632463	2.39	4.97	2.08	0.77	0.62	0.93
cg16619662	19	EID2	0.040549053	2.30	3.15	1.37	0.75	0.59	0.91
cg24174307	17	ATP5H; KCTD2	1.21839E-39	2.20	20.45	9.31	0.82	0.68	0.96
cg15709401	20	HSPC072	1.80044E-12	7.28	6.25	0.86	0.79	0.64	0.94
cg26776742	6	KPNA5	0.018013059	2.18	3.80	1.74	0.77	0.62	0.93
cg18216767	18	VPS4B	0.022464854	2.29	3.49	1.53	0.75	0.59	0.91
cg09799983	2	CYP1B1	9.32541E-39	3.71	12.41	3.34	0.79	0.64	0.94
cg15259947	6	MTHFD1L	0.00663151	2.74	3.52	1.28	0.75	0.58	0.91
cg05755010	1	ACP6	0.016598953	2.13	3.94	1.84	0.75	0.59	0.91
cg07847424	16	RHBDL1	0.001504035	2.35	4.69	2.00	0.83	0.70	0.97
cg23202277	17	MAPT	4.46412E-06	2.68	6.15	2.30	0.76	0.60	0.92
cg14389183	18	MBD1	8.67153E-15	2.08	16.41	7.88	0.89	0.78	1.00
cg23633626	1	DNTTIP2	0.009651084	3.52	2.89	0.82	0.80	0.65	0.95
cg09525523	12	DERA	1.57461E-13	2.19	14.13	6.45	0.80	0.65	0.95
cg04166546	6	DHX16	1.25759E-05	2.56	6.07	2.37	0.84	0.71	0.97
cg05121388	17	METTL2A	2.41814E-06	4.18	4.80	1.15	0.82	0.68	0.96
cg27063739	9	SEC16A	0.033659762	2.09	3.60	1.72	0.78	0.63	0.94
cg14515699	12	PPM1H	7.82067E-07	2.94	6.20	2.11	0.76	0.60	0.92
cg07318231	5	FBXW11	1.05961E-13	2.84	10.50	3.70	0.75	0.59	0.91
cg04110109	17	FKBP10; SC65	8.83786E-39	2.51	15.17	6.05	0.86	0.73	0.98
cg20407116	11	POLA2	2.6312E-38	4.68	10.15	2.17	0.84	0.71	0.97
cg25519033	11	TMEM80; DEAF1	0.037444008	2.87	2.68	0.93	0.82	0.68	0.96
cg03159396	6	TFAP2B	9.98869E-40	2.74	17.85	6.52	0.83	0.70	0.97
cg17070338	13	CARKD	0.008464634	2.66	3.50	1.32	0.85	0.71	0.98
cg03309253	19	LTBP4	8.65072E-06	2.10	7.77	3.70	0.86	0.74	0.99
cg08154698	19	LASS4	0.000245369	2.06	6.37	3.09	0.75	0.59	0.91
cg22551196	12	IKBIP; APAF1	0.000151592	2.40	5.51	2.29	0.76	0.60	0.92
cg09436851	3	LRCH3	1.07481E-05	2.90	5.50	1.90	0.78	0.62	0.93
cg25569234	6	PFDN6; WDR46	4.68822E-39	2.67	15.62	5.85	0.75	0.59	0.91
cg11748517	9	PDCL	1.2008E-06	2.20	8.10	3.68	0.87	0.75	0.99
cg19688118	5	CCDC112	2.00038E-14	2.04	16.65	8.15	0.83	0.69	0.97
cg04955406	2	ORMDL1; PMS1	0.020188177	2.50	3.28	1.31	0.77	0.61	0.92
cg15886919	7	METTL2B	0.011309825	2.91	3.16	1.08	0.76	0.60	0.92
cg16817229	21	MX1	3.49145E-06	2.88	5.86	2.04	0.77	0.61	0.92
cg26226802	4	GLRA3	1.54894E-05	2.77	5.59	2.01	0.75	0.59	0.91
cg14409958	8	ENPP2	2.85737E-15	2.54	12.64	4.97	0.76	0.60	0.92
cg15674575	1	FAM46C	1.03644E-08	2.42	8.82	3.64	0.78	0.63	0.93
cg04730071	17	LIMD2	0.005435967	2.37	4.07	1.72	0.76	0.60	0.92
cg09361488	1	DHRS3	6.15082E-05	2.35	5.99	2.54	0.80	0.65	0.94

cg17245735	1	NFIA	4.43961E-08	2.19	9.44	4.30	0.77	0.62	0.93
cg16629967	4	HNRNPD	0.004127091	2.12	4.74	2.23	0.79	0.64	0.94
cg15660832	1	PROX1	7.68933E-05	2.10	6.79	3.24	0.77	0.61	0.92
cg25240047	5	ADAMTS12	2.50792E-06	2.03	8.76	4.32	0.81	0.66	0.95
cg18680021	14	OTUB2	0.000112569	2.69	5.08	1.89	0.76	0.60	0.92
cg07334717	10	GLUD1; FAM35A	1.14949E-06	2.21	8.07	3.65	0.79	0.64	0.94
cg22493117	19	TRMT1; NACC1	0.002192984	2.73	3.96	1.45	0.75	0.59	0.91
cg12635019	15	PAK6	0.011507136	2.26	3.89	1.72	0.82	0.68	0.96
cg25178781	13	ARGLU1	1.07474E-11	2.31	11.70	5.07	0.77	0.61	0.92
cg14998457	2	SLC35F5	1.10364E-13	4.57	7.73	1.69	0.81	0.66	0.95
cg07857142	2	STK39	0.010154469	2.35	3.79	1.61	0.76	0.60	0.92
cg08959305	17	TMEM97	0.01349805	2.07	4.21	2.03	0.81	0.67	0.95
cg13794009	6	DDAH2	7.09664E-07	2.90	6.29	2.17	0.83	0.70	0.97
cg22256664	16	ZNF598	0.015446732	2.33	3.62	1.55	0.77	0.61	0.92
cg09151181	14	SETD3; CCNK	9.50837E-05	5.20	3.66	0.70	0.81	0.67	0.95
cg25314532	22	POLR2F	0.002644531	2.14	4.90	2.29	0.75	0.59	0.91
cg08866589	5	KIAA0141	0.00048608	2.86	4.35	1.52	0.76	0.60	0.92
cg07690783	2	GFPT1	0.047362709	2.88	2.56	0.89	0.75	0.58	0.91
cg00108164	2	ACP1; SH3YL1	5.38093E-09	3.82	6.34	1.66	0.77	0.61	0.92
cg06152533	3	LRRN1	5.22973E-38	3.80	9.83	2.59	0.80	0.65	0.95
cg15518042	1	ZNF643	0.003767914	2.62	3.88	1.48	0.76	0.60	0.92
cg07900968	19	ZFP82	4.29498E-05	2.58	5.60	2.17	0.76	0.60	0.92
cg13233534	1	UAP1	0.004349522	2.57	3.87	1.51	0.75	0.59	0.91
cg16600637	3	CBLB	2.55546E-08	2.27	9.25	4.08	0.79	0.64	0.94
cg01587050	17	BRCA1; NBR2	3.37089E-07	2.19	8.67	3.96	0.77	0.61	0.92
cg14942464	14	TMED10	4.13721E-08	2.17	9.62	4.43	0.76	0.60	0.92
cg18116548	2	PPP1R1C	8.71406E-39	2.16	16.99	7.85	0.80	0.65	0.95
cg24530250	11	PHOX2A	7.54773E-07	2.14	8.63	4.04	0.75	0.58	0.91
cg02468435	9	NINJ1	5.71612E-06	2.07	8.15	3.94	0.80	0.66	0.95
cg22474865	17	WDR81	0.002514754	2.16	4.89	2.26	0.75	0.59	0.91
cg00220905	5	RGMB	0.00016159	2.95	4.61	1.56	0.89	0.78	1.00
cg18453621	9	LMX1B	0.002128773	2.71	3.99	1.47	0.75	0.58	0.91
cg09338809	11	ZBED5	0.000266711	3.48	4.03	1.16	0.75	0.59	0.91
cg26708817	4	NEUROG2	1.54331E-06	2.46	7.03	2.86	0.85	0.72	0.98
cg11829072	8	TRMT12	8.57162E-14	4.80	7.61	1.59	0.79	0.64	0.94
cg09818520	1	VPS72	0.000228185	2.80	4.67	1.67	0.80	0.65	0.94
cg14726348	11	KAT5	0.00153496	3.47	3.51	1.01	0.80	0.65	0.95
cg19937480	1	PRDX1	4.87052E-10	2.92	8.17	2.80	0.77	0.62	0.93
cg02306127	19	ZNF461	5.03909E-40	5.01	15.00	3.00	0.91	0.81	1.00
cg14331245	17	MED24	7.23436E-14	4.20	8.11	1.93	0.77	0.61	0.92
cg10938441	3	ABHD14B	1.62455E-38	3.77	11.55	3.06	0.77	0.61	0.92
cg10897909	6	CCNC	6.73905E-38	3.72	9.53	2.56	0.87	0.75	0.99
cg13255538	13	CENPJ	0.015719208	3.39	2.77	0.82	0.86	0.74	0.99
cg19090210	3	SEMA3F	5.20044E-39	2.66	15.49	5.82	0.82	0.68	0.96
cg06649808	10	SEC24C	0.036255731	2.26	3.27	1.45	0.83	0.69	0.96
cg14174215	2	KLHL23	0.003871217	2.16	4.66	2.16	0.81	0.66	0.95
cg26322231	7	ZC3HAV1L	0.001525739	2.15	5.17	2.41	0.75	0.58	0.91
cg11596498	19	ADCK4	0.019515076	2.03	4.08	2.00	0.76	0.60	0.92
cg27071312	5	KIAA0947	6.17507E-43	2.03	35.66	17.59	0.77	0.62	0.93
cg18480233	2	CRIM1	1.89816E-14	4.60	8.00	1.74	0.85	0.72	0.98
cg07006158	16	EXOSC6	0.000632893	2.04	5.99	2.93	0.77	0.62	0.93
cg11804724	1	DNAH14	0.011546435	2.42	3.63	1.50	0.79	0.64	0.94
cg25386745	1	UTP11L	6.90724E-39	2.11	17.86	8.48	0.80	0.65	0.95
cg12565250	10	EIF4EBP2	5.17498E-09	2.96	7.48	2.52	0.77	0.61	0.92

cg06824297	21	RWDD2B	7.70314E-06	3.00	5.47	1.82	0.81	0.66	0.95
cg16413687	12	ALX1	0.000409219	2.43	5.06	2.08	0.82	0.68	0.96
cg05431548	17	LUC7L3	2.4996E-39	2.20	19.13	8.72	0.85	0.71	0.98
cg05439191	3	OGG1	8.08593E-06	7.14	3.85	0.54	0.77	0.61	0.92
cg08697244	7	DAGLB	1.0463E-10	4.09	6.89	1.68	0.88	0.77	1.00
cg26008390	19	PLEKHG2	1.017E-08	3.69	6.33	1.71	0.81	0.67	0.95
cg06591892	19	AP2S1	1.77644E-13	2.84	10.34	3.64	0.92	0.82	1.00
cg07944399	2	STK36; RNF25	3.46766E-05	4.29	4.16	0.97	0.82	0.67	0.96
cg09782621	19	ZNF444	1.79076E-43	3.53	26.64	7.54	0.81	0.67	0.96
cg25674838	17	DUSP3	6.59164E-06	3.48	5.01	1.44	0.81	0.67	0.95
cg23928824	14	GALNTL1	3.37448E-38	3.26	11.13	3.41	0.81	0.66	0.95
cg18679299	14	TM9SF1	8.52534E-06	3.02	5.41	1.80	0.81	0.66	0.95
cg06794268	19	HNRNPUL1	2.53426E-08	2.68	7.69	2.87	0.79	0.64	0.94
cg17509537	15	SERF2	0.001498757	2.63	4.23	1.61	0.79	0.64	0.94
cg23359276	5	PCDHGA4; PCDHGA12; PCDHGA11; PCDHGA11; PCDHGA9; PCDHGA1; PCDHGB1; PCDHGC3; PCDHGB6; PCDHGB3; PCDHGB7; PCDHGC3; PCDHGA6; PCDHGA8; PCDHGA10; PCDHGA5; PCDHGB4; PCDHGA3; PCDHGC3; PCDHGA2; PCDHGA7; PCDHGB2; PCDHGB5	0.000264318	2.50	5.09	2.03	0.85	0.72	0.98
cg02379642	9	INPP5E	1.34819E-05	2.42	6.37	2.63	0.77	0.61	0.92
cg15028282	1	TTLL7	1.45484E-14	2.40	13.19	5.50	0.77	0.62	0.93
cg15603959	3	PBRM1; GNL3	1.22444E-05	2.25	6.97	3.10	0.77	0.61	0.92
cg15755662	7	TTYH3	0.003668153	2.17	4.68	2.16	0.87	0.75	0.99
cg11552078	19	TIMM50	4.7557E-48	2.31	47.45	20.52	0.86	0.73	0.98
cg08607907	7	SGCE; PEG10	3.94336E-05	2.42	5.97	2.46	0.76	0.60	0.92
cg08274661	17	CDC27	6.05247E-06	2.86	5.73	2.00	0.89	0.78	1.00
cg01096478	12	DIP2B	0.0001002	3.26	4.47	1.37	0.76	0.60	0.92
cg23538765	19	UBA2	1.95165E-08	2.40	8.72	3.64	0.79	0.64	0.94
cg22334543	19	FZR1	4.11255E-39	2.01	19.68	9.77	0.90	0.80	1.00
cg18037107	7	VOPP1	0.008635084	2.33	3.92	1.68	0.79	0.64	0.94
cg16477190	15	BRUNOL6	3.92422E-38	3.47	10.61	3.06	0.78	0.62	0.93
cg10598353	6	HSPA1A; HSPA1L	0.003391091	2.21	4.60	2.08	0.78	0.63	0.93
cg12077581	22	PANX2	7.50015E-06	2.76	5.83	2.11	0.84	0.70	0.97
cg05388821	10	FAM160B1	4.7098E-38	2.94	11.15	3.79	0.75	0.59	0.91
cg03243965	19	HSH2D	1.40516E-05	2.28	6.80	2.98	0.90	0.79	1.00
cg01466862	2	ZC3H8	0.010734952	2.98	3.13	1.05	0.81	0.66	0.95
cg22876214	13	RBM26	3.24945E-40	5.12	15.44	3.01	0.83	0.69	0.96
cg10495115	11	NDUFS8	7.35224E-07	3.72	5.36	1.44	0.87	0.75	0.99
cg03416888	20	PSMA7; SS18L1	2.50443E-07	3.32	5.98	1.80	0.77	0.61	0.92
cg05298677	6	ETV7	6.76208E-05	3.23	4.60	1.43	0.77	0.61	0.92
cg03902642	1	ROBLD3; UBQLN4	0.000242704	3.02	4.41	1.46	0.77	0.62	0.93
cg16916914	17	ITGB4	0.0009283	2.80	4.19	1.50	0.78	0.62	0.93
cg24718035	12	TFCP2	1.9608E-10	2.72	8.93	3.28	0.88	0.77	1.00
cg09360495	17	NEK8	8.70436E-14	2.38	12.78	5.38	0.86	0.73	0.98
cg08223129	12	NEMP1	3.48095E-11	2.28	11.51	5.06	0.76	0.60	0.92
cg25300805	2	NUP35	1.37838E-12	2.26	12.74	5.63	0.77	0.62	0.93
cg15992333	5	REEP5	2.15874E-09	2.12	11.12	5.23	0.86	0.74	0.99
cg11820558	20	CDS2; PCNA	0.001375933	2.52	4.42	1.75	0.76	0.60	0.92
cg06373505	16	HCFC1R1; THOC6	6.4398E-06	2.94	5.60	1.91	0.78	0.63	0.93

cg05541396	8	PURG; WRN	0.020418224	2.47	3.30	1.34	0.80	0.65	0.94
cg26459683	15	PIAS1	2.76256E-05	3.37	4.74	1.41	0.83	0.69	0.96
cg09698548	8	CA8	0.015205521	2.43	3.50	1.44	0.89	0.77	1.00
cg13656078	19	CALM3	0.024795375	2.15	3.68	1.72	0.79	0.64	0.94
cg20912169	8	ADHFE1	1.69211E-07	2.89	6.70	2.32	0.83	0.69	0.97
cg13793678	4	OCIAD1	1.11547E-10	2.86	8.69	3.04	0.77	0.62	0.93
cg17704177	6	SFRS3	1.25503E-05	2.34	6.63	2.83	0.78	0.62	0.93
cg04110421	17	NBR2; BRCA1	2.66346E-08	2.02	10.88	5.38	0.77	0.61	0.92
cg23065793	3	SEC62	1.17601E-12	3.85	7.97	2.07	0.88	0.76	0.99
cg00873037	8	NEIL2	6.61998E-39	2.87	14.47	5.05	0.76	0.61	0.92
cg03582798	7	MLXIPL	3.10955E-10	4.51	6.39	1.42	0.81	0.66	0.95
cg26076960	13	MED4	1.10371E-39	4.14	14.81	3.58	0.78	0.62	0.93
cg03128635	2	ATOH8	7.00872E-07	4.05	5.15	1.27	0.82	0.68	0.96
cg20824211	3	DLG1	7.79323E-38	4.03	9.06	2.25	0.75	0.58	0.91
cg18691565	10	ACBD5	4.14588E-40	3.92	16.37	4.18	0.87	0.75	0.99
cg09931891	16	AP1G1	3.9742E-10	3.19	7.69	2.41	0.87	0.75	0.99
cg20678247	22	SUN2	0.000131387	2.71	4.99	1.84	0.82	0.68	0.96
cg06951601	19	ZNF799	0.032489311	2.54	2.99	1.18	0.79	0.64	0.94
cg23812361	11	E2F8	0.00054458	2.45	4.90	2.00	0.83	0.70	0.97
cg21733597	15	IDH3A	0.000320188	2.45	5.13	2.10	0.75	0.59	0.91
cg23836487	2	KANSL3	0.020750626	2.32	3.49	1.51	0.82	0.68	0.96
cg22777309	6	SCUBE3	3.19441E-11	2.17	12.34	5.68	0.77	0.62	0.93
cg04260924	2	PMS1; ORMDL1	1.32361E-07	2.16	9.21	4.27	0.75	0.59	0.91
cg24454144	20	SLC32A1	0.000819447	2.10	5.62	2.68	0.84	0.70	0.97
cg19488260	11	SPON1	0.009880627	2.09	4.33	2.07	0.84	0.70	0.97
cg16128701	8	FABP5	2.29107E-10	2.08	12.37	5.93	0.75	0.59	0.91
cg01460882	12	FAM60A	0.001512968	2.80	4.02	1.44	0.84	0.71	0.97
cg27445106	1	SNIP1	8.57366E-15	2.44	13.10	5.37	0.83	0.69	0.97
cg13251256	11	CCDC84	0.000316488	3.87	3.78	0.97	0.85	0.71	0.98
cg01013370	19	HMHA1	2.27152E-08	2.34	8.94	3.83	0.76	0.60	0.92
cg21454656	22	RBX1	4.45045E-09	3.80	6.40	1.69	0.89	0.78	1.00
cg23912763	12	PWP1	0.003547485	2.88	3.63	1.26	0.79	0.64	0.94
cg11930768	3	CRELD1	7.82501E-05	3.71	4.22	1.14	0.81	0.66	0.95
cg09516764	12	EP400NL	4.08203E-06	3.12	5.50	1.76	0.88	0.76	0.99
cg17585343	11	HSD17B12	0.000329374	2.58	4.86	1.88	0.77	0.62	0.93
cg18962915	19	TSEN34	2.14899E-38	5.71	9.93	1.74	0.77	0.62	0.93
cg06208824	14	PSMC6	0.007939408	2.08	4.48	2.15	0.77	0.61	0.92
cg23912878	14	MBIP	1.3742E-13	2.18	14.27	6.55	0.83	0.69	0.96
cg05020625	6	TTBK1	1.54343E-09	3.85	6.57	1.71	0.81	0.67	0.95
cg21952419	13	INTS6	8.91538E-14	5.58	7.19	1.29	0.76	0.60	0.92
cg03235119	5	ZNF346	8.77316E-41	4.20	17.94	4.27	0.81	0.67	0.95
cg06325700	2	FAM228B; SF3B6	1.92591E-06	3.98	4.96	1.25	0.77	0.62	0.93
cg12099727	7	WBSCR22; DNAJC30	5.3032E-05	3.89	4.23	1.09	0.78	0.62	0.93
cg03630364	7	NUB1	2.40344E-05	3.58	4.61	1.29	0.75	0.59	0.91
cg00584636	2	DNPEP	4.28856E-07	3.30	5.87	1.78	0.86	0.74	0.99
cg14922223	1	NUF2; RGS5	0.038946089	3.29	2.45	0.75	0.79	0.64	0.94
cg07663813	8	ZNF704	0.000463981	2.85	4.38	1.54	0.78	0.62	0.93
cg05188158	17	METT10D	7.32575E-06	2.84	5.71	2.01	0.93	0.84	1.00
cg23849946	9	KDM4C	0.000127526	2.68	5.04	1.88	0.76	0.60	0.92
cg12711743	1	LRRC8C; FLJ27354	0.0004474	2.68	4.61	1.72	0.99	0.95	1.00
cg04029586	6	EHMT2	0.026922148	2.15	3.63	1.69	0.77	0.61	0.92
cg25421835	5	CNOT8	0.001258175	2.59	4.34	1.68	0.75	0.59	0.91
cg20689859	2	EHBP1	0.000171095	2.88	4.67	1.62	0.75	0.59	0.91
cg11535522	22	PPPDE2; XRCC6	4.69422E-07	4.01	5.26	1.31	0.75	0.59	0.91

cg16199144	15	TRIP4; KIAA0101	1.00958E-07	2.40	8.16	3.40	0.80	0.65	0.95
cg05348768	1	CYB5R1	4.08353E-05	2.66	5.46	2.05	0.85	0.72	0.98
cg19896040	22	MED15	0.034267111	2.48	3.03	1.22	0.77	0.61	0.92
cg05009619	7	NDUFB2	0.017538677	2.12	3.93	1.85	0.83	0.69	0.96
cg05709063	5	CENPK; PPWD1	3.44577E-38	3.78	10.46	2.77	0.75	0.59	0.91
cg04018299	1	ADAM15	0.00973411	2.72	3.38	1.24	0.78	0.63	0.93
cg12630974	1	MED18	3.32034E-12	7.03	6.22	0.88	0.75	0.59	0.91
cg27566521	1	CTNNBIP1	0.00618475	2.32	4.09	1.76	0.81	0.67	0.96
cg07532155	1	PTPRF	2.91983E-09	3.36	6.97	2.08	0.86	0.73	0.98
cg17223643	2	TTC7A; MCFD2	0.045526237	2.11	3.38	1.60	0.75	0.58	0.91
cg24160036	20	RBM38	0.000187568	2.10	6.35	3.03	0.84	0.71	0.97
cg02658739	5	TNPO1	7.22562E-06	2.54	6.30	2.48	0.78	0.62	0.93
cg14686008	11	PDHX; APIP	0.013836581	3.80	2.67	0.70	0.81	0.67	0.95
cg20784314	20	YWHAB	1.26593E-12	2.36	12.04	5.10	0.85	0.71	0.98
cg10106268	4	SLC30A9	5.02346E-38	6.64	8.58	1.29	0.88	0.76	1.00
cg00746864	17	PITPNC1	4.0854E-39	6.10	11.86	1.94	0.80	0.66	0.95
cg17319142	2	C1QL2	1.8096E-38	5.09	10.42	2.05	0.78	0.62	0.93
cg18957973	2	KIAA1310	1.55571E-10	4.68	6.42	1.37	0.79	0.64	0.94
cg13055374	1	SSBP3	1.44987E-39	3.78	14.90	3.94	0.90	0.80	1.00
cg04917181	8	TSPYL5	6.00767E-11	3.64	7.45	2.05	0.85	0.72	0.98
cg15356195	7	ACTB	0.000673785	3.57	3.71	1.04	0.84	0.71	0.97
cg17777561	17	FXR2; SHBG	0.001370048	2.98	3.89	1.31	0.75	0.59	0.91
cg25417344	19	MRI1	0.005258628	2.87	3.49	1.21	0.85	0.72	0.98
cg20150565	20	ZNFX1	1.25136E-41	2.62	25.01	9.56	0.84	0.71	0.98
cg14351634	17	WRAP53; TP53	6.37943E-05	2.54	5.54	2.18	0.75	0.59	0.91
cg05367846	22	MICAL3	1.63558E-45	2.16	42.42	19.63	0.96	0.89	1.00
cg13207467	1	CSRP1	0.01866122	2.15	3.83	1.78	0.78	0.62	0.93
cg27269921	22	MN1	0.007478555	2.10	4.46	2.12	0.75	0.58	0.91
cg25987077	20	POFUT1; PLAGL2	0.014515649	2.10	4.09	1.95	0.76	0.60	0.92
cg27392024	18	CCDC11	0.024234669	2.96	2.81	0.95	0.75	0.59	0.91
cg11416243	10	CUGBP2	0.007922148	2.13	4.35	2.04	0.77	0.61	0.92
cg13876048	2	PQLC3	0.012953533	2.31	3.74	1.62	0.82	0.68	0.96
cg00101453	22	GATSL3	0.000639594	3.14	4.00	1.27	0.76	0.60	0.92
cg03771878	16	PHKG2	2.42235E-05	2.48	6.01	2.42	0.77	0.61	0.92
cg06891548	1	GGPS1; ARID4B	2.33443E-38	6.90	9.48	1.37	0.88	0.77	1.00
cg08863848	7	HOXA11	3.05412E-09	4.52	5.97	1.32	0.83	0.70	0.97
cg01849260	5	TBCA	2.68952E-39	3.41	14.64	4.30	0.82	0.68	0.96
cg07408690	9	ZFAND5	0.048697813	2.17	3.23	1.49	0.79	0.64	0.94
cg16605745	12	RPAP3	1.46777E-42	3.52	24.20	6.87	0.83	0.70	0.97
cg10892375	14	RAB2B; TOX4	0.000842316	2.16	5.41	2.50	0.82	0.68	0.96
cg20124620	19	GPR108	0.000521807	3.27	3.97	1.21	0.78	0.63	0.94
cg06044117	11	FAM76B; CEP57	1.91395E-08	3.78	6.11	1.62	0.75	0.59	0.91
cg27537561	12	VDR	6.31386E-08	3.19	6.47	2.03	0.78	0.63	0.93
cg00044463	1	GLT25D2	2.86959E-38	2.85	12.15	4.27	0.79	0.64	0.94
cg21226485	18	MYL12B	0.002273408	2.85	3.83	1.34	0.80	0.66	0.95
cg26886427	19	YIPF2	6.25909E-12	2.84	9.48	3.34	0.76	0.60	0.92
cg02891574	19	ZNF446	1.39225E-10	2.43	10.17	4.19	0.75	0.59	0.91
cg26831488	11	RBM7	0.027317358	2.42	3.22	1.33	0.78	0.63	0.93
cg07801681	16	ARL6IP1	0.005783243	2.29	4.18	1.82	0.82	0.68	0.96
cg16395712	17	NDEL1	0.025207335	2.22	3.54	1.59	0.78	0.63	0.94
cg20573820	6	SYNGAP1	0.0469218	2.21	3.19	1.44	0.87	0.75	0.99
cg10531688	3	THPO; CHRD	1.41353E-39	2.17	20.41	9.42	0.82	0.69	0.96
cg07099627	19	LPPR2	0.000835114	2.15	5.45	2.53	0.78	0.62	0.93
cg06151988	20	DLGAP4	6.01797E-39	3.53	13.29	3.77	0.79	0.64	0.94

cg13096688	17	PLEKHM1	0.004073094	2.09	4.83	2.31	0.76	0.60	0.92
cg17322839	9	TMOD1	2.42974E-39	4.44	13.48	3.03	0.81	0.66	0.95
cg09848695	5	FAM172A	1.92608E-06	3.02	5.82	1.93	0.83	0.69	0.96
cg19156395	1	PEF1	5.05812E-08	2.21	9.29	4.20	0.75	0.59	0.91
cg18721249	7	CAV1	7.30266E-39	3.56	12.96	3.64	0.77	0.61	0.92
cg02464895	19	RPS9	1.35102E-06	3.37	5.51	1.64	0.81	0.66	0.95
cg27306034	1	FAM189B	0.000324328	2.29	5.47	2.39	0.80	0.65	0.95
cg17080709	1	GNAI3	1.31635E-39	3.48	15.52	4.46	0.87	0.75	0.99
cg01181758	16	OGFOD1; NUDT21	6.90224E-13	2.29	12.71	5.54	0.81	0.67	0.96
cg10865087	10	BICC1	2.21961E-13	2.27	13.27	5.85	0.75	0.58	0.91
cg12072333	7	WDR91	3.16126E-05	5.96	3.75	0.63	0.83	0.70	0.97
cg14825076	12	RNF41	0.048370017	2.16	3.25	1.50	0.78	0.63	0.93
cg00048823	14	OSGEP; APEX1	6.2549E-05	3.79	4.24	1.12	0.83	0.69	0.97
cg15708019	4	WDR19	0.000585167	2.86	4.29	1.50	0.78	0.63	0.93
cg21577705	19	MBOAT7	0.018117597	2.19	3.78	1.73	0.76	0.60	0.92
cg04719537	11	CNTN5	6.23088E-11	3.37	7.80	2.31	0.80	0.65	0.95
cg17143565	17	SLFN5	2.94018E-38	3.30	11.28	3.42	0.78	0.63	0.94
cg14197576	6	KIF13A	0.003478912	2.72	3.80	1.40	0.75	0.59	0.91
cg07574621	3	XPC; LSM3	0.003833611	2.61	3.88	1.48	0.75	0.59	0.91
cg07587588	8	TPD52	2.60266E-41	2.56	24.25	9.46	0.75	0.59	0.91
cg02328010	1	RYR2	1.35098E-07	2.44	7.90	3.23	0.76	0.60	0.92
cg06490744	11	ST3GAL4	0.000245033	2.41	5.31	2.20	0.76	0.60	0.92
cg19425870	11	STIM1	0.000499898	2.39	5.07	2.12	0.82	0.68	0.96
cg24668364	17	UBB	3.49171E-06	2.29	7.30	3.19	0.85	0.72	0.98
cg01009697	9	NTRK2	2.3269E-08	2.25	9.39	4.18	0.80	0.65	0.95
cg06808983	17	G6PC3	1.29371E-07	2.24	8.77	3.91	0.89	0.78	1.00
cg06639387	1	TRIM11	2.72584E-05	2.15	7.05	3.28	0.76	0.60	0.92
cg06383531	19	HDGF2	7.39895E-05	2.12	6.69	3.15	0.80	0.65	0.94
cg05843800	3	SUMF1	0.000381585	2.12	5.94	2.81	0.76	0.60	0.92
cg24976104	22	PRR5; ARHGAP8	0.040410452	2.14	3.40	1.59	0.79	0.64	0.94
cg02237342	22	ADORA2A	8.12043E-06	2.61	6.09	2.33	0.86	0.74	0.99
cg14185025	14	EML1	6.88688E-08	3.30	6.31	1.91	0.82	0.68	0.96
cg08393660	6	RNF8	0.000291196	3.14	4.25	1.36	0.81	0.67	0.95
cg16331823	6	FLOT1; IER3	4.32835E-07	2.55	7.18	2.81	0.75	0.58	0.91
cg17182540	12	DDX55	0.001586118	2.82	3.98	1.41	0.82	0.68	0.96
cg24591761	15	MYO9A; SENP8	0.008594083	2.36	3.87	1.64	0.77	0.61	0.92
cg10743145	19	ERCC1	5.90301E-07	3.23	5.86	1.81	0.83	0.69	0.97
cg20721338	15	PML	0.002020682	2.18	4.94	2.26	0.79	0.64	0.94
cg27026673	2	MAP4K3	8.25333E-39	4.08	12.18	2.99	0.80	0.66	0.95
cg04677871	16	ZNF821	0.000231412	2.36	5.44	2.30	0.78	0.63	0.93
cg07798595	1	SEP15; HS2ST1	2.76044E-09	2.29	9.91	4.33	0.78	0.62	0.93
cg11804248	15	GALK2; COPS2	4.47999E-06	4.15	4.68	1.13	0.77	0.61	0.93
cg15059389	1	MIIP	9.40956E-38	4.80	8.34	1.74	0.84	0.71	0.97
cg22865986	2	FBXO36; TRIP12	8.65829E-11	4.25	6.80	1.60	0.78	0.63	0.93
cg08646372	13	USPL1	5.31857E-12	3.24	8.56	2.64	0.79	0.64	0.94
cg18270629	11	PAX6	0.000320617	3.19	4.18	1.31	0.84	0.70	0.97
cg19982658	14	AP1G2	0.036813354	2.84	2.70	0.95	0.82	0.68	0.96
cg15589156	11	RAB3IL1	0.008674167	2.75	3.39	1.23	0.80	0.65	0.94
cg17147121	19	ZNF836	1.20614E-07	2.54	7.62	2.99	0.79	0.64	0.94
cg02205181	10	NFKB2	8.27296E-15	2.38	13.52	5.68	0.82	0.68	0.96
cg01538659	1	POGZ	0.001706049	2.28	4.77	2.09	0.86	0.74	0.99
cg09166659	1	SAMD11	0.009586367	2.25	4.00	1.78	0.76	0.60	0.92
cg13792025	7	RABGEF1	0.00532253	2.50	3.88	1.55	0.80	0.65	0.95
cg26389465	11	PTS	0.002087201	3.41	3.45	1.01	0.81	0.67	0.96

cg13275176	2	SEMA4C	1.95171E-43	3.10	28.08	9.05	0.84	0.70	0.97
cg08034643	17	AKAP1	0.013920493	2.38	3.60	1.51	0.78	0.63	0.93
cg00546594	21	ATP5O	0.025066169	2.14	3.69	1.73	0.75	0.58	0.91
cg26211360	12	LRRC43	7.13839E-10	2.28	10.45	4.58	0.79	0.64	0.94
cg21695485	11	RHOD	9.70146E-40	3.01	17.00	5.64	0.76	0.60	0.92
cg20910303	4	SLC10A7	5.03836E-10	2.61	9.05	3.47	0.80	0.65	0.95
cg26298722	19	DMPK	0.00695983	2.33	4.01	1.72	0.76	0.60	0.92
cg08048517	12	KIAA0528	1.02777E-37	7.55	7.50	0.99	0.79	0.64	0.94
cg09453752	5	MARCH3	7.28564E-05	3.56	4.33	1.22	0.83	0.70	0.97
cg07231649	1	VPS45	0.001644728	3.28	3.60	1.10	0.76	0.60	0.92
cg20419523	4	RASGEF1B	4.44519E-38	3.27	10.68	3.26	0.80	0.65	0.95
cg23473726	19	ZNF324	0.018774771	2.90	2.96	1.02	0.90	0.79	1.00
cg08073882	4	JAKMIP1	0.020573513	2.37	3.42	1.44	0.81	0.67	0.96
cg13472656	22	ADRBK2	6.31011E-40	2.37	20.40	8.62	0.82	0.68	0.96
cg01070148	19	BCL3	0.000410625	2.19	5.67	2.59	0.77	0.62	0.93
cg13714459	15	IREB2	0.021426332	2.13	3.81	1.79	0.81	0.66	0.95
cg01966344	22	DEPDC5	5.86883E-09	2.90	7.58	2.62	0.88	0.76	0.99
cg14701410	12	SLC4A8	0.000711579	4.97	3.23	0.65	0.84	0.71	0.97
cg24105243	3	OXSM; NGLY1	0.034168852	2.74	2.81	1.03	0.77	0.61	0.92
cg06658864	19	ZNF700	0.010037357	2.54	3.55	1.40	0.86	0.73	0.98
cg19622755	2	MCFD2; TTC7A	0.000435326	2.17	5.71	2.64	0.84	0.70	0.97
cg10088519	17	VAMP2	0.023250635	2.13	3.74	1.75	0.88	0.76	0.99
cg10729426	19	ZNF549	1.25603E-07	2.18	9.09	4.16	0.79	0.64	0.94
cg12266551	19	TOMM40	0.011057067	2.19	4.05	1.86	0.76	0.60	0.92
cg27249422	17	DHX58	0.000703914	2.68	4.44	1.66	0.79	0.64	0.94
cg17525428	19	SHANK1	3.3055E-11	3.19	8.26	2.59	0.83	0.70	0.97
cg19125323	17	SFRS2	0.016035371	2.47	3.41	1.38	0.76	0.60	0.92
cg25680942	5	NREP	1.54648E-11	2.96	8.93	3.02	0.81	0.66	0.95
cg12749132	1	C2CD4D	2.81952E-06	5.75	4.27	0.74	0.87	0.75	0.99
cg14260522	7	BAZ1B	2.61777E-41	4.51	19.00	4.21	0.83	0.69	0.97
cg27225571	6	MRPL18; TCP1	5.06009E-07	3.89	5.32	1.37	0.80	0.66	0.95
cg00370820	22	GTPBP1	3.11116E-09	3.56	6.71	1.89	0.80	0.66	0.95
cg07983652	1	HDGF	6.25949E-38	3.40	9.99	2.94	0.77	0.62	0.93
cg18172830	17	WFIKKN2	1.87522E-38	3.22	12.10	3.76	0.75	0.59	0.91
cg21145136	15	SH3GL3	0.002515513	3.03	3.64	1.20	0.80	0.66	0.95
cg01849156	20	CDK5RAP1	0.000577457	2.97	4.18	1.41	0.75	0.58	0.91
cg07203258	10	DDX50	1.08356E-05	2.92	5.48	1.88	0.79	0.64	0.94
cg12858895	14	SLC8A3	7.62074E-39	2.68	14.80	5.52	0.84	0.71	0.97
cg06749592	2	EFHD1	0.01663186	2.54	3.32	1.30	0.75	0.58	0.91
cg23687194	6	ID4	1.37492E-06	2.53	6.87	2.71	0.85	0.72	0.98
cg00696023	3	GMPS	0.001048519	2.38	4.77	2.00	0.79	0.64	0.94
cg10583297	15	DUOX1	2.8746E-07	2.32	8.12	3.50	0.75	0.59	0.91
cg17960268	9	CBWD1	1.09072E-13	2.29	13.34	5.82	0.85	0.72	0.98
cg20648939	18	RNMT	0.045635078	2.20	3.22	1.46	0.78	0.62	0.93
cg07596314	3	SHISA5	4.82597E-07	2.19	8.50	3.87	0.80	0.65	0.94
cg16443970	10	PAX2	0.005608551	2.03	4.83	2.37	0.76	0.60	0.92
cg00391481	8	FAM91A1	5.75888E-06	3.23	5.28	1.63	0.75	0.59	0.91
cg19849093	2	GALNT14	4.29662E-07	2.66	6.91	2.60	0.75	0.59	0.91
cg15048437	19	ZNF260	0.003701207	2.34	4.30	1.84	0.80	0.66	0.95
cg11039361	11	MARK2	1.51882E-11	2.43	10.89	4.49	0.76	0.60	0.92
cg14114033	7	SYPL1	0.00010394	2.37	5.73	2.41	0.80	0.65	0.94
cg00765710	1	PGBD2	2.75948E-40	4.48	16.20	3.61	0.95	0.87	1.00
cg04010086	21	DSCR3	0.020892751	4.03	2.46	0.61	0.82	0.68	0.96
cg05360949	19	BCKDHA; EXOSC5	1.27089E-14	2.58	12.13	4.70	0.82	0.69	0.96

cg12904950	1	NPHP4	4.83569E-38	4.40	9.48	2.16	0.81	0.66	0.95
cg16277479	1	PDPN	3.35642E-08	2.77	7.40	2.67	0.75	0.58	0.91
cg27332938	10	ZNF511; TUBGCP2	3.80537E-08	2.09	10.16	4.85	0.81	0.67	0.95
cg15079565	19	TYK2	3.62532E-06	3.58	5.08	1.42	0.77	0.62	0.93
cg23363832	3	RBP1	1.48291E-05	2.40	6.39	2.66	0.81	0.67	0.95
cg15378253	2	FARP2	5.29713E-44	4.53	25.79	5.70	0.89	0.78	1.00
cg14676825	17	PROCA1	2.4532E-39	3.30	14.97	4.54	0.77	0.61	0.92
cg17015511	11	RDX	4.47107E-06	3.03	5.58	1.84	0.78	0.63	0.93
cg22782268	13	GPC5	0.000278877	2.60	4.89	1.88	0.78	0.63	0.93
cg13880677	11	ATHL1	0.000813531	2.41	4.82	2.00	0.78	0.63	0.93
cg26185926	15	BLOC1S6	0.032378823	2.39	3.15	1.32	0.81	0.67	0.95
cg14926717	14	FLJ43390	6.13245E-07	2.36	7.68	3.25	0.81	0.66	0.95
cg26509139	1	MRPL20	5.76974E-07	2.31	7.90	3.42	0.79	0.64	0.94
cg21377598	22	DGCR6L	0.002131259	2.25	4.73	2.10	0.80	0.66	0.95
cg04607235	12	APOLD1	4.17352E-05	2.24	6.50	2.90	0.75	0.59	0.91
cg21283714	1	TMCC2	0.035096066	2.13	3.50	1.65	0.76	0.60	0.92
cg21127184	4	RWDD4A	1.06861E-44	2.07	41.24	19.88	0.78	0.63	0.94
cg18029778	7	MLL5	7.55438E-41	3.14	20.25	6.45	0.79	0.64	0.94
cg17576266	5	MATR3	0.002049914	2.76	3.96	1.44	0.83	0.69	0.96
cg25607294	22	GCAT	2.46923E-07	2.36	7.99	3.38	0.79	0.64	0.94
cg05730369	12	MON2	5.40025E-10	2.35	10.17	4.34	0.83	0.69	0.97
cg05031521	16	SOX8	4.55656E-38	3.10	10.91	3.52	0.85	0.73	0.98
cg00285026	16	TMEM159; DNAH3	0.043231339	2.02	3.59	1.77	0.80	0.65	0.95
cg15963901	7	VWC2	2.02212E-07	2.27	8.44	3.71	0.77	0.61	0.92
cg05808399	5	TRIM23; TRAPPC13	2.1828E-08	3.12	6.84	2.19	0.80	0.65	0.95
cg18453441	7	CDK6	0.003945492	2.44	4.10	1.68	0.76	0.60	0.92
cg13849422	20	FAM83D	1.09184E-05	2.29	6.85	2.99	0.83	0.70	0.97
cg00221525	16	ZNF668	1.03031E-38	2.62	14.51	5.54	0.90	0.80	1.00
cg13298682	22	ZNRF3	1.69548E-41	2.36	26.32	11.14	0.81	0.67	0.95
cg07696310	16	MAP1LC3B; FBXO31	9.49015E-05	2.37	5.78	2.44	0.85	0.71	0.98
cg14579362	19	RPS16	0.000628523	2.85	4.28	1.50	0.81	0.67	0.95
cg05192759	19	ANKRD24	4.38007E-08	2.25	9.12	4.05	0.85	0.73	0.98
cg12996119	17	AATF	0.001939598	2.50	4.31	1.73	0.80	0.66	0.95
cg25520999	1	UCK2	0.002851101	2.28	4.54	1.99	0.78	0.62	0.93
cg00978521	2	CDC42EP3	8.24991E-05	3.26	4.52	1.39	0.85	0.72	0.98
cg14786398	19	ZNF543	0.038055322	2.27	3.22	1.42	0.81	0.67	0.96
cg16414762	1	CAMTA1	1.06227E-38	6.56	10.54	1.61	0.79	0.64	0.94
cg23258678	1	NR5A2	7.69662E-40	3.43	16.35	4.76	0.78	0.62	0.93
cg12746717	2	HDAC4	0.003508901	2.53	4.01	1.58	0.84	0.71	0.97
cg26161170	6	RPP21	2.0695E-13	2.11	14.91	7.08	0.84	0.71	0.97
cg02126098	17	MAP3K3	0.002236493	3.32	3.48	1.05	0.79	0.64	0.94
cg25713309	19	OCEL1	0.034528036	2.25	3.31	1.47	0.75	0.58	0.91
cg05889842	3	FAM194A	0.002164499	2.73	3.97	1.46	0.76	0.60	0.92
cg27431596	1	ZNF697	0.013647647	2.29	3.74	1.63	0.79	0.64	0.94
cg03847009	21	SYNJ1	5.57399E-05	2.13	6.78	3.18	0.76	0.60	0.92
cg18727717	2	ERLEC1; GPR75; ASB3	0.002477617	2.82	3.82	1.35	0.85	0.72	0.98
cg12518410	2	SH3RF3	2.44277E-07	4.86	4.99	1.03	0.79	0.64	0.94
cg15933935	1	MOBKL2C	2.55861E-41	3.06	22.00	7.19	0.92	0.82	1.00
cg25571184	12	METTL1; FAM119B	1.43293E-38	2.72	13.63	5.01	0.78	0.63	0.94
cg10690327	3	ACY1	1.73103E-39	2.59	17.58	6.79	0.86	0.74	0.99
cg15088822	5	DIAPH1	0.02596345	2.59	3.06	1.18	0.89	0.78	1.00
cg19977408	4	PGRMC2	2.31088E-39	2.44	17.79	7.30	0.86	0.74	0.99
cg11407066	1	RPS8	3.67459E-07	2.42	7.64	3.15	0.76	0.60	0.92

cg23792679	7	PSMC2	1.05418E-38	2.33	15.65	6.71	0.77	0.62	0.93
cg02395768	19	ATP5SL	0.000498404	2.26	5.38	2.39	0.77	0.62	0.93
cg13614659	20	SNX5	1.73522E-06	2.23	7.80	3.49	0.75	0.58	0.91
cg06815419	15	BTBD1	0.000969267	2.20	5.24	2.38	0.80	0.65	0.94
cg06187252	5	CETN3	0.000167782	2.13	6.30	2.96	0.81	0.67	0.96
cg05716166	8	RALYL	0.003126717	2.57	4.02	1.56	0.81	0.66	0.95
cg09990790	4	COX18	1.31431E-11	2.19	12.51	5.71	0.84	0.71	0.97
cg12486121	11	PPFIBP2	0.000107845	2.73	5.03	1.85	0.78	0.63	0.93
cg01815035	1	XPR1	1.48213E-08	2.09	10.63	5.10	0.75	0.59	0.91
cg14322427	6	WASF1; CDC40	3.5522E-05	3.30	4.72	1.43	0.78	0.62	0.93
cg15689069	19	ZNF581	0.027857499	2.19	3.54	1.62	0.81	0.67	0.95
cg08689322	12	OS9	6.74679E-05	2.35	5.95	2.53	0.79	0.64	0.94
cg25875515	5	ZCCHC10	0.002800864	2.56	4.07	1.59	0.84	0.71	0.97
cg10860002	12	ANAPC7	3.72623E-14	2.10	15.65	7.46	0.78	0.63	0.94
cg01180236	16	EEF2K	2.31797E-07	2.11	9.33	4.43	0.84	0.70	0.97
cg26404748	14	ACIN1	0.000108248	2.98	4.70	1.58	0.75	0.59	0.91
cg19965088	3	VIPR1	0.001483029	2.01	5.65	2.81	0.82	0.69	0.96
cg12276298	10	ECD; FAM149B1	0.007863224	3.34	3.04	0.91	0.75	0.58	0.91
cg26767154	4	NUP54	1.83993E-38	3.88	11.25	2.90	0.80	0.65	0.94
cg04199977	16	NUBP1	0.000114909	2.56	5.29	2.07	0.83	0.69	0.96
cg00064255	1	LRRC8C	0.003090668	2.45	4.19	1.71	0.75	0.59	0.91
cg07935151	6	LSM2	6.44988E-10	2.21	10.95	4.96	0.83	0.70	0.97
cg04939555	13	SPG20	9.87406E-11	2.76	9.00	3.26	0.81	0.67	0.95
cg20701145	12	DUSP16	0.026036093	3.45	2.56	0.74	0.78	0.63	0.93
cg06005309	2	STRADB; TRAK2	3.56562E-38	3.24	11.08	3.42	0.82	0.68	0.96
cg00091986	11	MDK	3.5127E-15	3.16	10.39	3.29	0.88	0.76	0.99
cg09506515	20	SNHG11	1.98786E-09	3.04	7.57	2.49	0.87	0.75	0.99
cg03839252	13	FOXO1	0.006246352	2.91	3.39	1.17	0.77	0.61	0.92
cg24404401	16	CALB2	0.000832165	2.77	4.26	1.54	0.87	0.75	0.99
cg13567310	18	NDUFV2-AS1; ANKRD12	0.004821193	2.40	4.07	1.70	0.76	0.60	0.92
cg15946251	11	KCNJ5	0.000118475	2.38	5.66	2.37	0.83	0.70	0.97
cg14769207	21	SIM2	6.58174E-39	2.32	16.57	7.14	0.76	0.60	0.92
cg20294304	6	HMGA1	2.37512E-15	2.30	14.49	6.31	0.82	0.68	0.96
cg15166561	18	SMAD7	0.004782255	2.05	4.86	2.37	0.78	0.63	0.93
cg03426252	9	CRAT; PPP2R4	4.38045E-12	3.17	8.74	2.75	0.76	0.60	0.92
cg05943975	12	MLEC	0.002484428	2.90	3.74	1.29	0.77	0.61	0.92
cg13264662	14	COX16	0.007097117	2.08	4.55	2.18	0.77	0.61	0.92
cg08251337	16	CENPN	5.89982E-05	2.03	7.23	3.56	0.80	0.66	0.95
cg07709080	15	ANP32A	0.007094446	2.39	3.91	1.64	0.76	0.60	0.92
cg08822537	1	ZBTB8A	3.9545E-40	2.15	22.84	10.61	0.80	0.66	0.95
cg10487770	5	RAB3C	9.24627E-46	2.81	36.07	12.86	0.89	0.77	1.00
cg21883754	19	TTYH1	0.000137671	2.50	5.33	2.13	0.77	0.61	0.92
cg06797525	6	HIST1H2AM; HIST1H2BO	0.016635292	2.94	2.98	1.02	0.81	0.67	0.95
cg17331461	7	ATXN7L1	1.66328E-39	3.64	14.92	4.10	0.85	0.73	0.98
cg27085741	7	CLCN1	1.16643E-09	3.43	7.09	2.07	0.76	0.60	0.92
cg19127283	3	TRIM71	0.029196116	2.11	3.64	1.72	0.77	0.62	0.93
cg15317173	18	KIAA1632	0.021557914	2.38	3.38	1.42	0.76	0.60	0.92
cg02021606	21	SIK1	1.49124E-39	2.99	16.44	5.50	0.84	0.71	0.97
cg02558537	11	CWF19L2	7.19134E-38	4.64	8.79	1.89	0.81	0.66	0.95
cg22602910	9	TRIM14	9.1874E-07	3.71	5.31	1.43	0.79	0.64	0.94
cg11369026	2	ITSN2	8.82485E-13	3.59	8.36	2.33	0.78	0.63	0.93
cg02232875	1	TXLNA	7.86057E-13	3.53	8.47	2.40	0.81	0.66	0.95
cg23167148	9	TOR2A	1.12395E-09	3.45	7.07	2.05	0.78	0.62	0.93

cg10304637	6	NRN1	9.60039E-12	2.87	9.28	3.24	0.85	0.71	0.98
cg17194243	13	FAM48A	0.000568563	2.85	4.31	1.52	0.80	0.66	0.95
cg15937634	12	BCL7A	5.37157E-07	2.62	6.94	2.65	0.77	0.62	0.93
cg26500063	1	MEX3A	2.322E-09	2.36	9.61	4.07	0.85	0.72	0.98
cg12326308	15	FAM227B; DTWD1	4.31626E-07	3.69	5.50	1.49	0.75	0.59	0.91
cg00490421	12	CPM	6.01523E-06	2.09	7.99	3.82	0.79	0.64	0.94
cg05928650	5	ITGA2	0.026461627	2.15	3.64	1.69	0.90	0.79	1.00
cg07866476	16	CBFB	1.10643E-06	2.07	8.89	4.30	0.84	0.70	0.97
cg24484856	12	SLC35E3	0.036753446	2.01	3.73	1.86	0.81	0.67	0.95
cg08242069	12	ADIPOR2	1.26955E-37	6.83	7.35	1.08	0.87	0.75	0.99
cg06458358	8	ARHGEF10	0.001856745	2.04	5.42	2.65	0.80	0.66	0.95
cg07050555	12	FBRSL1	0.031975104	2.16	3.51	1.63	0.79	0.64	0.94
cg05888082	3	DZIP3; KIAA1524	1.50408E-12	3.99	7.78	1.95	0.76	0.60	0.92
cg26954533	19	ZNF266	0.001776083	2.46	4.41	1.79	0.77	0.62	0.93
cg25436985	22	XRCC6; PPPDE2	0.024292169	2.72	2.98	1.09	0.81	0.67	0.96
cg09252805	1	ZBTB40	6.79288E-39	3.18	13.71	4.32	0.89	0.77	1.00
cg01395760	16	KIF22	6.5568E-08	3.03	6.70	2.21	0.79	0.64	0.94
cg14492773	5	GPBP1	4.98026E-42	2.99	24.45	8.17	0.90	0.80	1.00
cg22992573	12	VEZT; FGD6	1.4522E-39	2.90	16.73	5.77	0.77	0.62	0.93
cg00920484	8	RNF19A	0.000141941	2.81	4.82	1.71	0.84	0.71	0.97
cg00529181	12	ESYT1	1.73761E-06	2.80	6.20	2.21	0.76	0.60	0.92
cg12983110	1	DOCK7	6.64508E-06	2.52	6.37	2.53	0.75	0.59	0.91
cg17168820	19	JOSD2	2.46161E-10	2.43	9.99	4.11	0.82	0.69	0.96
cg26648054	17	DLG4	0.000282413	2.42	5.23	2.16	0.89	0.78	1.00
cg17414580	3	KPNA4	0.016021825	2.31	3.64	1.58	0.77	0.61	0.92
cg10526733	15	TARSL2	4.25144E-39	2.14	18.53	8.65	0.82	0.68	0.96
cg00597366	16	GPT2	0.002020142	2.08	5.22	2.50	0.75	0.59	0.91
cg09832551	6	RING1	0.022665622	2.07	3.90	1.89	0.79	0.64	0.94
cg18428563	13	FAM124A	1.12988E-06	2.06	8.91	4.32	0.82	0.69	0.96
cg06641938	1	UBXN11	1.125E-06	2.84	6.26	2.21	0.83	0.69	0.96
cg23653835	1	PUM1	1.37609E-08	3.10	6.99	2.25	0.75	0.59	0.91
cg20328917	12	TMTC3; CEP290	3.68352E-07	2.21	8.52	3.85	0.80	0.66	0.95
cg02891332	18	TWSG1	0.000287424	2.88	4.50	1.56	0.91	0.81	1.00
cg21184585	2	SEPT10; ANKRD57	9.37481E-07	2.26	7.93	3.51	0.77	0.62	0.93
cg12684684	2	ARPC2	0.038000313	2.91	2.65	0.91	0.80	0.65	0.94
cg03000603	19	DHX34	3.4001E-06	3.14	5.52	1.76	0.83	0.69	0.97
cg15485113	9	SAXO1; RRAGA	0.01984315	2.27	3.58	1.57	0.76	0.60	0.92
cg20415529	4	POLR2B	3.23923E-39	2.03	20.05	9.90	0.87	0.75	0.99
cg16029838	7	NUDCD3	0.000260378	3.83	3.85	1.00	0.78	0.63	0.94
cg17229496	11	TMEM109	4.73544E-39	3.61	13.51	3.74	0.81	0.66	0.95
cg09859272	12	RFC5	0.004152268	2.30	4.32	1.88	0.77	0.62	0.93
cg04054012	13	THSD1	9.62908E-05	2.13	6.53	3.06	0.77	0.62	0.93
cg01991785	17	RPTOR	7.50857E-13	2.33	12.45	5.35	0.78	0.63	0.93
cg26734620	12	CS	0.023370122	2.36	3.37	1.43	0.77	0.61	0.92
cg19016924	16	IST1	0.00018188	2.40	5.45	2.28	0.87	0.75	0.99
cg25175469	15	FAM63B	0.000317579	2.76	4.63	1.68	0.76	0.60	0.92
cg03556393	12	HOXC10	4.41918E-11	2.84	8.98	3.16	0.75	0.58	0.91
cg09134682	17	FLCN	2.4474E-06	2.46	6.87	2.79	0.78	0.63	0.93
cg07006526	22	SMTN	0.047203772	2.39	2.95	1.23	0.82	0.69	0.96
cg22541679	13	EDNRB	6.06982E-41	4.23	18.34	4.33	0.85	0.72	0.98
cg06656344	1	NTNG1	1.45697E-05	3.64	4.69	1.29	0.88	0.76	1.00
cg16232979	19	TPM4	4.8876E-38	3.59	10.13	2.82	0.94	0.85	1.00
cg17642145	8	PPP3CC	0.000236853	3.22	4.24	1.32	0.81	0.67	0.96
cg01560739	3	OSBPL10; ZNF860	3.48886E-06	3.13	5.53	1.77	0.81	0.66	0.95

cg15894039	8	INTS8	0.000156929	2.69	4.96	1.84	0.81	0.67	0.96
cg25036234	2	EFR3B	0.001927697	2.64	4.12	1.56	0.87	0.75	0.99
cg19943978	12	CLEC2B	1.19946E-38	2.58	14.39	5.58	0.79	0.64	0.94
cg08080008	16	NDUFB10	0.000426787	2.51	4.89	1.94	0.78	0.62	0.93
cg04465974	1	SUCO	0.000724539	2.51	4.69	1.87	0.77	0.61	0.92
cg20649047	14	NUMB	0.034230179	2.34	3.19	1.36	0.79	0.64	0.94
cg21691267	18	ZNF24	0.033014524	2.32	3.23	1.39	0.92	0.83	1.00
cg19686855	7	NDUFA5	0.00760996	2.20	4.22	1.92	0.77	0.61	0.92
cg12950434	20	CNBD2	5.80298E-13	2.12	14.38	6.79	0.85	0.72	0.98
cg13909453	3	PLD1	7.20095E-11	2.10	12.70	6.05	0.76	0.60	0.92
cg18963750	6	TUBB	0.020925375	2.09	3.89	1.86	0.75	0.58	0.91
cg18892239	6	BAT3	4.1769E-10	2.09	12.12	5.81	0.75	0.59	0.91
cg22322187	17	FN3KRP	2.85339E-39	2.03	20.24	9.96	0.81	0.67	0.95
cg10207042	12	RAD51AP1	0.018816068	2.03	4.10	2.02	0.78	0.63	0.93
cg17217189	17	IGF2BP1	0.017600047	2.06	4.07	1.97	0.90	0.80	1.00
cg14805136	1	SLC39A1	3.56207E-05	2.92	5.12	1.75	0.75	0.59	0.91
cg23597375	22	NHP2L1	0.009065596	2.84	3.30	1.16	0.79	0.64	0.94
cg25494665	13	DGKH	0.001097345	2.18	5.24	2.41	0.86	0.73	0.98
cg16713690	19	ZNF17	2.75023E-09	2.03	11.82	5.81	0.75	0.58	0.91
cg05796909	17	SENP3	0.007539009	2.17	4.28	1.97	0.77	0.62	0.93
cg21493666	11	PPME1; C2CD3	0.006700124	2.58	3.68	1.42	0.81	0.66	0.95
cg24451981	15	BUB1B	8.35583E-10	4.00	6.56	1.64	0.82	0.68	0.96
cg04300632	17	RNASEK	1.09609E-38	3.53	12.42	3.52	0.78	0.63	0.94
cg17953633	17	MRM1	2.54367E-11	2.50	10.38	4.16	0.81	0.67	0.96
cg00761861	14	VTI1B	0.001611641	3.33	3.58	1.07	0.77	0.62	0.93
cg17495178	4	PTPN13	1.46303E-14	5.22	7.65	1.46	0.82	0.69	0.96
cg27315947	21	GART; SON	9.65995E-05	2.72	5.07	1.86	0.80	0.65	0.94
cg19664712	6	CDKAL1	0.000110111	2.30	5.91	2.57	0.79	0.64	0.94
cg09522027	2	PPP1CB	0.000495761	2.19	5.58	2.55	0.81	0.66	0.95
cg06102063	9	CENPP; NOL8	0.007016271	2.55	3.70	1.45	0.75	0.58	0.91
cg14626490	9	NUP188; DOLK	0.007472754	2.68	3.53	1.32	0.81	0.66	0.95
cg15837503	17	MRPL12	5.51282E-06	2.51	6.45	2.57	0.75	0.59	0.91
cg14614793	9	IKBKAP	4.72854E-41	6.12	17.02	2.78	0.81	0.67	0.96
cg09882656	5	DAB2	4.61898E-15	5.12	7.86	1.54	0.76	0.60	0.92
cg06516150	12	ACACB	2.47673E-06	3.65	5.12	1.40	0.78	0.63	0.93
cg09215152	11	PPP2R1B	4.77307E-06	3.23	5.33	1.65	0.77	0.61	0.92
cg21726313	1	GPSM2	9.38416E-40	3.07	16.90	5.51	0.84	0.71	0.97
cg18173908	7	KLHDC10	1.00524E-39	3.05	16.84	5.51	0.83	0.70	0.97
cg06745957	11	LIPT2	1.78229E-38	3.00	12.59	4.20	0.83	0.69	0.97
cg03704756	20	PRNP	3.03639E-38	2.89	11.96	4.13	0.79	0.64	0.94
cg25992610	17	NLK	3.39058E-38	2.69	12.26	4.55	0.78	0.62	0.93
cg17447240	7	PDGFA	2.44819E-08	2.62	7.87	3.00	0.82	0.68	0.96
cg08966155	3	RPL35A; IQCG	7.78482E-11	2.45	10.27	4.19	0.82	0.69	0.96
cg05029288	4	UGDH	0.020463381	2.19	3.71	1.69	0.76	0.60	0.92
cg18372367	12	TNFRSF1A	0.000113211	2.07	6.70	3.23	0.75	0.58	0.91
cg11441935	19	ZNF566	3.30593E-06	2.06	8.47	4.12	0.77	0.62	0.93
cg03000271	6	SLC39A7; RXRB	1.08159E-07	3.34	6.16	1.84	0.77	0.61	0.92
cg20407381	2	PKP4; CCDC148	0.018632604	2.16	3.82	1.77	0.76	0.60	0.92
cg00713939	8	NAPRT1	7.81434E-42	2.88	24.31	8.44	0.79	0.64	0.94
cg13412003	19	ZNF792	1.47528E-08	2.22	9.73	4.39	0.84	0.71	0.97
cg22846109	17	DHRS13	0.007669666	2.49	3.73	1.50	0.85	0.72	0.98
cg24904303	19	ZC3H4	8.8763E-09	3.16	7.00	2.21	0.77	0.61	0.92
cg23651146	3	APPL1	2.68808E-07	2.87	6.61	2.30	0.79	0.64	0.94
cg02579736	3	FANCD2; CIDECP	0.000546275	2.73	4.47	1.64	0.77	0.61	0.92

cg15732812	1	DYRK3	0.008620122	3.14	3.12	0.99	0.80	0.65	0.94
cg16018184	19	ECH1	0.000186026	2.52	5.18	2.06	0.76	0.60	0.92
cg15248904	19	UQCRFS1	0.012329494	2.25	3.88	1.72	0.82	0.68	0.96
cg25701418	3	MAGI1	5.11338E-38	3.74	9.92	2.65	0.86	0.74	0.99
cg00465476	15	DUOX2	1.8338E-05	4.43	4.25	0.96	0.79	0.65	0.94
cg05254098	19	MKNK2	4.18203E-38	4.13	9.87	2.39	0.79	0.64	0.94
cg13529912	6	ELOVL5	7.55883E-06	3.90	4.70	1.20	0.78	0.62	0.93
cg07936037	6	SSR1	3.48665E-08	3.75	6.01	1.60	0.80	0.65	0.95
cg08304478	17	ASGR1	4.2193E-07	2.69	6.85	2.55	0.77	0.62	0.93
cg18764438	3	SNX4	8.89683E-10	2.44	9.53	3.90	0.77	0.62	0.93
cg06213964	6	FLJ22536	3.59927E-07	2.37	7.82	3.29	0.78	0.63	0.94
cg16657816	16	BCL7C	0.001054629	2.37	4.79	2.02	0.79	0.64	0.94
cg13187788	17	DLX3	6.57765E-09	2.34	9.37	4.01	0.80	0.65	0.95
cg17895626	17	PRPF8	0.017836326	2.00	4.22	2.11	0.77	0.61	0.92
cg06751596	5	SKP1	1.11949E-42	2.41	30.06	12.49	0.78	0.63	0.93
cg10583382	11	ELP4; IMMP1L	1.13591E-06	2.31	7.63	3.30	0.79	0.64	0.94
cg19891271	1	SSR2	3.49195E-05	3.05	4.97	1.63	0.77	0.62	0.93
cg24722405	5	PAIP2	0.003916523	2.31	4.33	1.87	0.76	0.60	0.92
cg16963632	1	DENND1B	0.027221653	2.23	3.47	1.56	0.75	0.58	0.91
cg01996304	16	ZNF668; ZNF646	0.015585515	2.83	3.09	1.09	0.76	0.60	0.92
cg10338465	15	CIB2	0.002384969	2.32	4.54	1.96	0.77	0.61	0.92
cg23017654	17	GHDC	3.16218E-39	3.20	14.81	4.63	0.77	0.61	0.92
cg03678754	3	RAB5A	3.05431E-06	3.64	5.08	1.40	0.78	0.62	0.93
cg11952622	19	ZNF324B	0.017129083	2.37	3.51	1.48	0.79	0.64	0.94
cg17016559	22	ATXN10	1.20455E-38	4.38	11.40	2.60	0.87	0.75	0.99
cg14461522	10	NPM3	3.29748E-05	3.77	4.42	1.17	0.75	0.59	0.91
cg09781971	12	MDM2	0.000192451	4.74	3.60	0.76	0.87	0.75	0.99
cg20978010	12	SETD1B	0.000250414	3.73	3.91	1.05	0.75	0.59	0.91
cg21864071	16	SPATA33; CHMP1A	9.49551E-06	3.00	5.40	1.80	0.79	0.64	0.94
cg04446777	10	BTRC	0.002946207	2.71	3.87	1.43	0.78	0.63	0.93
cg03637781	3	CTDSPL	7.51867E-41	2.70	21.96	8.15	0.79	0.64	0.94
cg24176566	12	ATP2A2	0.00058539	2.38	5.02	2.11	0.78	0.63	0.94
cg23481221	20	TOMM34	1.54258E-07	2.21	8.85	4.00	0.75	0.58	0.91
cg26660102	2	PDK1	1.18731E-09	2.10	11.60	5.54	0.82	0.68	0.96
cg13813874	22	KLHL22	0.002323444	2.00	5.44	2.72	0.89	0.77	1.00
cg24005131	12	TCP11L2	0.044625022	2.63	2.76	1.05	0.78	0.63	0.94
cg18345506	14	PSEN1	0.007244729	2.06	4.59	2.22	0.78	0.63	0.94
cg16739396	1	ATG4C	3.99116E-11	3.66	7.50	2.05	0.81	0.66	0.95
cg08726071	20	GINS1	0.006415221	2.53	3.76	1.48	0.81	0.66	0.95
cg13822158	19	MEIS3	1.28167E-06	2.03	9.09	4.48	0.81	0.67	0.96
cg02291533	19	LRFN3	0.013309855	2.14	4.05	1.89	0.83	0.69	0.96
cg25730717	7	PDIA4	2.10983E-10	7.09	5.60	0.79	0.83	0.69	0.96
cg05279284	14	DHRS1	0.011872967	2.52	3.50	1.39	0.77	0.61	0.92
cg12500437	17	FAM104A	1.48148E-09	4.04	6.42	1.59	0.75	0.59	0.91
cg05857825	20	MAPRE1	2.961E-07	2.24	8.45	3.77	0.82	0.69	0.96
cg24705402	4	ARFIP1	1.1332E-13	2.26	13.55	5.99	0.85	0.72	0.98
cg18639052	11	ZDHHC5	0.001189174	2.15	5.29	2.46	0.76	0.60	0.92
cg25600136	5	NADK2	0.00081959	2.79	4.26	1.53	0.77	0.62	0.93
cg16860267	18	MPPE1	3.41601E-08	2.33	8.85	3.80	0.86	0.74	0.99
cg11952076	9	DOLK; NUP188	0.001415511	2.89	3.95	1.37	0.78	0.63	0.93
cg14282407	17	TRAPPC1; CNTROB	6.99888E-38	7.78	7.95	1.02	0.83	0.70	0.97
cg09432792	16	GNAO1	1.99038E-12	3.23	8.79	2.72	0.84	0.70	0.97
cg14020129	2	GRHL1	1.04768E-38	2.76	14.04	5.10	0.81	0.66	0.95
cg18026227	8	ZFHX4	6.14181E-15	2.62	12.14	4.63	0.77	0.61	0.92

cg08397339	19	PIP5K1C	0.001022828	2.32	4.91	2.11	0.88	0.76	1.00
cg22700360	17	MSI2	0.002637957	2.23	4.67	2.09	0.81	0.67	0.96
cg07941711	15	LACTB	8.3716E-13	2.04	15.20	7.46	0.76	0.60	0.92
cg03559973	19	ZNF226	3.06943E-13	2.00	16.08	8.02	0.75	0.59	0.91
cg14022620	16	ITFG1; PHKB	9.02821E-10	2.29	10.28	4.48	0.85	0.72	0.98
cg04199943	6	HLA-L	4.28471E-05	3.53	4.50	1.28	0.79	0.64	0.94
cg09336228	16	DDX19A	0.008708313	2.20	4.15	1.88	0.79	0.64	0.94
cg07742499	19	SUV420H2	0.005156556	2.10	4.67	2.22	0.83	0.69	0.96
cg12105108	18	METTL4; NDC80	2.90233E-06	2.03	8.66	4.26	0.75	0.58	0.91
cg03379367	16	USP31	1.56181E-39	3.26	15.71	4.82	0.86	0.74	0.99
cg25212146	18	DSEL	6.36992E-06	2.61	6.17	2.36	0.75	0.59	0.91
cg10929349	17	SCO1	0.041664912	2.36	3.05	1.29	0.80	0.65	0.95
cg04835163	1	TNNI3K; FPGT; LRRIQ3	0.000624565	2.17	5.52	2.54	0.78	0.63	0.94
cg18191664	8	ERLIN2	4.86294E-09	7.63	5.03	0.66	0.81	0.66	0.95
cg24301192	12	HRK	1.03213E-38	2.17	16.61	7.64	0.82	0.69	0.96
cg01316535	19	ZNF121	0.028020396	2.64	2.98	1.13	0.75	0.59	0.91
cg05094046	18	DYM	6.5469E-05	2.03	7.21	3.56	0.75	0.59	0.91
cg08953194	19	GLTSCR1	4.13396E-11	4.71	6.63	1.41	0.83	0.69	0.96
cg19766441	4	SLC34A2	2.50245E-39	3.60	14.41	4.00	0.82	0.68	0.96
cg10500461	5	PPARGC1B	0.028692296	3.22	2.61	0.81	0.81	0.67	0.95
cg13767695	16	CDH15	8.40647E-05	2.59	5.34	2.06	0.84	0.71	0.97
cg25445017	15	CTXN2	2.36351E-07	2.38	7.95	3.34	0.81	0.67	0.95
cg11443159	19	IRGQ; ZNF576	2.11886E-38	2.37	14.22	6.01	0.77	0.61	0.92
cg11335495	1	GPN2	0.009907084	2.19	4.10	1.87	0.80	0.65	0.95
cg10784843	12	ORAI1	0.002119436	2.12	5.07	2.39	0.81	0.67	0.96
cg22824647	19	MAP1S	0.000914837	2.04	5.80	2.84	0.75	0.58	0.91
cg16023162	4	PRKG2	8.23579E-06	2.28	6.99	3.06	0.75	0.59	0.91
cg26054395	1	MTF2	5.77515E-06	2.58	6.27	2.43	0.76	0.60	0.92
cg24994173	5	TSLP	1.7301E-39	4.94	13.53	2.74	0.78	0.63	0.93
cg27541540	12	PTPN11	5.41449E-38	9.29	8.07	0.87	0.79	0.65	0.94
cg06889484	19	MPV17L2	0.007365739	2.46	3.79	1.54	0.76	0.60	0.92
cg24114314	19	ZNF677	0.002169983	2.12	5.06	2.38	0.77	0.61	0.92
cg17369088	17	CCDC55	5.50773E-41	2.59	22.95	8.84	0.78	0.63	0.93
cg12804420	17	ATAD5	1.74052E-13	2.36	12.65	5.35	0.78	0.63	0.94
cg07917502	2	PREPL	0.001292759	3.72	3.45	0.93	0.75	0.58	0.91
cg24401310	15	MTMR15	0.003051997	2.62	3.96	1.51	0.77	0.61	0.92
cg00986824	12	KCNC2	0.00049257	2.10	5.89	2.81	0.75	0.58	0.91
cg06554331	10	FAM21A	0.026711897	2.22	3.51	1.58	0.78	0.63	0.94
cg20573562	11	SERGEF	0.037318234	2.08	3.57	1.72	0.81	0.67	0.96
cg14195684	8	STAU2	1.16576E-13	3.22	9.42	2.92	0.75	0.58	0.91
cg18701590	5	PRR7	0.012257701	2.00	4.46	2.23	0.75	0.59	0.91
cg12284567	17	PSMC3IP	0.048351844	2.40	2.93	1.22	0.79	0.64	0.94
cg22219278	5	LMAN2	0.038011248	2.43	3.03	1.25	0.82	0.69	0.96
cg15078479	14	PARP2; RPPH1	0.011605415	2.67	3.35	1.25	0.75	0.59	0.91
cg02824451	19	SLC7A10	4.14825E-39	4.05	13.15	3.24	0.81	0.66	0.95
cg08134704	13	GTF2F2	0.046046609	2.14	3.31	1.55	0.83	0.70	0.97
cg09114151	11	EED	0.000832584	2.21	5.27	2.38	0.87	0.75	0.99
cg24006384	1	PARK7	0.001506249	3.04	3.80	1.25	0.75	0.58	0.91
cg18154214	16	PSMD7	8.05112E-12	3.52	8.01	2.27	0.76	0.60	0.92

Supplementary Table S2:

Target ID	CHR	Genes	FDR p-Val	Fold change	% Methylation		AUC	CI	
					Cases	Controls		lower	upper
cg22700691	10	C10orf140	0.000463149	2.56	4.78	1.87	0.76	0.60	0.92
cg05855489	10	C10orf26	1.18197E-39	2.44	18.92	7.76	0.82	0.68	0.96
cg05596319	10	C10orf88	6.21568E-10	2.95	8.04	2.72	0.77	0.61	0.92
cg21156771	11	C11orf73	3.77811E-10	3.19	7.70	2.42	0.81	0.67	0.95
cg18369257	11	C11orf9	3.21742E-06	3.41	5.25	1.54	0.79	0.64	0.94
cg16704476	12	C12orf23	3.9358E-13	2.05	15.39	7.52	0.78	0.63	0.94
cg26229977	12	C12orf57	6.71069E-07	2.37	7.63	3.23	0.84	0.70	0.97
cg16091543	12	C12orf65	5.13575E-08	3.43	6.23	1.82	0.81	0.66	0.95
cg15901216	14	C14orf102	1.91243E-10	3.97	6.88	1.73	0.83	0.69	0.97
cg08165558	14	C14orf167	1.11216E-05	2.10	7.68	3.66	0.76	0.60	0.92
cg18498159	15	C15orf17	4.66236E-12	3.12	8.83	2.83	0.76	0.60	0.92
cg26884816	15	C15orf39	0.002213234	2.73	3.95	1.45	0.76	0.60	0.92
cg08251337	16	C16orf61	5.89982E-05	2.03	7.23	3.56	0.80	0.66	0.95
cg25674838	17	C17orf105	6.59164E-06	3.48	5.01	1.44	0.81	0.67	0.95
cg01906998	17	C17orf106	0.00137692	2.41	4.60	1.91	0.81	0.67	0.96
cg13258989	17	C17orf28	8.30075E-06	2.69	5.93	2.21	0.83	0.69	0.96
cg10929349	17	C17orf48	0.041664912	2.36	3.05	1.29	0.80	0.65	0.95
cg23261070	17	C17orf49	0.001020992	2.94	4.02	1.37	0.80	0.65	0.95
cg22700039	17	C17orf51	2.75772E-40	2.07	24.37	11.79	0.80	0.65	0.95
cg14576294	17	C17orf61	0.010312747	2.86	3.24	1.13	0.75	0.59	0.91
cg14595275	17	C17orf72	0.000122042	2.30	5.87	2.56	0.77	0.61	0.92
cg02840638	17	C17orf76	0.002150033	2.02	5.43	2.69	0.81	0.66	0.95
cg12500437	17	C17orf80	1.48148E-09	4.04	6.42	1.59	0.75	0.59	0.91
cg12202743	18	C18orf22	0.029421304	2.32	3.31	1.43	0.84	0.71	0.97
cg01968530	18	C18orf45	0.003526641	2.04	5.08	2.50	0.75	0.59	0.91
cg03766516	19	C19orf29	0.007592812	2.92	3.31	1.13	0.88	0.77	1.00
cg01037673	19	C19orf39	0.013222805	2.10	4.13	1.96	0.86	0.74	0.99
cg26886427	19	C19orf52	6.25909E-12	2.84	9.48	3.34	0.76	0.60	0.92
cg18886719	19	C19orf53	1.28962E-08	2.85	7.48	2.63	0.89	0.78	1.00
cg12571879	19	C19orf59	2.05108E-09	2.58	8.75	3.40	0.82	0.69	0.96
cg07697134	19	C19orf62	2.65424E-13	2.05	15.54	7.59	0.76	0.60	0.92
cg05344495	19	C19orf77	1.0135E-11	2.25	12.13	5.39	0.76	0.60	0.92
cg17225465	1	C1orf212	1.35372E-38	2.37	15.03	6.35	0.84	0.71	0.97
cg16306898	1	C1orf70	2.74541E-11	3.89	7.33	1.88	0.85	0.71	0.98
cg12858300	1	C1orf74	4.39704E-05	2.75	5.29	1.92	0.75	0.59	0.91
cg27312944	1	C1orf86	1.10954E-08	2.46	8.67	3.53	0.87	0.75	0.99
cg20777508	20	C20orf114	6.38976E-12	4.25	7.28	1.71	0.77	0.62	0.93
cg02480104	20	C20orf132	0.001385512	2.97	3.89	1.31	0.78	0.63	0.94
cg17234198	20	C20orf199	6.76865E-06	3.42	5.06	1.48	0.76	0.60	0.92
cg06510830	21	C21orf45	0.005924677	2.05	4.74	2.31	0.77	0.61	0.92
cg25314532	22	C22orf23	0.002644531	2.14	4.90	2.29	0.75	0.59	0.91
cg07596120	22	C22orf32	1.1514E-05	2.43	6.41	2.64	0.83	0.69	0.97
cg05570707	2	C2orf44	3.50123E-14	2.11	15.52	7.35	0.77	0.62	0.93
cg19233043	3	C3orf18	1.23008E-05	3.28	5.03	1.53	0.81	0.66	0.95
cg02758599	3	C3orf21	6.09162E-11	2.22	11.70	5.26	0.75	0.59	0.91
cg22993707	3	C3orf31	5.9831E-06	4.59	4.44	0.97	0.86	0.74	0.99

cg24826502	3	C3orf37	0.001926272	2.14	5.09	2.38	0.81	0.66	0.95
cg16671114	3	C3orf58	0.002335892	3.25	3.51	1.08	0.79	0.64	0.94
cg10515332	4	C4orf37	7.47883E-06	2.51	6.36	2.54	0.76	0.60	0.92
cg21127184	4	C4orf41	1.06861E-44	2.07	41.24	19.88	0.78	0.63	0.94
cg04522604	5	C5orf13	1.40483E-09	2.03	12.17	6.00	0.84	0.71	0.97
cg04124112	5	C5orf24	7.48199E-39	3.10	13.72	4.42	0.85	0.73	0.98
cg03866757	5	C5orf41	3.97323E-05	3.13	4.85	1.55	0.75	0.59	0.91
cg08904082	5	C5orf43	1.51275E-06	3.51	5.35	1.52	0.78	0.62	0.93
cg04059965	5	C5orf45	0.000736428	3.53	3.71	1.05	0.83	0.70	0.97
cg07816366	6	C6orf134	0.004759907	2.67	3.72	1.40	0.76	0.60	0.92
cg19477921	6	C6orf174	3.74083E-09	2.04	11.63	5.70	0.75	0.59	0.91
cg05955802	6	C6orf204	0.00755663	2.05	4.61	2.25	0.76	0.60	0.92
cg11371403	9	C9orf135	4.14224E-08	2.62	7.73	2.95	0.75	0.58	0.91
cg05338745	12	C12orf26	2.10056E-05	6.12	3.80	0.62	0.77	0.61	0.93
cg05279284	14	C14orf21	0.011872967	2.52	3.50	1.39	0.77	0.61	0.92
cg05386387	18	C18orf55	0.003054209	4.65	2.92	0.63	0.85	0.72	0.98
cg14109663	19	C19orf70	1.54679E-06	3.95	5.03	1.27	0.86	0.73	0.98
cg14614793	9	C9orf6	4.72854E-41	6.12	17.02	2.78	0.81	0.67	0.96
cg04343292	1	C1orf174	6.1472E-08	2.46	8.09	3.28	0.84	0.71	0.97
cg06700856	18	C18orf18	0.003617756	2.61	3.90	1.50	0.75	0.59	0.91
cg26945813	10	C10orf114	2.02145E-11	2.38	11.07	4.66	0.81	0.66	0.95
cg00104570	3	C3orf26	0.037166153	3.55	2.38	0.67	0.75	0.59	0.91
cg24737639	12	C12orf48	0.022361072	2.12	3.78	1.78	0.76	0.60	0.92
cg12635019	15	C15orf56	0.011507136	2.26	3.89	1.72	0.82	0.68	0.96
cg07917502	2	C2orf34	0.001292759	3.72	3.45	0.93	0.75	0.58	0.91
cg13614659	20	C20orf72	1.73522E-06	2.23	7.80	3.49	0.75	0.58	0.91
cg09703789	6	C6orf225	0.015461548	2.15	3.95	1.84	0.78	0.63	0.93
cg06030942	14	C14orf119	1.86281E-13	4.06	8.08	1.99	0.84	0.71	0.97
cg20648939	18	C18orf19	0.045635078	2.20	3.22	1.46	0.78	0.62	0.93
cg26831488	11	C11orf71	0.027317358	2.42	3.22	1.33	0.78	0.63	0.93
cg27063739	9	C9orf163	0.033659762	2.09	3.60	1.72	0.78	0.63	0.94
cg20876723	17	C17orf91	0.044304834	2.51	2.87	1.14	0.75	0.59	0.91
cg15524173	19	C19orf47	0.005204489	2.72	3.63	1.34	0.78	0.63	0.94
cg20415529	4	C4orf14	3.23923E-39	2.03	20.05	9.90	0.87	0.75	0.99
cg10207042	12	C12orf4	0.018816068	2.03	4.10	2.02	0.78	0.63	0.93

Supplementary Table S3:

Target ID	CHR	Gene	FDR p-Val	Fold change	% Methylation		AUC	CI	
					Cases	Controls		lower	upper
cg21816532	17	LOC404266	2.21E-08	2.13	10.15	4.77	0.89	0.78	1.00
cg23065793	3	LOC100128164	1.18E-12	3.85	7.97	2.07	0.88	0.76	0.99
cg21933829	16	LOC101927814	2.60E-02	2.07	3.82	1.85	0.87	0.75	0.99
cg27312944	1	LOC100128003	1.11E-08	2.46	8.67	3.53	0.87	0.75	0.99
cg12749132	1	LOC100132111	2.82E-06	5.75	4.27	0.74	0.87	0.75	0.99
cg21348554	1	LOC643355	8.13E-05	2.70	5.17	1.92	0.86	0.74	0.99
cg04127233	4	LOC101927179	2.13E-38	3.57	11.40	3.20	0.86	0.74	0.99
cg19580633	5	LOC100268168	1.82E-08	4.54	5.62	1.24	0.85	0.73	0.98
cg04343292	1	LOC100133612	6.15E-08	2.46	8.09	3.28	0.84	0.71	0.97
cg01754828	6	LOC101928307	2.23E-14	2.03	16.72	8.22	0.83	0.70	0.97
cg10800082	10	LOC282997	5.63E-39	2.57	15.71	6.12	0.83	0.70	0.97
cg22905859	1	LOC728855	6.48E-07	3.26	5.82	1.79	0.83	0.69	0.97
cg05009619	7	LOC100134713	1.75E-02	2.12	3.93	1.85	0.83	0.69	0.96
cg06187252	5	LOC731157	1.68E-04	2.13	6.30	2.96	0.81	0.67	0.96
cg15732840	2	LOC100132215	1.95E-09	2.96	7.73	2.61	0.81	0.67	0.95
cg03467424	10	LOC102031319	2.65E-02	3.09	2.70	0.87	0.80	0.66	0.95
cg19515530	4	LOC550112	1.18E-07	3.47	5.99	1.73	0.80	0.65	0.95
cg18330637	20	LOC388789	1.52E-03	2.49	4.42	1.77	0.80	0.65	0.94
cg09552183	12	LOC101928597	6.03E-07	4.01	5.21	1.30	0.80	0.65	0.94
cg18029778	7	LOC100216545	7.55E-41	3.14	20.25	6.45	0.79	0.64	0.94
cg12518410	2	LOC100287216	2.44E-07	4.86	4.99	1.03	0.79	0.64	0.94
cg03213877	1	LOC643837	1.57E-13	3.82	8.38	2.20	0.79	0.64	0.94
cg25635304	16	LOC100505942	4.25E-39	2.15	18.46	8.59	0.79	0.64	0.94
cg01050704	19	LOC100131691	8.50E-03	2.33	3.92	1.68	0.79	0.64	0.94
cg24626312	17	LOC404266	5.46E-05	3.35	4.57	1.36	0.79	0.64	0.94
cg19125926	11	LOC101929089	2.39E-40	4.83	16.05	3.32	0.79	0.64	0.94
cg15709401	20	LOC100270804	1.80E-12	7.28	6.25	0.86	0.79	0.64	0.94
cg00140842	2	LOC100302652	2.16E-07	2.24	8.57	3.82	0.78	0.63	0.94
cg03301945	19	LOC147727	1.95E-06	2.57	6.64	2.58	0.78	0.63	0.94
cg17448127	4	LOC285456	3.60E-07	2.92	6.43	2.20	0.78	0.63	0.93
cg11208853	12	LOC653113	3.51E-03	3.83	3.10	0.81	0.78	0.63	0.93
cg25631714	4	LOC550112	2.45E-03	2.06	5.21	2.54	0.77	0.62	0.93
cg05476169	22	LOC646851	1.03E-03	3.56	3.59	1.01	0.77	0.62	0.93
cg22811818	3	LOC440944	2.55E-03	2.28	4.59	2.01	0.77	0.61	0.92
cg20686479	11	LOC100126784	4.18E-04	2.31	5.32	2.30	0.77	0.61	0.92
cg18026227	8	LOC100192378	6.14E-15	2.62	12.14	4.63	0.77	0.61	0.92
cg23202277	17	LOC100128977; LOC100130148	4.46E-06	2.68	6.15	2.30	0.76	0.60	0.92
cg08838040	7	LOC100507642	1.61E-05	2.22	6.96	3.13	0.75	0.59	0.91
cg00719400	1	LOC100130093	3.55E-05	2.41	6.05	2.51	0.75	0.59	0.91
cg06700856	18	LOC339290	3.62E-03	2.61	3.90	1.50	0.75	0.59	0.91
cg20978010	12	LOC338799	2.50E-04	3.73	3.91	1.05	0.75	0.59	0.91

Supplementary Figure 1A.

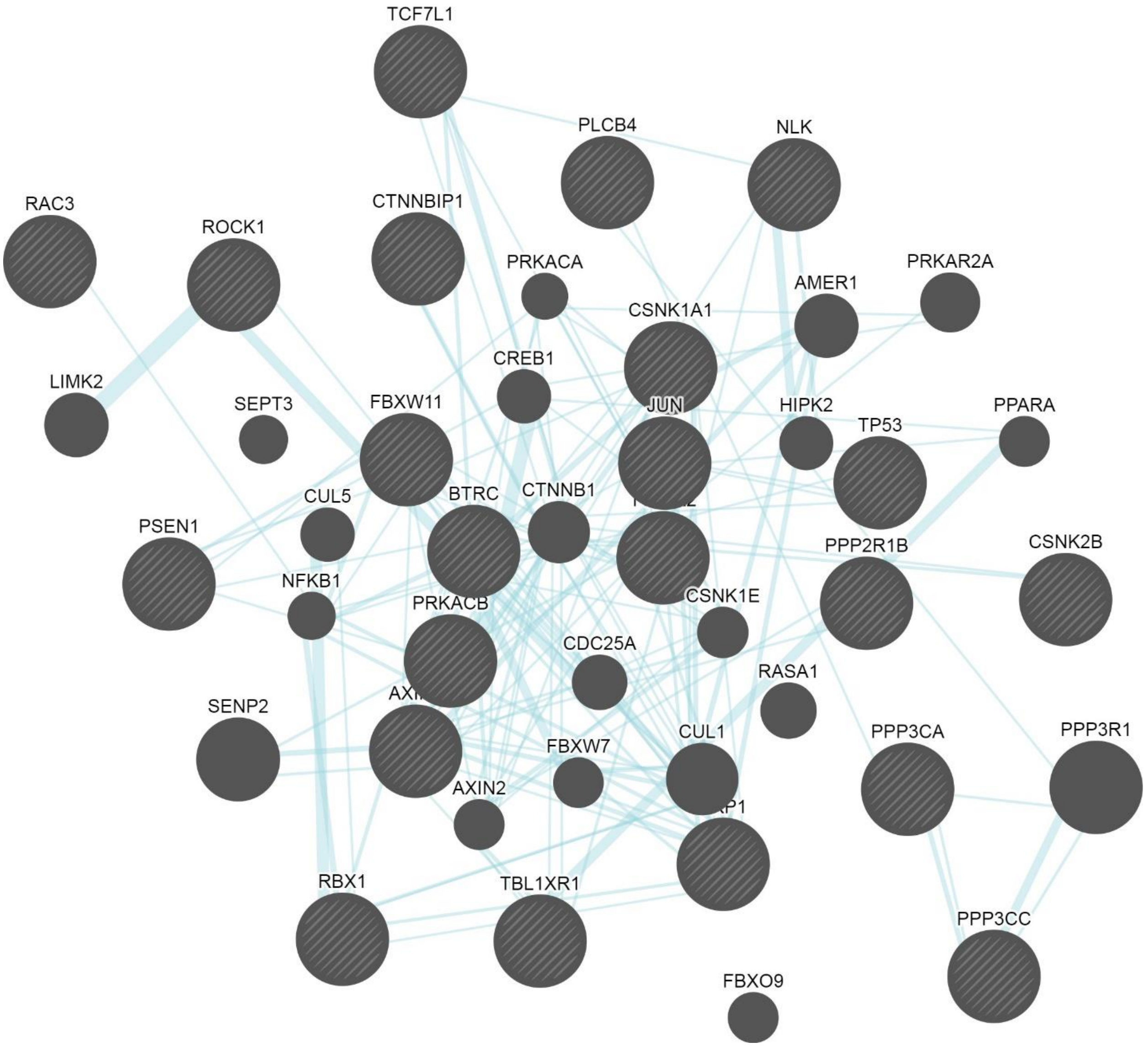

Supplementary Figure 1B

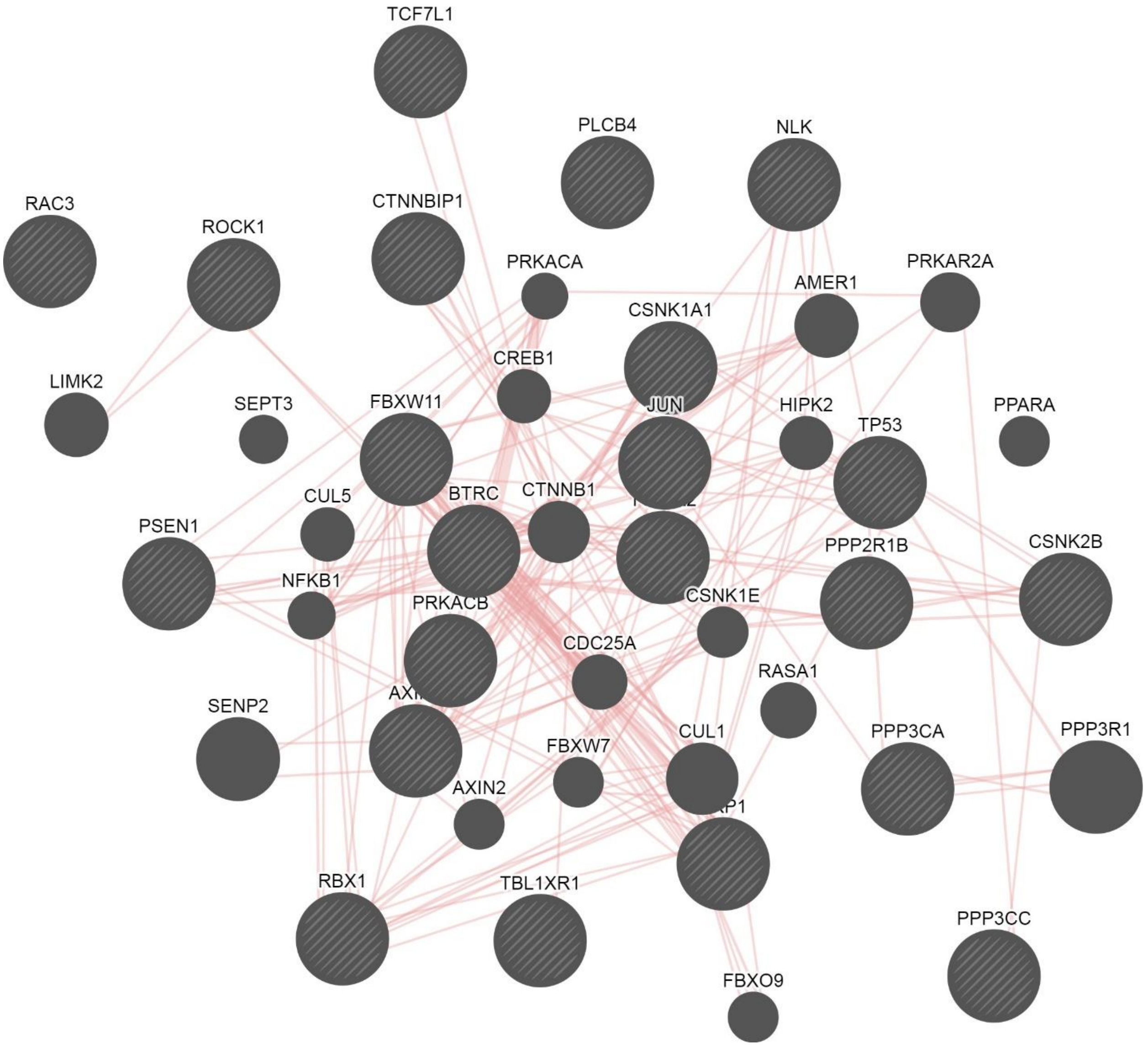

Supplement: Supplementary file 1 — Dataset 1 [file 41598_2019_40299_MOESM1_ESM.pdf]
